# Deuteration of Arenes via Pd-Catalyzed C–H Activation: A Lesson in Nondirected C–H Activation, Isotopic Labeling, and NMR Characterization

**DOI:** 10.1021/acs.jchemed.4c00270

**Published:** 2024-07-16

**Authors:** Fritz Deufel, Manuel van Gemmeren

**Affiliations:** Otto-Diels-Institut für Organische Chemie, Christian-Albrechts-Universität zu Kiel, Otto-Hahn-Platz 4, 24118 Kiel, Germany

**Keywords:** Upper-Division Undergraduate, Laboratory Instruction, Catalysis, Deuteration, C−H Activation, Arenes, Late-Stage Functionalization

## Abstract

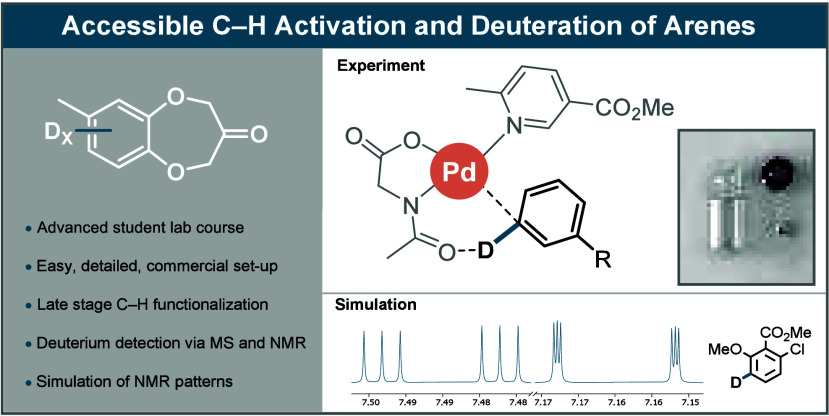

Isotopic labeling is an important tool in medicinal research, metabolomics, and for understanding reaction mechanisms. In this context, transition metal-catalyzed C–H activation has emerged as a key technology for deuterium incorporation via hydrogen isotope exchange. A detailed and easy-to-implement experimental procedure for a nondirected arene deuteration has been developed that exclusively uses commercial equipment and chemicals. The protocol is ideally suited for students and other prospective applicants who are not experts in catalysis. The degree of deuterium incorporation was analyzed via different means like mass spectrometry and ^1^H and ^2^H nuclear magnetic resonance (NMR). A hands-on understanding of quantitative NMR, as well as the influence of H/D exchange on experimental spectra, was conveyed by comparative NMR spin simulations. Students were measurably familiarized with the concepts of C–H activation, isotope effects, and basics in experimental catalysis.

## Introduction

Deuterium incorporation alters the properties of a native reference molecule and has therefore gained increasing attention in medicinal chemistry and materials research. Changes in the behavior of administration, distribution, metabolism, and excretion (ADME), solubility, or fluorescence lifetime have been documented. Additionally, metabolomics studies harness the different isotopic distributions to identify deuterium marked metabolites, and studies of reaction mechanisms often require a positional marker or a heavy isotopologue for investigating kinetic isotope effects.^1^

**Figure 1:**
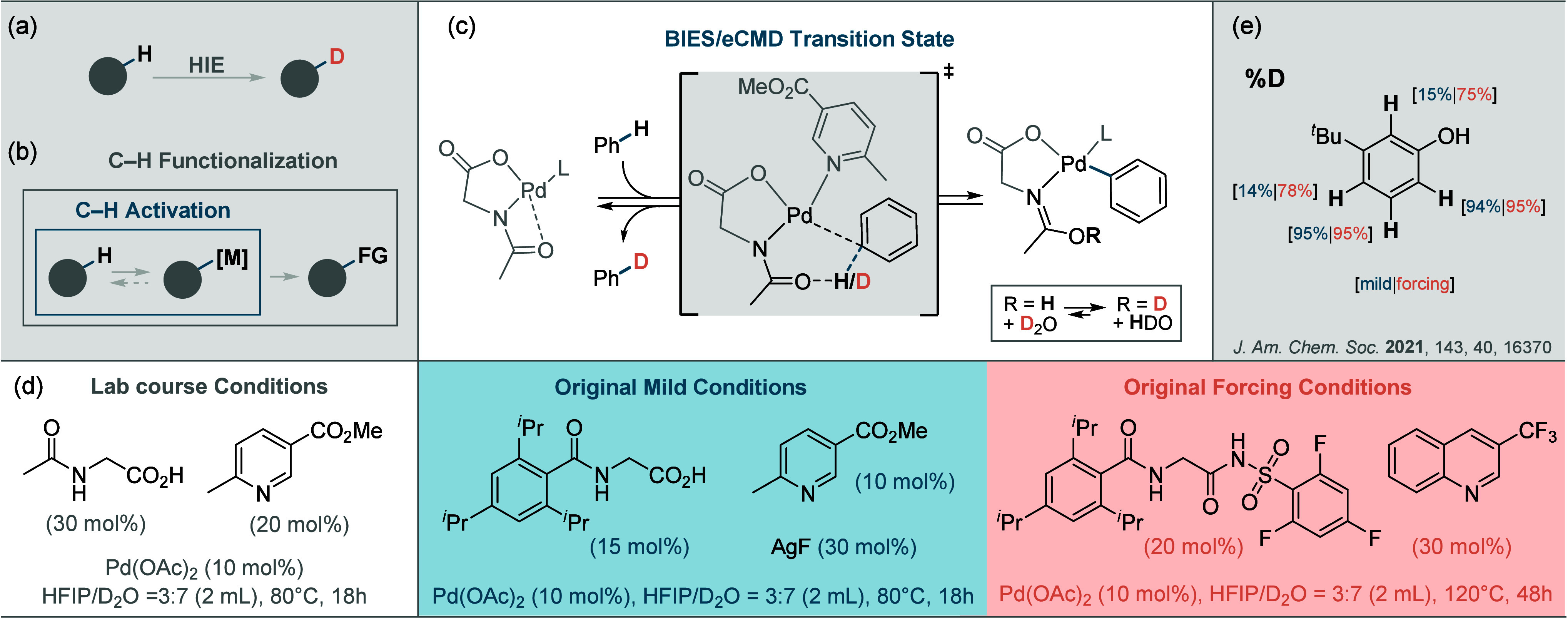
Schematic overview of (a) HIE and (b) C–H activation/functionalization. (c) Dual ligand-enabled transition state and simplified deuterium incorporation based on the reversibility of the C–H activation step. (d) Overview of the lab course conditions in comparison with the optimized mild and forcing literature reaction conditions. (e) Representative example of deuterium incorporation (%D) in a model substrate under mild and forcing literature conditions.

Synthetically, hydrogen isotope exchange (HIE) has emerged as a key technique to access deuterated analogues since it does not rely on the prefunctionalization of a target molecule and directly exchanges hydrogen and deuterium (Figure 1a).^2^ Different approaches make use of Lewis acid/base catalysis or transition metal-catalyzed C–H activation and have extensively been reviewed in the literature.^3^ C–H activation describes an elementary step in organometallic chemistry, in which a C–H bond is converted to a C–transition metal bond through an inner sphere mechanism. The overall process in which the C–H bond is converted to a functional group (often via transition metal catalysis) is termed C–H functionalization (Figure 1b).

**Figure 2:**
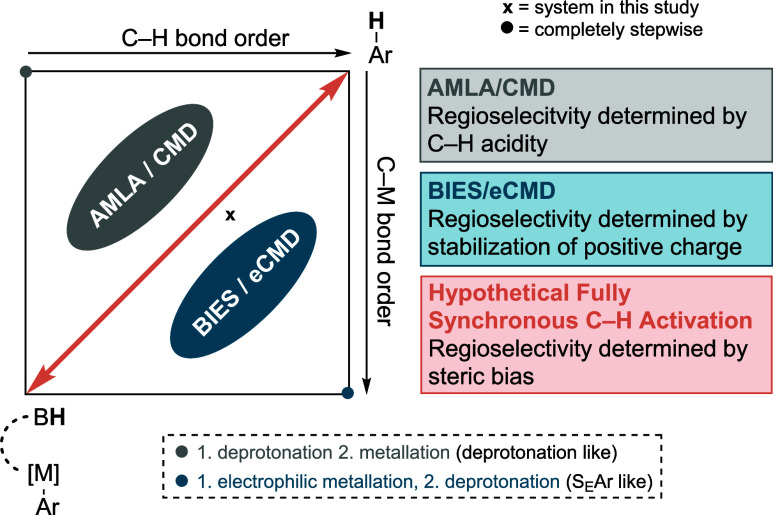
More O’Ferrall–Jencks plot illustrating the different mechanistic scenarios for a concerted C–H activation in a continuum ranging from S_E_Ar to deprotonation.

A hydrogen/deuterium (H/D) exchange can occur through C–H activation and its microscopic reverse in the presence of a deuterium source, the deutero-demetalation. Simply speaking, the organometallic species generated after the C–H activation step can exchange a proton for deuterium and revert back to the (now deuterated) starting material (Figure 1c). In the case of homogeneous C–H activation, concerted mechanisms prevail, which are in varying degrees sensitive to steric and electronic effects and have been reviewed extensively in the literature.^4^ Ambiphilic metal–ligand activation (AMLA) or concerted metalation deprotonation (CMD) mechanisms activate preferentially the most acidic position. An electronic preference for electron-rich positions can be rationalized by a concerted yet asynchronous mechanism often termed base-assisted intramolecular electrophilic-type substitution (BIES) or electrophilic concerted metalation deprotonation (eCMD), resulting in regioselectivities resembling those of S_E_Ar-type reactions. The more synchronous a C–H activation mechanism becomes, the weaker electronic effects become, and consequently, steric effects play an increasing role in such cases. The system applied herein shows electronic preference and steric bias typical for a comparably synchronous eCMD/BIES-type mechanism.^5^ The More O’Ferrall–Jencks diagram (Figure 2) is used in the literature^5^ to illustrate these different mechanistic scenarios as a function of the extent to which the C–H bond is cleaved (decreasing C–H bond order) and the extent to which a new C–M bond is formed (increasing C–M bond order). Deprotonation and S_E_Ar are extreme (stepwise rather than concerted) cases along the edges of the diagram, whereas AMLA/CMD and BIES/eCMD are all concerted yet asynchronous, tending to one of these extremes, respectively.

From a synthetic perspective, C–H activation offers the additional advantage that it is in principle suitable for late-stage functionalization (LSF), underscoring its relevance in labeling and medicinal chemistry. Here, LSF refers to chemoselective, functional group tolerant transformations that do not require a prefunctionalization of the substrate molecules.^6,7^ The recently disclosed nondirected C–H activation procedures by Fernández-Ibáñez,^8^ Yu,^9^ and van Gemmeren^10^ do not require directing groups, can use the arene as the limiting reagent,^11^ and are in principle suited for LSF. Late-stage deuteration is especially attractive since a complex organic molecule at hand can be transformed to its deuterated analogue in one benign step without the need of developing a new diverging multistep synthesis. Classical routes that involve steps such as halogenation, lithiation, and lithium deuterium exchange are comparatively step intensive and are often incompatible with, for example, base sensitive functional groups. It can be highly advantageous to rapidly access deuterated bioactive compounds in the context of ADME studies, where the distinct isotope patterns enable monitoring of the concentration and identity of a bioactive compound under investigation and its metabolites.

The increasing importance of deuteration and C–H activation in isotopic labeling inspired us to design a laboratory experiment based on a modified study from our group (Figure 1e).^12^ The dual ligand-based catalyst design^13,14^ is maintained but is adapted to use exclusively commercially available equipment, starting materials, and ligands.^15^

## Experimental Overview

### Experimental Tasks

**Figure 3:**
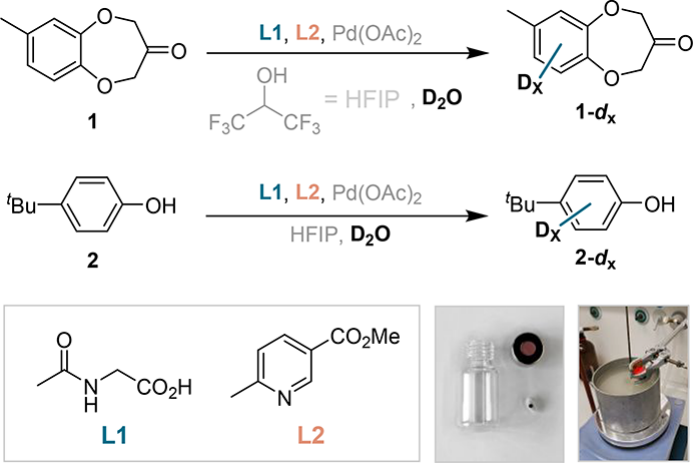
Deuteration of watermelon ketone **1** and 4-*tert*-butylphenol **2** using D_2_O as the deuterium source. The reactions were performed on a 0.2 mmol scale with **L1** (20 mol %), **L2** (30 mol %), and Pd(OAc)_2_ (10 mol %) in HFIP:D_2_O = 3:7 (1 M), 80 °C, 18 h.

After an initial risk assessment, the students were tasked to carry out two catalytic deuteration experiments using watermelon ketone **1**, which has a pronounced aquatic smell, and 4-*tert*-butylphenol **2** (Figure 3). Palladium(II) acetate is used as a catalyst precursor, *N*-acetylglycine (**L1**) as a bidentate ligand, and methyl 6-methynicotinate (**L2**) as a monodentate ligand. A solvent mixture of 1,1,1,3,3,3-hexafluoropropan-2-ol (HFIP) and D_2_O is crucial for the desired reactivity and functions as the deuterium source (for details, see the Supporting Information (SI)). The students ran the reactions overnight and analyzed the crude reaction mixtures using NMR and GC-MS. An internal standard was used to quantify the reaction yield via ^1^H NMR.

The students were initially tasked to determine the degree of deuteration using mass spectrometry (MS). The altered isotopic contribution compared to the native molecules is reflected in the relative intensities of the MS peaks corresponding to the target molecule. A higher degree of deuterium incorporation translates to an increased signal intensity of heavier mass peaks. The Universal Mass Calculator^16^ and the integrated fitting algorithm allow for a determination of the overall deuterium content. The deuterium content was compared with the results from quantitative ^1^H NMR spectroscopy. Here, the decreased signal intensity in aromatic ^1^H signals correlates to the replacement of ^1^H by ^2^H. The students were given spectra of the starting materials to assign the chemical shift to the respective positions and determine the positional selectivity and degree of deuteration in each position (%D). For the starting material **1**, two-dimensional NMR spectra (COSY, HMBC, HSQC) were provided to ensure an unambiguous assignment of chemical shifts and to refamiliarize students with the practical analysis of 2D spectra. Principles of quantitative NMR (qNMR) were further illustrated at this stage by comparing a spectrum with insufficiently long relaxation delay (*d*1 = 0.1 s) with a sufficiently relaxed spectrum (*d*1 = 30 s). With this knowledge at hand, the crude yield was determined with respect to 1,3,5-trimethoxybenzene (TMB) as internal standard.

In addition to ^1^H NMR spectra, standard proton decoupled ^13^C NMR spectra (^13^C{^1^H} for **1-*****d***_**X**_) and ^2^H NMR spectra (for **1-*****d***_**X**_) were recorded and compared with the native compound. The influence of ^2^H on the ^13^C NMR signals was investigated by the students, and the location of deuterium incorporation was further verified by ^2^H NMR.

### Post-Laboratory Tasks

In order to convey detailed knowledge regarding the effect of deuterium incorporation on NMR spectra, the students were tasked to simulate the spin system and coupling pattern of compound **2** and its *ortho* dideuterated analogue (**2-*****d***_**2**_) and to compare the simulation with the experimental spectra (**2-*****d***_**X**_) using MNova.^17^

Additionally, selected tri- and tetrasubstituted arenes were simulated. To this end, the given coupling constants for *J*_o_, *J*_m_, and *J*_p_ needed to be scaled for the deuterated analogues (*J*_H,H_ ≈ 6.5*J*_H,D_). Additional theoretical questions (see the SI) were to be answered in the final report.

## Hazards

Standard safety precautions should be taken (lab goggles, lab coat, gloves). The use of a well-ventilated fume hood is essential. The pressurized vial should be allowed to cool to room temperature before opening (not flammable but potential overpressure). EtOAc is flammable. Nitric acid and hydrochloric acid are corrosive and can liberate toxic vapors (NO_*x*_); hence, appropriate dilution and safety gloves are vital. Needles are to be disposed of in puncture resistant containers. HFIP and 4-*tert*-butylphenol should be handled with special care.

A more comprehensive safety assessment and detailed precautionary measures on handling and waste disposal are provided in the supplemental safety data sheet (see the SI).

## Results and Discussion

When designing this lab course, we identified the following main learning goals: a deeper understanding of deuterium labeling, C–H activation, and a familiarization with the experimental and technical equipment required in state-of-the-art research in the respective fields. The following specific points illustrate the goals in more detail.

### Learning Outcomes

- Practical introduction to C–H activation, C–H deuteration, and late-stage functionalization
- Determination of deuteration degrees using mass spectrometry and qNMR
- Simulation of NMR spectra of deuterated organic compounds (isotopologues)
- Visual determination of the parameters influencing a spectrum

### Results

**1** and **2** were deuterated according to the experimental procedure. Compound **1** was obtained in 72% yield after deuteration. The NMR and MS values indicate a moderate total deuterium incorporation of one ^2^H atom per molecule on average.^18^

**Figure 4:**
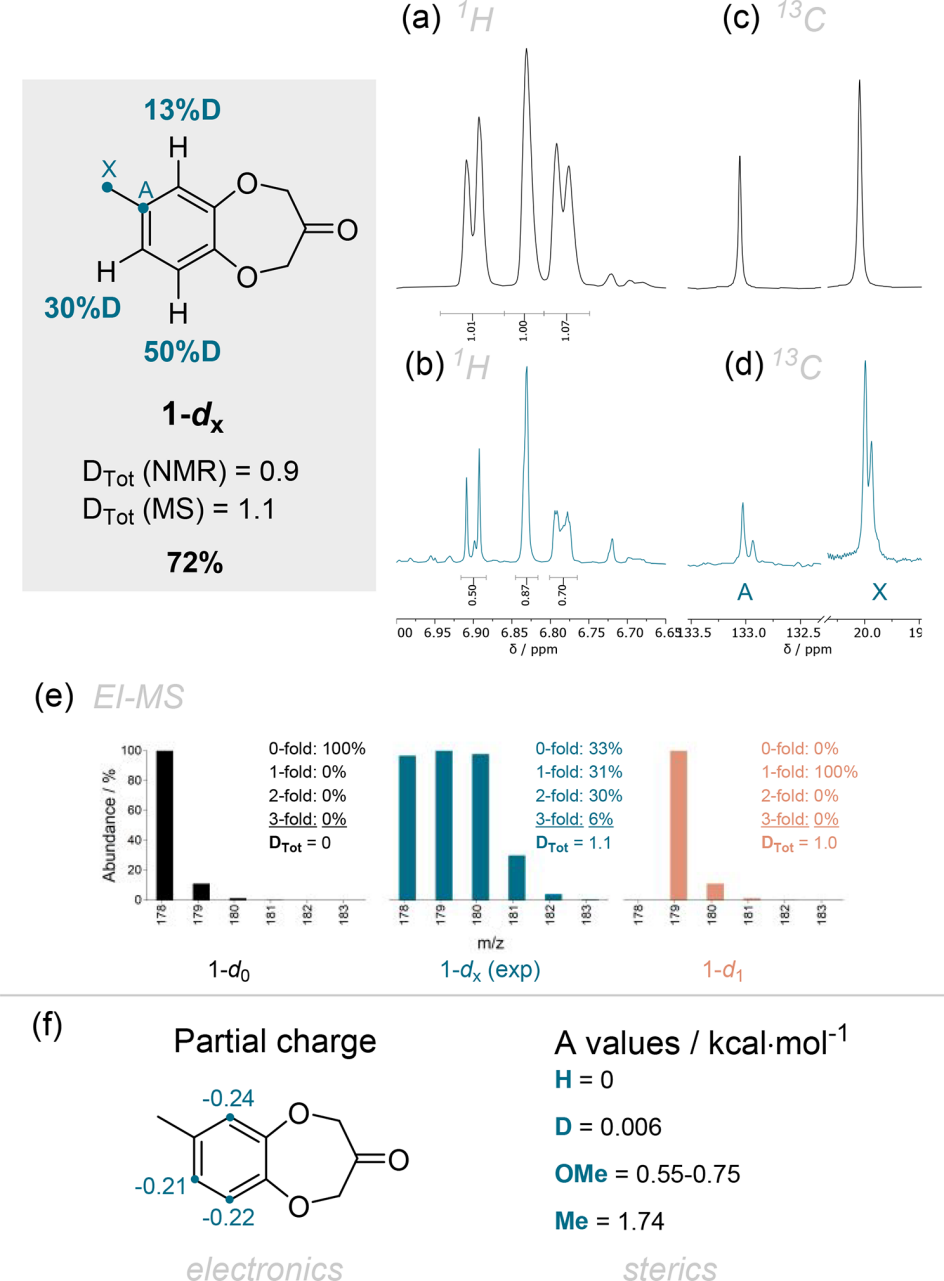
Experimental results from the deuteration of **1** with the crude yield using TMB as internal standard and the total degree of deuteration *D*_Tot_ from ^1^H NMR and MS measurements. An overlay is shown of (a) the ^1^H experimental spectrum of the starting material **1**, (b) the crude spectrum of deuterated compound **1-*****d***_**X**_, (c) the ^13^C NMR spectrum of **1** magnified to nuclei A and X, and (d) the ^13^C NMR spectrum of **1-*****d***_**X**_ magnified to nuclei A and X. (e) EI-MS comparison of the [M^+•^] peak of the simulated nondeuterated starting material **1-*****d***_**0**_, the experimentally measured mass pattern of **1-*****d***_**X**_, and a simulated perfectly monodeuterated **1-*****d***_**1**_. (f) Comparison of the NBO partial charges on carbon and *A* values for substituents resembling the ones present in compound **1**.

**Figure 5:**
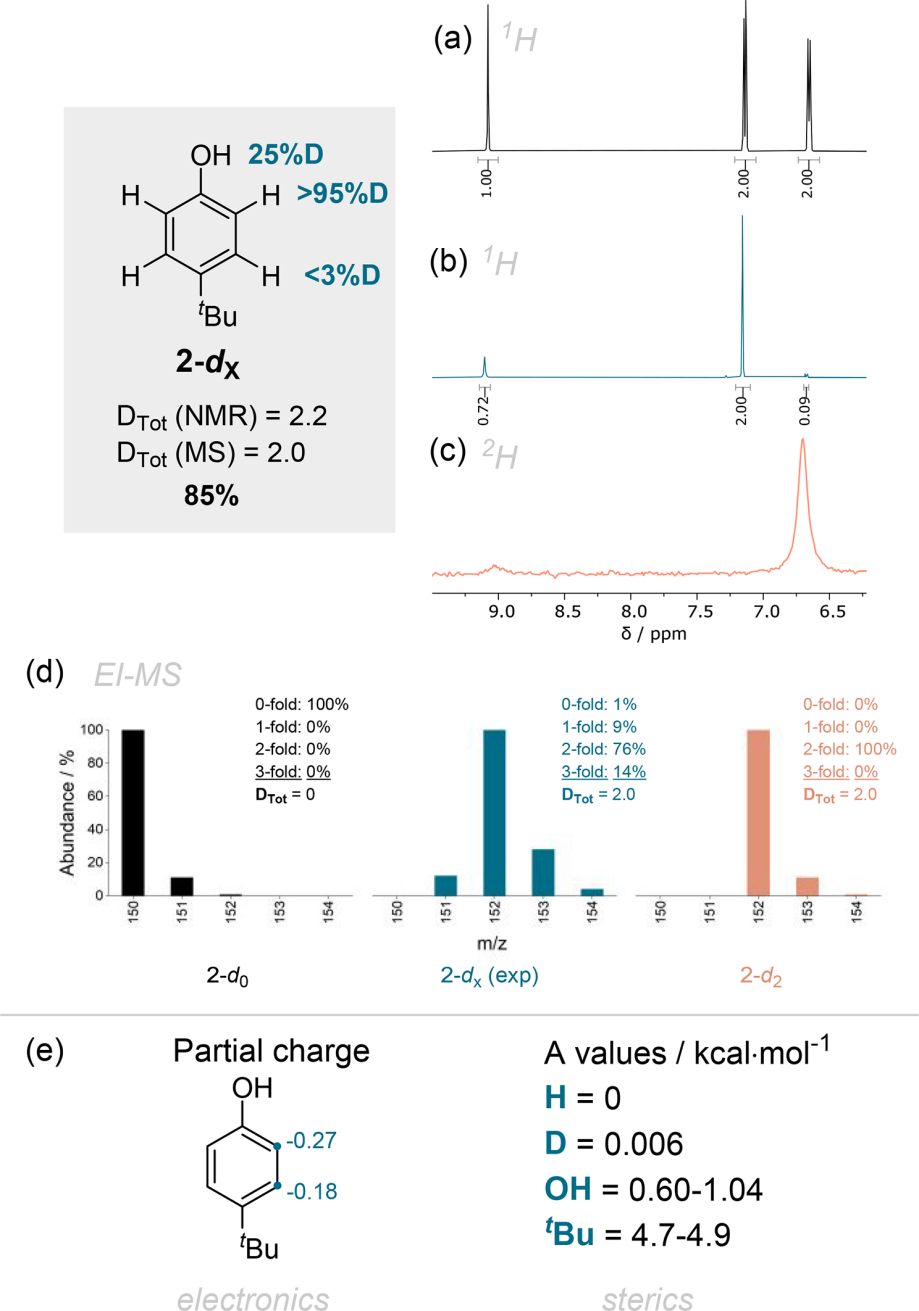
Experimental results from the deuteration of **2** with the crude yield using TMB as internal standard and the total degree of deuteration *D*_Tot_ from ^1^H NMR and MS measurements. An overlay is shown of (a) the ^1^H experimental spectrum of the starting material **2**, (b) the crude spectrum of deuterated compound **2-*****d***_**X**_, and (c) the ^2^H NMR spectrum of **2-*****d***_**X**_. (d) EI-MS comparison of the [M^+•^] peak of the simulated nondeuterated starting material **2-*****d***_**0**_, the experimentally measured mass pattern of **2-*****d***_**X**_, and a simulated perfectly monodeuterated **2-*****d***_**2**_. (e) Comparison of the averaged NBO partial charges on carbon and *A* values for the substituents present in compound **2**.

The EI-MS traces (Figures 4e and 5d) show a distinct change in the isotope pattern from nondeuterated starting material **1-*****d***_**0**_, following the natural isotope distribution (^2^H ≈ 0.012%), to the deuterated product. In the case of **1-*****d***_**X**_, an average total degree of deuteration of *D*_Tot_ (MS) = 1.1 is observed. A broad distribution of the deuterium incorporation between 0- (33%), 1- (31%), 2- (30%), and 3-fold (6%) is observed rather than a selective formation of monodeuterated **1-*****d***_**1**_. This highlights the independence of the C–H activation events in the different positions: in a nondirected reaction, a monodeuterated molecule can react a second time through one of the other reactive positions, leading to a statistical distribution of the deuteration degree, which is superimposed with intrinsic reactivity differences between the respective C–H bonds. Accordingly, a stronger positional preference and higher overall average deuteration degree *D*_Tot_ (MS) = 2.0 in the case of **2-*****d***_**X**_ leads to a narrower isotope distribution (0-fold: 1%; 1-fold: 9%; 2-fold: 76%; and 3-fold: 14%) that approaches the simulated spectrum of the regioselectively dideuterated compound **2-*****d***_**2**_ (2-fold: 100%).

The integration of the ^1^H NMR spectrum was referenced to the four CH_2_ protons and shows a decrease in signal intensity in ^1^H for the deuterated product compared to the reference starting material (Figure 4a–d). **1-*****d***_**X**_ exhibits positional partial deuterium incorporation ranging from 13%D to 50%D. All positions are comparably electron-rich (more negative partial charge, Figure 4e) but have a different steric environment with a methyl, a hydrogen, or an aliphatic ether *ortho* to the respective hydrogen. A simple measure for the steric demand of a functional group is its so-called *A* value. These values are tabulated for many substituents and relate to the influence the substituent has on the conformational equilibrium of cyclohexane, i.e., a larger *A* value indicates a stronger preference to be in an equatorial orientation and hence a bulkier substituent (for a more detailed explanation, see the related literature).^19^ A qualitative comparison of *A* values^20^ (H = 0, OMe (simplified ether) = 0.55–0.75, Me = 1.74 kcal mol^–1^) underlines the trend of more deuterium incorporation at the sterically more accessible position. The positions *ortho* to a methyl group were substituted to a lesser extent (30% vs 53%). The additional steric effect of the methyl and ether group (vs methyl and H) leads to an even more sterically demanding environment (30% vs 13%).

In the ^13^C{^1^H} NMR, additional peaks appear. A ^13^C–^2^H splitting is generally not well resolved (low signal-to-noise ratio, line broadening), and additional peaks from ligands (crude spectra) complicate the analysis. But in Figure 4d, one can see additional small peaks shifted to lower frequencies that arise from the deuterium incorporation in the vicinity of the respective ^13^C nuclei. This so-called isotope shift is due to a change in magnetic environment and hence leads to different chemical shifts.

Compound **2-*****d***_**X**_ was obtained in 85% yield and with an almost complete deuteration *ortho* to the phenolic hydroxy group (>95%, *D*_Tot_ = 2.0). A negligible deuterium content (<3%D) is observed for the protons *ortho* to the ^*t*^Bu group (Figure 5). Here the steric effect is more pronounced (*A* values: OH = 0.60–1.04, ^*t*^Bu = 4.7–4.9 kcal mol^–1^),^21^ and additionally, the hydroxy group is strongly electron donating (+M effect), leading to a pronounced difference in nucleophilicity between *ortho* and *meta* positions (Figure 5d). In DMSO-*d*_6_ OH signals appear sharp, and a partial deuterium incorporation of the hydroxy proton was observed. This is dependent on the workup (variability between experiments) since the phenolic protons are comparably acidic, and an exchange with H_2_O is feasible. The deuterium incorporation was determined via ^1^H NMR using the ^*t*^Bu group as internal reference for integration. This was further corroborated by a ^2^H NMR spectrum, which can be measured in a deuterated solvent if the solvent peak has a sufficient chemical shift difference with the signals of interest.^21^ Since the chemical shift scale of ^1^H and ^2^H is very similar, the spectra can be visually compared, and a large peak was apparent in the ^2^H NMR spectrum in the cases where deuterium was incorporated in the molecule (Figure 5c).

**Figure 6:**
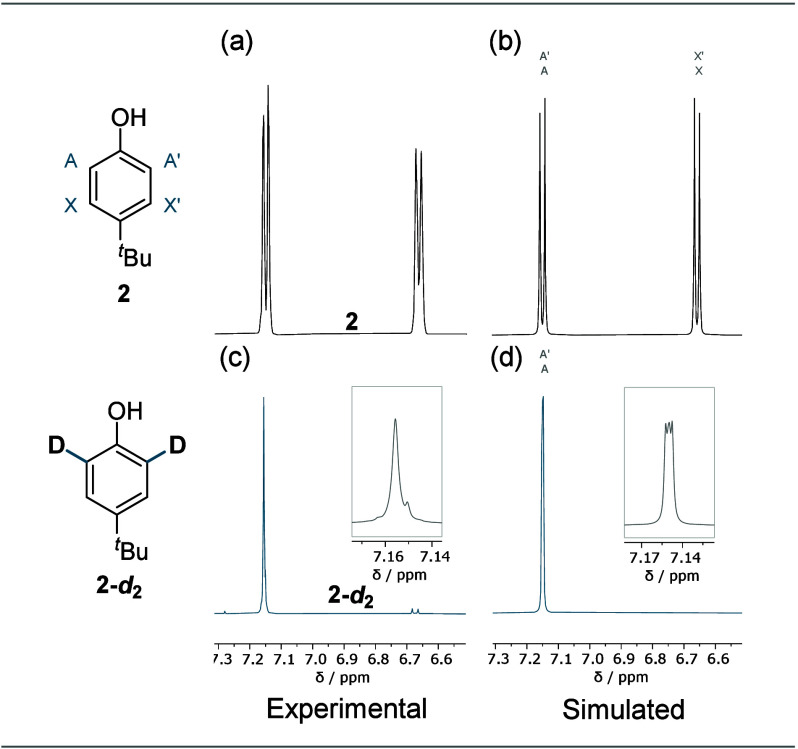
Comparison of the experimental and simulated ^1^H NMR spectra of (a, b) compound **2** and (c, d) **2-*****d***_**2**_.

To understand the influence of deuterium on the spectra in a hands-on way, students were tasked to first simulate the AA′XX′ spin system of compound **2**. The changes upon deuteration (**2-*****d***_**2**_) were also simulated and compared with the experimental spectra (Figure 6).

By taking into account the respective coupling constants observed for the nondeuterated compound **2**, the students gained insights into the composition of an AA′XX′ spin system, in which two protons are chemically but not magnetically equivalent. The students could decipher the underlying more complicated nature of the apparent experimental singlet of **2-*****d***_**2**_, which is due to small coupling constants, line broadening, and the higher order signal.

To analyze the influence of ^2^H incorporation on first-order spin systems, tri- and tetrasubstituted arenes were simulated based on typical chemical shifts and coupling constants provided to the students.

**Figure 7:**
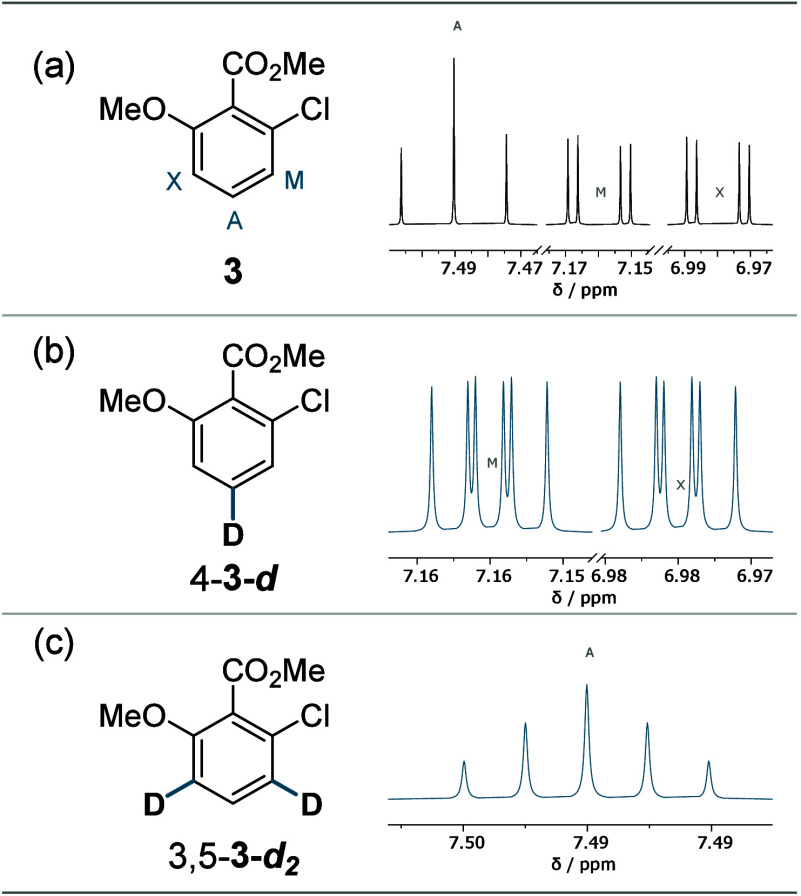
Example of a trisubstituted arene (a) in its native form, (b) monodeuterated, and (c) dideuterated with the respective simulated signals.

The number of signals is reduced upon deuteration of **3** (see Figure 7), but the signals show additional splitting. The initial three doublets of doublets turn into two doublets of triplets for 4-**3-*****d*** and into a triplet of triplets for 3,5-**3-*****d***_**2**_ (observed as an apparent quintet). The students were tasked to rationalize the altered signal intensities using Pascal-like triangles (here 1:2:3:2:1 vs classical quintet 1:4:6:4:1).^22^

To make further use of the simulation, the students were tasked to investigate the influence of acquisition time (number of points), line width, and differing spectrometer frequencies by systematically varying the simulation parameters (for detailed information, see the SI).

### Assessment of Students and Evaluation

The students were graded based on their laboratory reports and, to lesser extent, on their experimental performance. A set of additional questions and detailed answers are provided in the SI as a guideline for writing the report.

Key aspects to be covered in the report were general information about C–H activation and deuteration, mechanistic considerations, and the effects of deuterium labeling on NMR spectra (e.g., rationalization of the multiplicities in simulated spectra).

**Figure 8:**
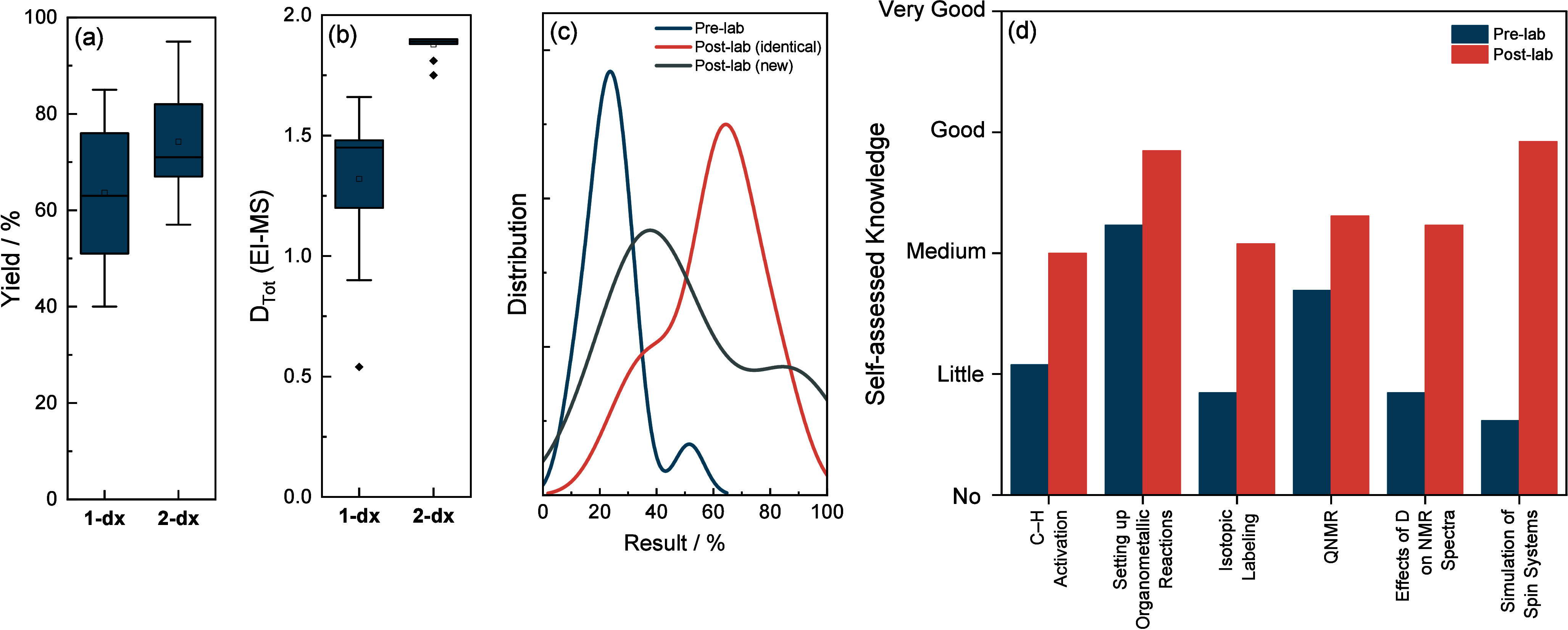
Box plots of (a) the crude yield and (b) the total deuteration degree (via EI-MS) obtained by the students for **1-*****d***_**X**_ and **2-*****d***_**X**_. Outliers can be explained by the malfunction of a stirring plate. Further reasons for the variations in (a) and (b) are discussed in the SI. (c) Distribution of the students’ results from a test performed before and two tests performed after the course with identical and new questions, respectively. (d) Self-assessment of the student’s knowledge corresponding to the learning outcomes prior to and after the course.

The lab course was carried out by 15 students in the first year of their full-time chemistry M.Sc. studies. This would make the course suitable for final-term undergraduate or first-year graduate students in the US system. The lab course was carried out on two consecutive half days, such that the reactions could run overnight (14–17 h). Potential modular variations to shorten or adapt the lab course to other departmental requirements are discussed in the SI. According to the students, an average of 7.2 h was required for the lab work, 3.5 h for analyzing the spectra, and 13.0 h for writing the report. All students were able to obtain deuterated compounds suitable for analysis (Figure 8a,b). Differences in yield and deuteration degrees and possible pitfalls were discussed in the students’ reports (and the SI).

The learning outcomes were assessed by means of self-evaluation of the students prior to and after the course and a pre- and post-lab test, the latter featuring identical as well as new questions. Students overall markedly improved their tested knowledge (Figure 8c, Table S2) and also felt subjectively more confident (Figure 8d) in their understanding of C–H activation, deuterium labeling, and qNMR.

## Conclusion

The lab course aims to equip students or non-C–H activation experts with the tools and knowledge to carry out palladium-catalyzed C–H activation reactions and with the means to quantify deuterium incorporation. An important qualitative goal is to fight the incorrect notion that C–H activation remains limited to research groups actively investigating such reactions and to demonstrate that such methods have become user-friendly and can be implemented in any synthetic organic chemistry lab.

The learning outcomes were quantifiably met, and an additional student feedback survey (Table S4) revealed overall satisfaction with the current course.

The lab work with a variation of a state-of-the-art deuteration method is complementary to other reported deuteration lab courses that focus more on kinetic aspects^23−27^ or use conventional synthetic methods like reduction^28^ or acid–base exchange.^29−31^ The detailed NMR simulations for isotopologues complement other more theoretical deuteration studies focusing, for example, on infrared spectra.^32,33^ This course furthermore adds an example of a nondirected arene C–H activation/functionalization to the few reported lab courses covering directed C–H activation^34,35^ or heteroaromatic substrates^36^ and hence constitutes a valuable new tool for teaching in modern synthetic organic chemistry lab courses.

## Data Availability

Further supporting information containing the Supporting Information document as a DOCX file; seminar slides (PPTX); student evaluation (XLSX); student instructions including a safety data sheet, tutorials, and report questions (PDF); MS data; and raw NMR data (Bruker, JCAMP-DX) is available on Zenodo at DOI: 10.5281/zenodo.11653341.

## Supporting Information Available

Equipment and chemical information, safety considerations, student instructions, instructor information, student performance and feedback, and possible deviations from the original experiment (PDF)
