# Supplementary material for: Deuteration of Arenes via Pd-Catalyzed C–H Activation: A Lesson in Nondirected C–H Activation, Isotopic Labeling, and NMR Characterization
Source: J Chem Educ. 2024 Jul 16;101(8):3410–7. doi: 10.1021/acs.jchemed.4c00270 (PMC11327962; doi:10.1021/acs.jchemed.4c00270)
Supplement: Supplementary file 1 — ed4c00270_si_001.pdf [file ed4c00270_si_001.pdf]

Supporting Information

Deuteration of Arenes via Pd-Catalyzed C–H Activation – A Lesson in Nondirected C–H Activation, Isotopic Labelling and NMR Characterization

Fritz Deufel[a], Manuel van Gemmeren[a],*

Otto-Diels-Institut für Organische Chemie, Christian-Albrechts-Universität zu Kiel, Otto-Hahn-Platz 4, 24118 Kiel, Germany, * vangemmeren@oc.uni-kiel.de

Contents

1 General Information 1
1.1 Equipment 1
1.2 Chemicals 1
2 Safety Considerations 2
2.1 Safety Datasheet (SDS) 2
3 Student Instructions 8
3.1 Experimental Procedure for the C–H Activation 8
3.2 Report Instructions 10
3.3 Calculate the Degree of Deuteration (via MS) 12
3.4 Calculate the Degree of Deuteration and Yield (via NMR) 14
3.5 Simulate NMR Coupling Pattern 14
3.6 Advice for Processing Experimental Spectra 15
4 Information for the Instructors 17
4.1 Additional Experimental Notes 17
4.2 Oral Questions prior to the Labwork 20
4.3 Possible Answers to Report Questions (Practical Part) 21
4.4 Possible Answers to Report Questions (Theoretical Part) 29
4.5 Additional Seminar 37
4.6 Useful Literature for Students and Instructors 37
5 Student Performance and Feedback 37
5.1 Graduate Student Course and Performance 37
5.2 Learning Outcomes 38
5.3 Student Feedback 40
5.4 Grading of Students 40
6 Possible Deviations from the Original Experiment 44
6.1 Use of Optimized Reaction Conditions and Different Substrates 44
6.2 Use of Different Reaction Vessels 44
6.3 Modularity of the Course 45
7 References 47

1 General Information

1.1 Equipment

Nuclear Magnetic Resonance Spectroscopy (NMR)

Proton (^{1}H) nuclear magnetic resonance spectra and carbon (^{13}C) nuclear magnetic resonance spectra were recorded on a Bruker DRX-500 spectrometer. Chemical shifts are given relative to tetramethylsilane (TMS) and are referenced to the residual proton signal of the NMR solvent.[1] Data are presented as follows: chemical shift, multiplicity (s=singlet, d=doublet, t=triplet, q=quartet, m=multiplet, and br=broad signal), coupling constant in Hertz (Hz) and integration. Full assignment of the signals was achieved by using 2D NMR techniques (^{1}H–^{13}C HSQC, ^{1}H–^{13}C HMBC, ^{1}H–^{1}H COSY-DQF) (raw data provided for students).

^{2}H NMR spectra were recorded in deuterated solvent, using a solvent that has a sufficiently large chemical shift difference with the region of interest. The zg2h pulse sequence was employed.

It is possible to use lower frequency instruments. We recommend a minimum of 300 MHz for compound **1** whereas compound **2** is even suitable for analysis with a 60 MHz benchtop NMR device. Raw data for starting materials and products are provided for comparability and to enable a completely NMR "experiment free" lab course.

Mass Spectrometry (MS)

GC-MS spectra were recorded on an Agilent Technologies 7890A GC-system with an Agilent 5975C VL MSD or an Agilent 5975 inert Mass Selective Detector (EI) and a HP-5MS column (30 m × 0.32 mm, film thickness: 0.25 µm). Please note, that an ESI or EI HRMS measurements work equally well for the determination of the overall degree of deuteration. It might be recommendable to retrieve a small sample prior to addition of internal standard in case no chromatographic separation (GC or LC) will be done.

The raw GC and EI-MS data for analysis is provided for comparability and to enable a MS "experiment free" lab course.

Materials

Stirrers: ICA RET or RCT basic stirrers with a connected thermometer in combination with a silicon oil bath
Balances: Sartorius Entris® II Essential Line Analytical Balance
Vials: Thermo Scientific™ 18mm Headspace Vials (10 mL) from Thermo Fisher with ROTILABO® ND18 screw caps (butyl red, PTFE grey) from CarlRoth
Gloves: Nitopren® 717 gloves (for aqua regia handling and disposal of chemicals), Nitrile disposable gloves (for weighing in, handling of nonhazardous starting materials).
Syringes: Injekt® syringes (1 mL, 3 mL) and Sterican® needles (0.8 x 120 mm) from B. BRAUN
Stirring bar: rare earth PTFE stirring bar, elliptic shape (10 x 6 mm), e.g. from Cowie® (for catalysis reaction); PTFE stirring bar cylindrical (10 x 5 mm) (or other, for stirring of stock solution)

1.2 Chemicals

All chemicals used herein are commercially available from common vendors. The vendor is explicitly indicated in case it is suspected that the quality could be crucial for a very precise reproducibility of the results.

1,1,1,3,3,3-Hexafluoroisopropanol (HFIP), CAS: 920-66-1, vendor: fluorochem
Palladiumacetate, CAS: 3375-31-3, vendor: SigmaAldrich
Deuterium oxide, CAS: 7789-20-0
N-Acetylglycine, CAS: 543-24-8
Methyl 6-methylnicotinate, CAS: 5470-70-2, Note: check purity with NMR/TLC, potentially purify via flash chromatography (EA/*n*-pentane)
7-methyl-2*H*-benzo[*b*][1,4]dioxepin-3(4*H*)-one (watermelon ketone/ Calone), CAS: 28940-11-6
4-(*tert*-butyl)phenol, CAS: 98-54-4
DMSO-d_6, CAS: 2206-27-1
Conc. HCl, CAS: 7647-01-0
Conc. HNO_3, CAS: 7697-37-2
Celite, CAS: 68855-54-9
Ethyl Acetate, CAS: 141-78-6
1,3,5-Trimethoxybenzene (internal standard), CAS: 621-23-8
Silica gel, CAS: 63231-67-4

2 Safety Considerations

Summarized main safety aspects:

Pressurized reaction vial (reaction above solvent boiling point and elevated temperatures)

Cleaning vials and stirring bars with aqua regia

- Use disposable vials
- OR: follow the safety instructions in SDS carefully

Needles

Chemicals

- HFIP
- 4-(*tert*-butyl)phenol
- Aqua regia (HCl and HNO_3)

2.1 Safety Datasheet (SDS)

Please note that chemical waste might be handled differently at your institute.

Saftey Data Sheet

for the laboratory experiment “Deuteration of Arenes”

Personal Information

Name: **First Name**: Building: Room Nr.:

Emergency telephone number (laboratory supervisor):

Reaction Overview

Reaction

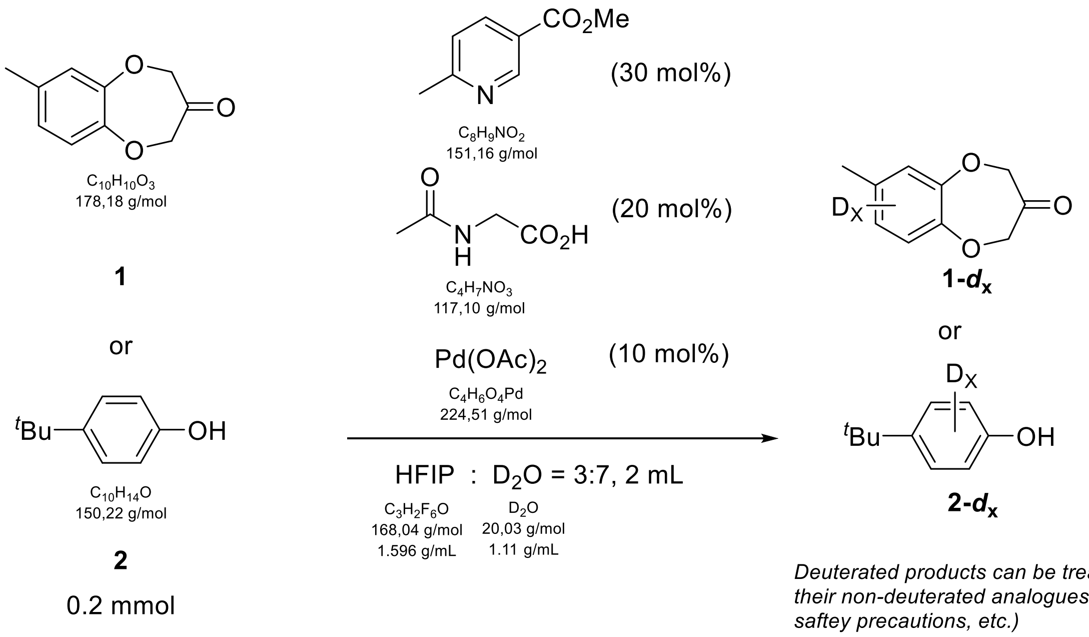

Deuterated products can be treated like their non-deuterated analogues (disposal, saftey precautions, etc.)

Work-up

GC-MS sample preparation:
Ethyl acetate, Silica gel

$C_4H_8O_2$
88,11 g/mol
0.902 g/mL

NMR sample preparation:
DMSO-d_6,CH_2Cl_2, Celite, 1,3,5-trimethoxybenzene

C_2D_6OS 84,17 g/mol 1.190 g/mL; CH_2Cl_2 84,93 g/mol 1.325 g/mL; $C_9H_{12}O_3$ 168.19 g/mol

Chemical Compounds (list)	Fp	Bp/ Mp	GHS Symbol, Signal word	Number of H- + P-statements	Cancerogenic Mutagenic Teratogenic	Required amount (g, mL, mmol)
7-methyl-2*H*-benzo[*b*][1,4]dioxepin-3(4*H*)-one		-/38°C	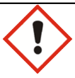 Warning	H302	-	35.6 mg, 0.2 mmol
1,1,1,3,3,3-hexafluoroisopropanol (HFIP)		59°C/-4°C	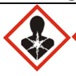 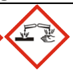 Danger	H314, H361, H373, P202, P260, P280, P303 + P361 + P353, P304 + P340 + P310, P305 + P351 + P338	T (cat 2)	0.6 mL
D_2O	-	101°C / 4°C	-	-	-	1.4 mL
4-(*tert*-butyl)phenol	113 °C	236°C/96° C	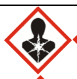 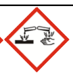 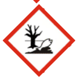 Danger	H315, H318, H361f, H410, P202, P273, P280, P302 + P352, P305 + P351 + P338, P308 + P313	T (cat 2)	30.0 mg, 0.2 mmol

methyl 6-methylnicotinate	103 °C	160°C(140 mbar)/34°C	Warning	H315, H319, H335, P261, P264, P271, P280, P302 + P352, P305 + P351 + P338	-	12.0 mg, 0.08 mmol
2-acetamidoacetic acid	-	>300°C/207°C	-	-	-	15.0 mg, 0.12 mmol
$Pd(OAc)_2$	-	-/216°C	Danger	H317, H318, H410, P261, P272, P280, P302 + P352, P305 + P351 + P338		9.0 mg, 0.04 mmol
Hydrochloric acid (conc.)		>100°C/-30°C	Danger	H290, H314, H335, P234, P261, P271, P280, P303+P361+P353, P305+P351 + P338	-	≈ 9 mL
Nitric acid (conc.)		120°C/	Danger	H272, H290, H314, H331, P210, P220, P280, P303+P361+P353, P304+P340+P310, P305+P351+P338, EUH071		≈ 3 mL
DMSO-d_6	88°C	189°C/20°C	-	-	-	≈ 0.6 mL
1,3,5-trimethoxybenzene	86°C	255°C/54.5°C/	Warning	H302, P264, P270, P301 + P312, P501	-	≈ 16 mg
Celite	-	>450°C/-	Danger	H372, P260,P260,P270,P314,P501	-	≈ 10 g
Ethyl acetate	-4°C	77°C/-83°C	Danger	H225, H319, H336, P210, P233, P240, P241, P242, P305+P351+P338, EUH066	-	≈ 50 mL
Silica gel	-	>450°C/-	-	-	-	≈ 500 mg

Relevant Hazard Statements and Precautionary Statements

H225	Highly flammable liquid and vapor.
H272	May intensify fire; oxidizer.
H290	May be corrosive to metals.
H302	Harmful if swallowed.
H314	Causes severe skin burns and eye damage.
H315	Causes skin irritation.
H317	May cause an allergic skin reaction.
H318	Causes serious eye damage.
H319	Causes serious eye irritation.
H331	Toxic if inhaled.
H335	May cause respiratory irritation.
H336	May cause drowsiness or dizziness.
H351	Suspected of causing cancer.
H361	Suspected of damaging fertility or the unborn child.
H361f	Suspected of damaging fertility.
H372	Causes damage to organs (Lungs) through prolonged or repeated exposure if inhaled.
H373	May cause damage to organs through prolonged or repeated exposure.
H410	Very toxic to aquatic life with long lasting effects.
P202	Do not handle until all safety precautions have been read and understood.
P210	Keep away from heat, hot surfaces, sparks, open flames and other ignition sources. No smoking.
P220	Keep away from clothing and other combustible materials.
P233	Keep container tightly closed.
P234	Keep only in original packaging.
P240	Ground and bond container and receiving equipment.

P241	Use explosion-proof electrical/ ventilating/ lighting/ equipment.
P242	Use nonsparking tools.
P260	Do not breathe mist or vapors.
P261	Avoid breathing dust.
P264	Wash skin thoroughly after handling.
P270	Do not eat, drink or smoke when using this product.
P271	Use only outdoors or in a well-ventilated area.
P272	Contaminated work clothing should not be allowed out of the workplace.
P273	Avoid release to the environment.
P280	Wear protective gloves/ protective clothing/ eye protection/ face protection.
P301 + P312	IF SWALLOWED: Call a POISON CENTER/ doctor if you feel unwell.
P301 + P312 + P330	IF SWALLOWED: Call a POISON CENTER/ doctor if you feel unwell. Rinse mouth.
P302 + P352	IF ON SKIN: Wash with plenty of water.
P303 + P361 + P353	IF ON SKIN (or hair): Take off immediately all contaminated clothing. Rinse skin with water.
P304 + P340 + P310	IF INHALED: Remove person to fresh air and keep comfortable for breathing. Immediately call a POISON CENTER/ doctor.
P305 + P351 + P338	IF IN EYES: Rinse cautiously with water for several minutes. Remove contact lenses, if present and easy to do. Continue rinsing.
P308 + P313	IF exposed or concerned: Get medical advice/ attention.
P314	Get medical advice/ attention if you feel unwell.
P501	Dispose of contents/ container to an approved waste disposal plant.
EUH066	Repeated exposure may cause skin dryness or cracking.
EUH071	Corrosive to the respiratory tract.

Additional Information Regarding Danger for Humans/Environment for Starting Materials/Products (e.g. TLV, LD_{50}, WHC, etc.):

TLV = threshold limit value, WHC = water hazard class = derived from the German WGK (Wassergefährdungsklasse) classification system (0 = not hazardous, 1 = slightly hazardous, 2 hazardous, 3 extremely hazardous to water)

7-methyl-2*H*-benzo[*b*][1,4]dioxepin-3(4*H*)-one: WHC2
1,1,1,3,3,3-hexafluoroisopropanol: WHC2
D_2O: -
4-(*tert*-butyl)phenol: LD50 Oral - rat - > 2.000 mg/kg, WHC3
methyl 6-methylnicotinate: LD50 Oral – rat - 2.500 mg/kg, WHC3
2-acetamidoacetic acid: -
$Pd(OAc)_2$: LD50 Oral - Rat - >= 5.110 mg/kg, WHC2
Hydrochloric acid (conc.): WHC1
Nitric acid (conc.): WHC1
DMSO-d_6: LD50 Oral - 28.300 mg/kg, LC0 Inhalation - Rat - male and female - 4 h - > 5,33 mg/l - dust/mist, LD50 Dermal - Rat - male and female - 40.000 mg/kg, WHC1
1,3,5-trimethoxybenzene: LD50 Oral - Mouse - 1.480 mg/kg, WHC3
Celite: LD50 Oral - Rat - female - > 2.000 mg/kg, LC50 Inhalation - Rat - male and female - 4 h - > 2,6 mg/l - dust/mist, WHC1
Silica gel: -
Ethyl acetate: LD50 Oral - Rat - 5.620 mg/kg, LD50 Dermal - Rabbit - male - > 20.000 mg/kg, WHC1

Precautionary Protective Measures and Rules of Conduct:

General:
Wear lab coat, protective goggles, and safety gloves (nitrile during experiment, acid resistant thick gloves for cleaning)
No food, drinks in the laboratory, no smoking on the laboratory premises
Do not inhale chemical vapors, perform the experiment in a well-ventilated fume hood
Avoid skin and eye contact, clean your hands thoroughly after leaving the lab environment
Remove ignition sources
Keep your lab space clean, organized and easy to access in case of an emergency
Label your flasks and experiments (in case it is run over night)
Inform yourself about the adequate disposal of the used chemicals
Do not contaminate the environment and water system with chemicals

Experiment specific:
Aqua regia (1/3 mixture v/v conc. nitric acid/ conc hydrochloric acid):
Wear thick acid resistant gloves and perform the cleaning in the fume hood with the protective sash window lowered as much as possible assure good ventilation -> brown gaseous nitrogen oxides evolve (toxic!),
Remove glassware only from fume hood after gases have evaporated and they have been rinsed with water and e.g. acetone,
Carefully dilute residual aqua regia with water (add ice in a water bath and carefully add aqua regia),
Adjust the pH depending on your departmental regulations and deposit as aqueous waste
Celite, Silica gel:
Do not inhale dust, work in fume hood

Measures in case of Danger, First-Aid Measures:

General:
Inform teaching staff, lab supervisor
Remove contaminated clothes, ensure supply of fresh air in case of inhalation, contact doctor in case of discomfort,
In case of breathing arrest, use CPR and call emergency physician
Contact with skin: wash thoroughly first with water, then with water and soup, contact doctor in case of continued discomfort
Contact with eye: open eyelid, rinse the eye several minutes (>10 min) with running water (use eye shower), contact doctor in case of continued discomfort
Swallowing: Rinse mouth with water, do NOT induce vomiting, contact instantly doctor and/or the poison control center
Fire: use CO_2 or class ABC-fire extinguisher, alert firefighters in case of a larger/uncontrolled fire, evacuate building
Exposition to the environment: remove ignition sources, alert the immediate surrounding, evacuate the premises, dispose the substance according to regulations, ventilate the room

Follow the indication of the specific H- and P-statements

Hazards Due to Experimental Set-up:

Pressurized reaction vessel:
The reaction is heated above the boiling point of one of the solvents, ensure an intact seal and a crack-free glass vessel
Do NOT puncture the seal with a needle!
The reaction mixture is nonflammable and does not require water-cooling so the reaction can be run in a fume-hood overnight (with safe electrical devices)
Allow the reaction to cool to room temperature before opening the reaction vial
Let the apparatus cool down to room temperature before removing the oil bath/the heating block
Needles:
Needles with (or without) chemicals can puncture the skin. Use with care. Quench content in syringe/needle if required
Leave no needle lying around openly/unprotected. Use rubber septa/stopper to secure the pointy end.
Dispose needle in special, puncture resistant safety container.
Removing Pd traces with aqua regia (see section above)

Disposal of (Chemical) Waste:

Organic nonhalogenated solvent waste:
7-methyl-2*H*-benzo[*b*][1,4]dioxepin-3(4*H*)-one, 4-(*tert*-butyl)phenol, methyl 6-methylnicotinate, DMSO-d_6, 1,3,5-trimethoxybenzene, ethyl acetate, products

Organic halogenated solvent waste:
1,1,1,3,3,3-hexafluoroisopropanol

Acidic aqueous waste:
D_2O, HCl (conc.) -> after dilution with water, HNO_3 (conc.) -> after dilution with water

Solid chemical waste/contaminated products:
2-acetamidoacetic acid, $Pd(OAc)_2$, Celite, Silica gel, contaminated & broken glassware, contaminated one-way gloves/paper towels/syringes/pipettes

Needle container:
Needles

I hereby pledge to undertake the laboratory experiment according the safety instructions and will undertake all the specified precautionary safety instructions stated herein<br><br>Date: ________________________<br>Signature of the student	I verified that the student read the safety data sheet, is aware of the required safety measures and the proper disposal of chemical waste and allow the student to carry out the experiment<br><br>________________<br>Signature of the laboratory instructor

Fp = Flashpoint, Bp = Boiling point, Mp = Melting point, TLV = Threshold limit value,
WHC = Water hazard class

3 Student Instructions

3.1 Experimental Procedure for the C–H Activation

3.1.1 Laboratory Instructions

Day 1

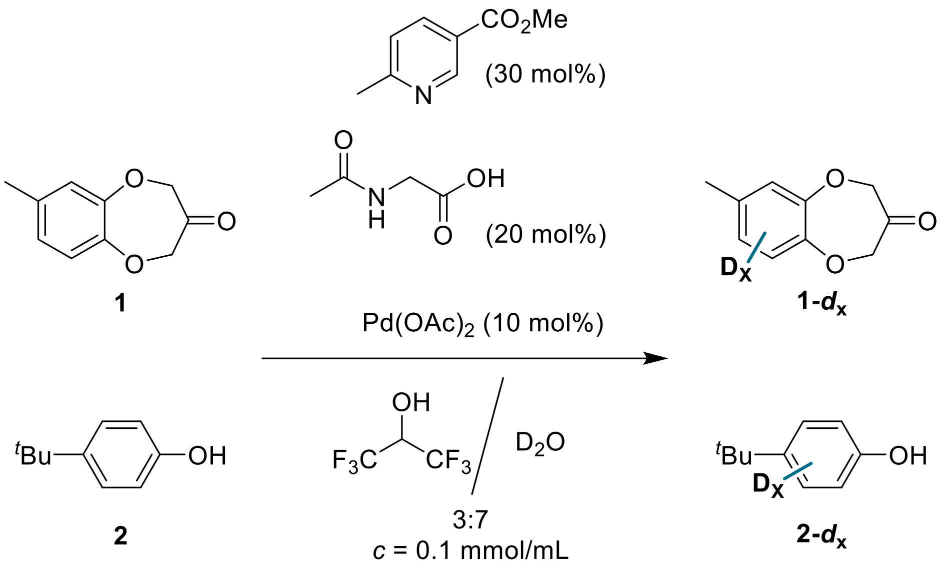

Figure S 1: Overview of the deuteration reactions.

Preparation of Catalyst Stock Solution (for *x* Reactions)

An oven dried vial was charged with Pd(OAc)2 (4.5 mg · ***x***, 20 µmol · ***x***, 10 mol%), *N*-acetylglycine (7.0 mg · ***x***, 60 µmol · ***x***, 30 mol%) and methyl 6-methylnicotinate (3.0 mg · ***x***, 40 µmol · ***x***, 20 mol%) and a magnetic stirring bar. HFIP (0.60 mL · ***x***) was added, the vial was tightly sealed and the reaction was stirred at room temperature for at least 10 minutes.

Advice: The stock solution was prepared for at least one additional reaction ($x_{stocksol} = x_{reactions} + 1$) since the last solvent drops can be difficult to be taken up by a syringe.

Preparation of Internal Standard Stock Solution (Example)

1,3,5-trimethoxybenzene (500 mg, 2.97 mmol) was added in a volumetric flask and EtOAc was added (25.0 mL). The flask was tightly sealed, wrapped with parafilm and shaken vigorously. The solution (0.119 M) was stored in a fridge until needed and was preferably prepared freshly the same day.[I]

Compound 1-d_X

An oven dried 10 mL pressure resistant vial was charged with 7-methyl-2*H*-benzo[*b*][1,4]dioxepin-3(4*H*)-one (35.6 mg, 200 µmol) and a rare earth stirring bar. The catalyst stock solution (0.600 mL) and D_2O (1.40 mL) were quickly added and vial was tightly sealed. The vial was then placed in a preheated silicon oil bath on top of a magnetic stirrer and stirred at 80°C for 18h.

Compound 2-d_X

An oven dried 10 mL pressure resistant vial was charged with 4-(*tert*-butyl)phenol (30.0 mg, 200 µmol) and rare earth stirring bar. Rapidly add the catalyst stock solution (600 µL), D_2O (1.40 mL) and tightly seal the pressure resistant vial. The vial was then placed in a preheated silicon oil bath on top of a magnetic stirrer and stirred at 80°C for 18h.

I Note: the precise concentration is not crucial and can be adapted depending on the number of reactions, as long as the final amount of substance that was added to one reaction mixture is reported and accounted for in the yield calculation (acceptable range n(IS) ≈ 0.04 – 0.3 mmol).

Day 2

The reaction mixture **1** or **2** was allowed to cool to room temperature. The vial was opened and the stock solution of 1,3,5-trimethoxybenzene (500 µL, 0.119 M, 60 µmol) was added as an internal standard.[II]

Workup:

The reaction mixture was diluted with EtOAc (≈ 2 mL), the vial was sealed and vigorously shaken. After reopening the vial, an aliquot (≈ 0.1 mL) of the organic layer was filtered over a silica filled pipette using EtOAc and collected in a GC-vial. The remaining organic layer of the reaction mixture was filtered over a pad of Celite®. EtOAc (≈ 2 mL) was added to the remaining aqueous layer, shaken, and the organic layer was taken up again with a pipette and filtered over the Celite® pad. This was repeated at least three times and the pad was washed with EtOAc (≈ 20 mL). The overall solution was concentrated under reduced pressure, DMSO-d_6 (0.7 mL) was added and the resulting solution was transferred to an NMR tube.

Cleaning:

Palladium contaminated single use reaction vials and caps/seals should be disposed in the solid waste after the experiment. Pd contaminated stirring bars are collected in a vial and stirred in an aqua regia solution (1:3 conc. HNO_3:conc. HCl) at 50°C for 1h. Ensure that the vial placed in a well ventilated fumehood (NO_x gases!) and is covered to avoid splashing. Rinse the vial and stirring bars with water and acetone in your fumehood. *Note: Be careful and follow the SDS strictly regarding aqua regia handling! Ask the instructor to assist you with the cleaning in case you are not comfortable. Multi-use reaction vials (Schlenk tubes) should be cleaned and not disposed. Ask your instructor whether your glassware requires cleaning in case you are unsure.*

Analysis:

Compound 1-*d*X

NMR: ^{1}H (d1 = 30s), ^{13}C{^{1}H}, optional: ^{2}H

GC-MS

Compound 2-d_X

NMR: ^{1}H (d1=30s), ^{2}H

GC-MS

3.1.2 Additionally Provided Data

Ask your instructor for the NMR data of the starting materials (in Bruker and JDX Format).

Compound **1** in CD_2Cl_2: ^{1}H (d1 = 0.5s), ^{1}H (d1 = 30s), ^{13}C{^{1}H}, ^{1}H-^{1}H-COSY, ^{1}H-^{13}C-ed.HSQC, ^{1}H-^{13}C-HMBC
in DMSO-d_6: ^{1}H (d1 = 30s), ^{13}C{^{1}H}.

Compound **1-d_x** in DMSO-d_6: ^{1}H, ^{2}H, ^{13}C{^{1}H}

Compound **2** in DMSO-d_6: ^{1}H (d1 = 0.01s), ^{1}H (d1 = 1s), ^{1}H (d1 = 30s), ^{13}C{^{1}H}

II Note: Alternatively, instead of a stock solution, 1,3,5-trimethoxybenzene (e.g. 10.1 mg, 60.0 µmol) can be added directly to the reaction mixture as an internal standard.

3.2 Report Instructions

Write a report answering the following aspects in a **short** and **concise** way.

3.2.1 Experimental Evaluation

Compound 1

- Assign all NMR active atoms in the starting material of compound **1**. Indicate the correlations you used for the assignment (*optional*).[III]
- Determine the crude yield and degree of deuteration (specify both calculations) of compound **1-*d*ₓ** using ^{1}H-NMR.
- Determine the deuterium incorporation in each aromatic position (%^{2}H) (specify calculation).
- Determine the overall degree of deuteration using EI-MS with the LabelChecker module of the Universal Mass Calculator Software.[2]
- Stack and compare the ^{13}C-NMR of starting material and product only for the methyl group and the adjacent carbon (Figure S 2a). What do you observe?
- How did the signals ^{1}H-NMR, ^{2}H-NMR and ^{13}C-NMR signals change from the nondeuterated to the deuterated compound?

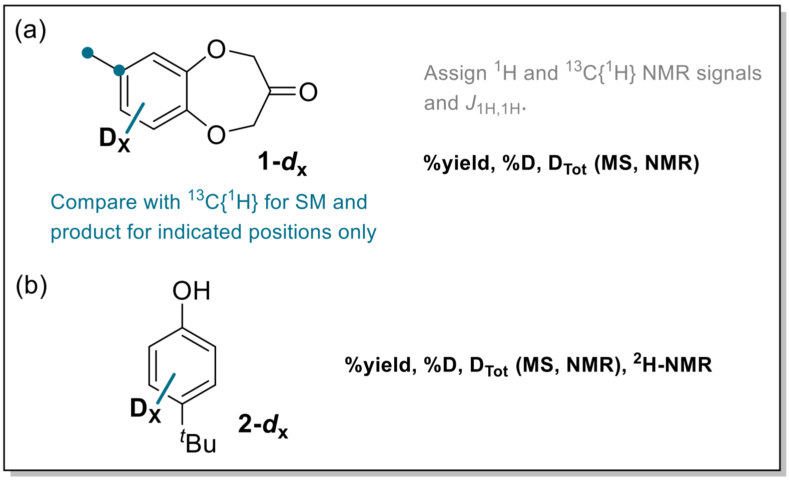

Figure S 2: Analytical tasks for(a) compound **1-*d*ₓ** and (b) **2-*d*ₓ**.

Compound 2

- Determine the crude yield and degree of deuteration of compound **2-*d*ₓ** using ^{1}H-NMR (Figure S 2b).
- Determine the deuterium incorporation in each aromatic position (%^{2}H) using ^{1}H-NMR and compare with ^{2}H-NMR (no integration required for ^{2}H-NMR).
- What spin system and what set of signals do you expect for compound **2**?
- How does the signal theoretically change from the native to deuterated compound?

NMR Simulation

- Simulate compound **2** and **2-*d*ₓ** using MestreNova with the experimental chemical shifts (Figure S 3a). Estimate the coupling constants of **2** from the simulation applying the relationship $J_{H,H} \approx 6.5 \cdot J_{H,D}$ for **2-*d*ₓ** [IV]
- Simulate the aromatic spin system (^{1}H NMR) for all isotopologues shown in Figure S 3b using the chemical shifts and coupling constants provided in Figure S 3. Change the simulation parameters for the indicated isotopologue (Figure S 3b) and compare with the standard settings. In all cases set the deuterium chemical shift to –3 ppm for simulation purposes.

[III] Note: The 2D spectra were recorded in CD_2Cl_2 since in DMSO-d_6 small quantities of ketone get converted to the hydrate. The order or ^{13}C and ^{1}H signals remain identical irrespective of the solvent. A comparison with the deuterated compound should be done in DMSO-d_6 for which spectral data is also provided.

[IV] Hint: If signals are chemically equivalent but magnetically inequivalent use not N = 2 but two distinct protons with the same chemical shift but a different coupling relation for your simulation. Assume perdeuteration in *ortho* to the OH group and no deuteration in the remaining molecule.

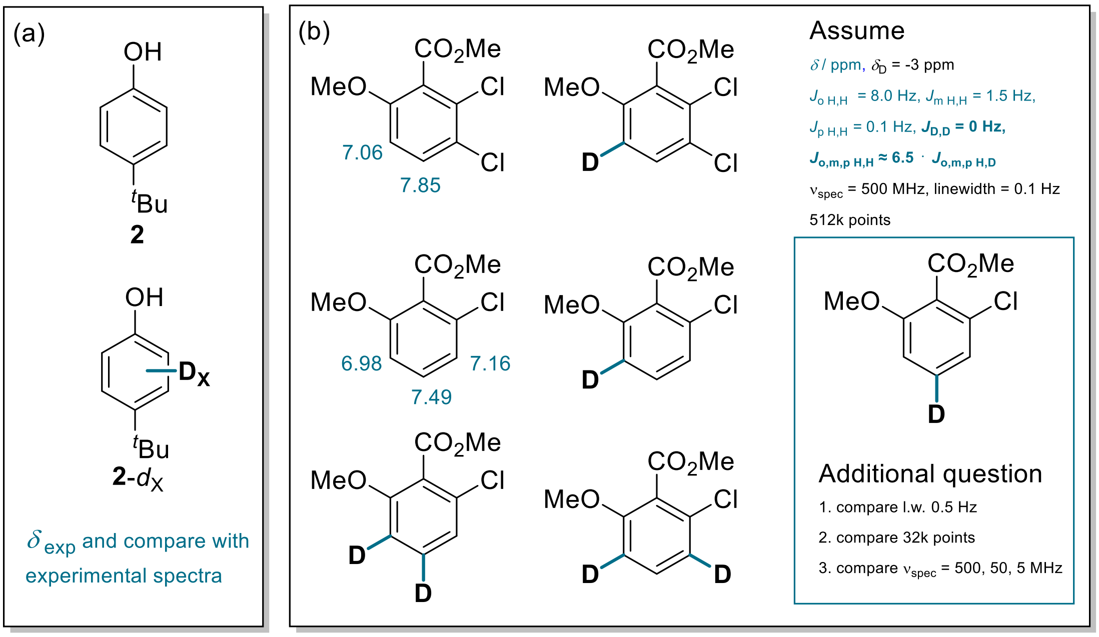

Figure S 3: (a) Isotopologues of compound **2** and **2-d_x** and (b) several model compounds.

3.2.2 Theoretical Aspects

Isotope Labelling

- Why/in which context do you think isotopic labelling could be relevant? Give examples especially for ^{2}H (and other nuclei). What is compound **1** used for? Can you think of a hypothetical application where a deuterated version of compound **1** is required?
- What other techniques apart from MS and NMR can be used to verify deuterium or tritium incorporation? Explain!

C–H Activation

- Give a definition of C–H activation and C–H functionalization.
- Give a mechanistic proposal for the catalytic cycle. What is the role of AcGlyOH and the Methyl 6-methylnicotinate?[V]
- Discuss for compounds **1** and **2**: why there are differing degrees of deuteration depending on the position?
- What is the difference between ambiphilic metal-ligand activation (AMLA) or concerted metalation deprotonation (CMD), and base-assisted intramolecular electrophilic-type substitution (BIES) or electrophilic concerted metalation deprotonation (eCMD)?[VI] What is the difference to e.g. an S_EAr reaction or a deprotonation?
- How could you achieve *selective* mono-deuteration of an arene using traditional non-C–H activation chemistry? (*optional*)
- What is late-stage functionalization (LSF) and why can C–H functionalization be beneficial in general? (*optional*)

qNMR and NMR

- Why is measuring ^{2}H-NMR usually done in nondeuterated solvents? How can you reference your shifts if you do not have residual solvent signals (e.g. no ^{2}H peak of the solvent in the ^{2}H NMR) (optional)
- Explain the multiplicity of the solvent (deuterated DMSO) in the ^{1}H-NMR and ^{13}C-NMR? A typical $^2J_{H,H}$ coupling constant is ~12 Hz, what are the coupling constants here? Why is $J_{H,H} \approx 6.5 \cdot J_{H,D}$?
- Why is a long relaxation delay used for quantitative NMR? Compare and stack the provided spectra of the starting material **1** (d1=0.5s, d1=30s)!

V Hint: Wedi, P.; Farizyan, M.; Bergander, K.; Mück-Lichtenfeld, C.; van Gemmeren, M. Mechanism of the Arene-Limited Nondirected C–H Activation of Arenes with Palladium. Angew. Chem. Int. Ed. 2021, 60 (28), 15641–15649. DOI: 10.1002/anie.202105092.

VI Hint: Carrow, B. P.; Sampson, J.; Wang, L. Base-Assisted C-H Bond Cleavage in Cross-Coupling: Recent Insights into Mechanism, Speciation, and Cooperativity. Isr. J. Chem. 2020, 60 (3-4), 230–258. DOI: 10.1002/ijch.201900095

- What constitutes a suitable internal standard for qNMR?
- Why is a normal ^{13}C-NMR experiment not suitable for qNMR? What do you need to record a qNMR experiment? (*optional*)
- Why is there no ^{13}C-^{1}H coupling visible in a normal ^{13}C-NMR experiment? (*optional*)

3.3 Calculate the Degree of Deuteration (via MS)

For publications please indicate:

The computer software UMC (Universal Mass Calculator) was used to [simulate isotope patterns, evaluate the degree of deuteration/labelling, evaluate elemental compositions]. UMC V3.11.0.27, Dr. Matthias C. Letzel, WWU Münster, Org.-Chem. Institut, Germany. https://www.uni-muenster.de/Chemie.oc/ms/downloads.html

Mass spectrometric determination of the overall deuteration incorporation:

1. (Install and) open the UMC Student Application, open the *LabelChecker* subprogramm

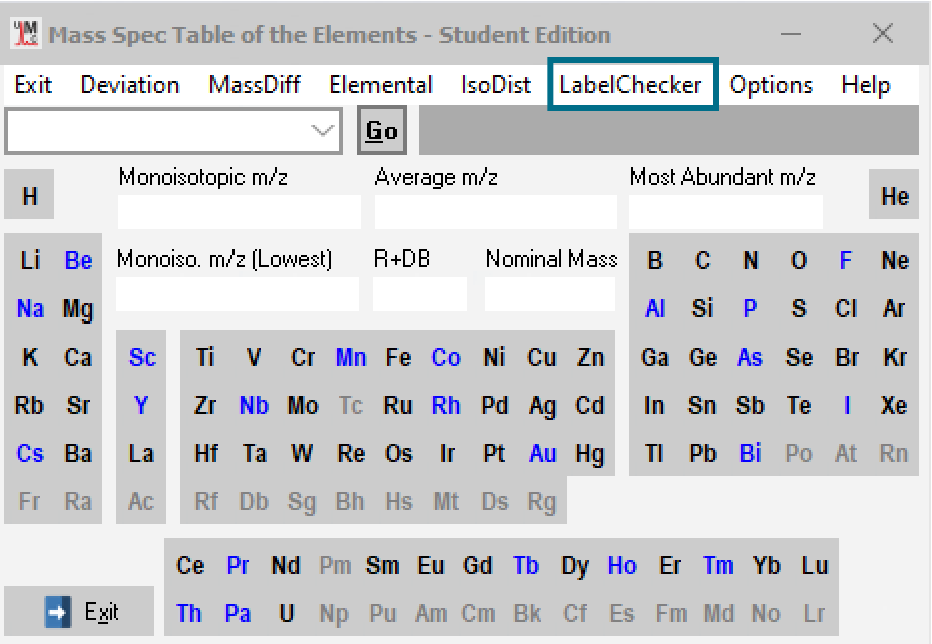

2. Add the chemical formula of your target molecule (! + 1xH in case e.g. ESI is used)
3. Click on *IsotopeList -> New*

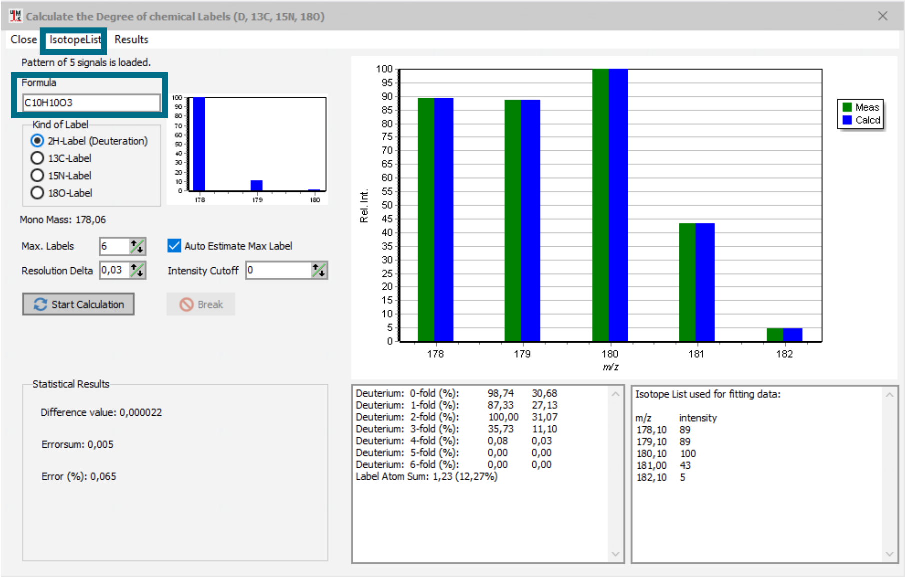

4. Manually add your Mass peaks and the respective Intensities. A new line can be added by pressing “Tab” on your keyboard

5. Start with the MS peak for your undeuterated substrate and add further peaks (until baseline noise/sensitivity of instrument)
6. Press *Close*

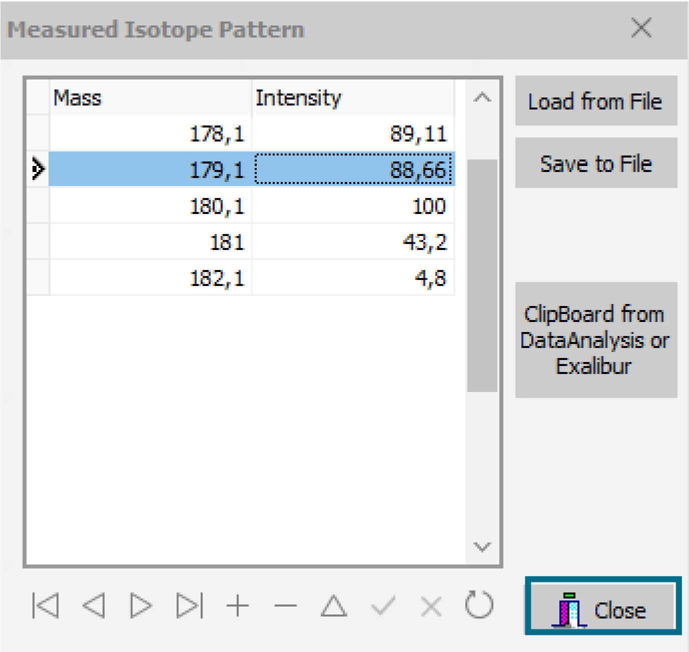

7. Make sure enough *Max. Labels* is indicated (this can influence the quality of the fit)
8. Play around with Resolution Delta (with our instrument 0.03 is a good value)
9. Press *Start Calculation*, check the that the fitting converged and note down the *Label Atom Sum* as the total deuterium content per molecule

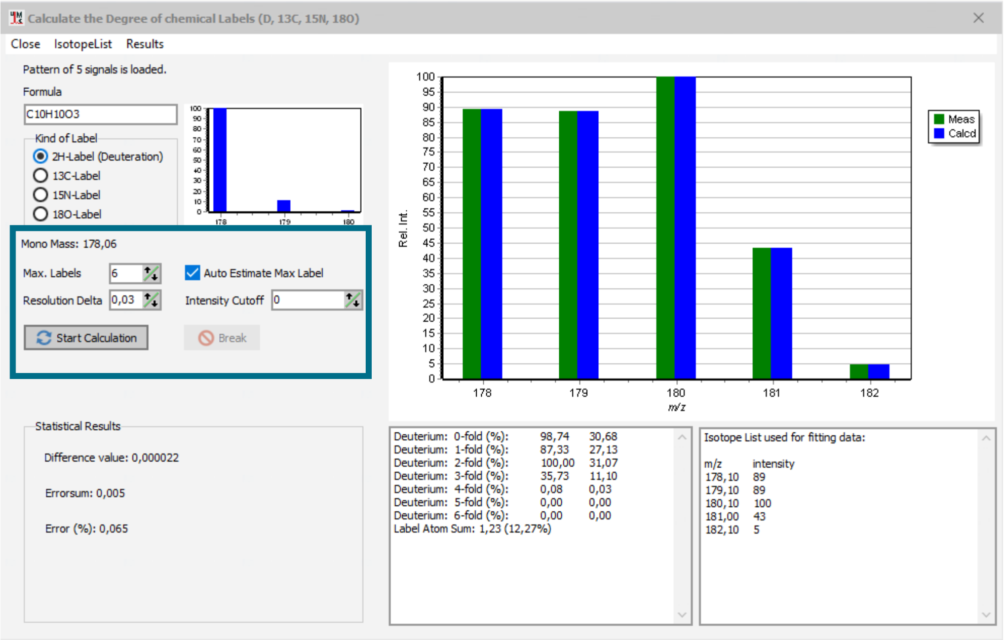

3.4 Calculate the Degree of Deuteration and Yield (via NMR)

For NMR analysis the decrease of the proton signal intensity was measured in ^{1}H NMR and compared with the values obtained from nondeuterated starting materials. The decrease in signal intensities was determined relative to positions in which no proton-deuterium exchange was observed. For comparison and determination of the chemical shift, the ^{1}H spectra of the nondeuterated compounds were recorded. Additionally, the yield of deuterated material was determined using 1,3,5-trimethoxybenzene as internal standard. A spectrum with sufficient relaxation delay (d1=30s) is required. Process the spectra, by referencing them (to TMS or the residual solvent signal) and by applying zerofilling /(or linear prediction), apodization (line broadening at least 0.2 Hz), phasing, and baseline correction.

To determine the degree of deuteration for the individual aromatic positions (%D) and the total degree of deuteration (D_{Tot}) equation 1 and 2 can be used. Hereby one needs to compare in the spectra of **1-*d*$_x$** or **2-*d*$_x$** the integration of a nondeuterated internal "reference", for instance a methyl group, and compare it with the integration of a position of interest in the same molecule i.e. the aromatic signals that have decreased in intensity upon deuteration. The crude yield can be thought of as the remaining, nondecomposed (deuterated) compound and can be calculated by integrating a nondeuterated position in the molecule of interest and the internal standard making use of equation 3.[VII]

$$\mathrm{D\%} = 1 - \frac{\mathrm{N_{eq.nuclei}(non{-}deut\ internal\ reference)}}{\mathrm{Int(non{-}deut\ internal\ reference)}} \cdot \frac{\mathrm{Int(position\ of\ interest)}}{\mathrm{N_{eq.nuclei}(position\ of\ interest)}} \quad (1)$$

$$\mathrm{D_{Tot}(NMR)} = \textstyle\sum_i \mathrm{D_i\%} \cdot \mathrm{N_{eq.nuclei}(i)} \quad (2)$$

$$\mathrm{yield\%} = \frac{n(\mathrm{IS})}{n(\mathrm{rxn\ scale})} \cdot \frac{\mathrm{Int(Product)}}{\mathrm{Int(IS)}} \cdot \frac{\mathrm{N_{eq.nuclei}(IS)}}{\mathrm{N_{eq.nuclei}(Product)}} \quad (3)$$

3.5 Simulate NMR Coupling Pattern

Since MestreNova® v.14.1 (also referred to as MNova®) is frequently used at our institute, the following, more detailed description is given for MestreNova.[3] It is alternatively possible to use Topspin[4] or WinDNMR[5] (both free for academic users) to simulate the spectra, which are both accessible free of charge for students. For Topspin, load the respective spectra (or a blank spectrum for the simulation of hypothetical compounds). Use the command "dnmr" and use the option "Add Nucleus" to define the spin system. Please be referred to the respective user manuals or publication for more details.

Simulation of coupling pattern in MestreNova:
Open the "Spin Simulation" Module in MNova

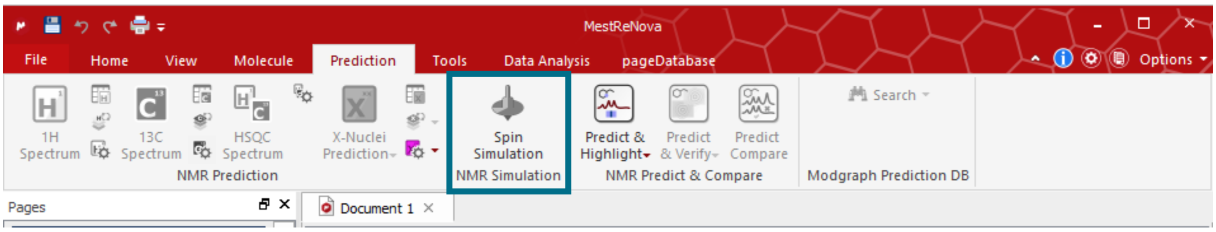

Adjust the data accordingly (label, chemical shifts, number of equivalent nuclei, spin, line width, spectrometer frequency) and press on "New Simulation"

[VII] Note: The reference spectra of the nondeuterated starting materials **1** and **2** are not needed for the determination of %D, D_{Tot} and %yield and are useful needed to determine the chemical shift of the deuterated signals in case high degrees of deuteration in **1-*d*$_x$** or **2-*d*$_x$** make the distinction between residual proton signal or impurities of ligands, etc. difficult. Stacking the spectra is therefore recommended.

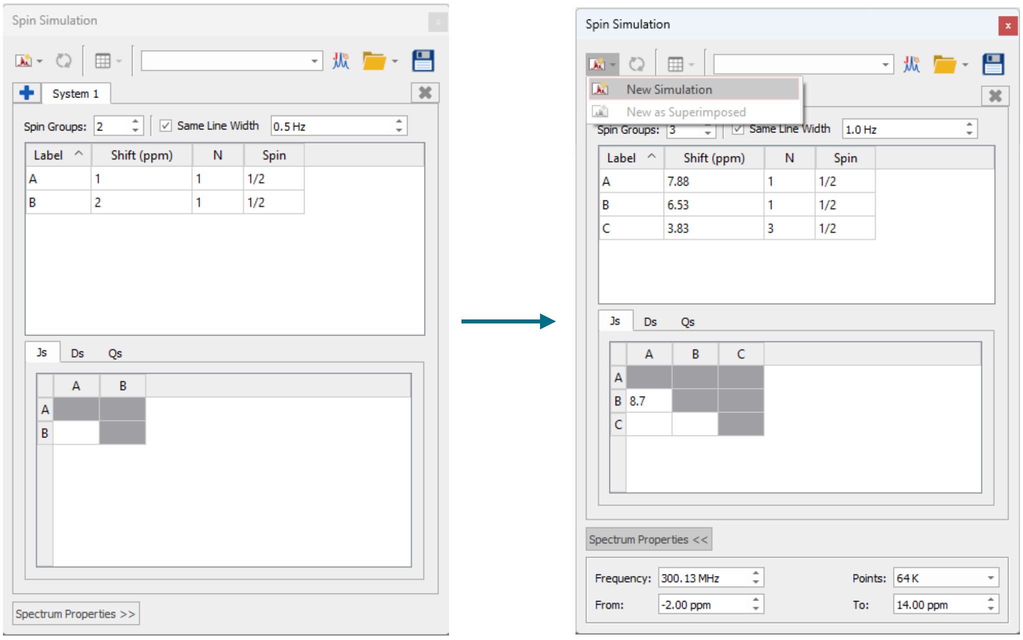

The spectrum you obtain can be processed like an experimental spectrum:

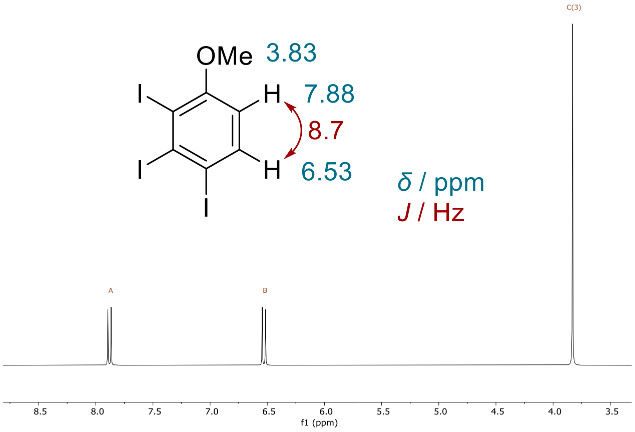

3.6 Advice for Processing Experimental Spectra

General recommendations (deviate from and experiment with the parameters to find the best-looking spectrum)

Processing 1D spectra
1. Window function
General: Exponential (0.2 Hz)
For good integration: Exponential (>0.5 Hz)
For good multiplicity: Exponential (0.1 Hz), Sine Square (e.g. 5°)
^{13}C, ^{2}H, ^{31}P: Exponential (>1 Hz)
2. Phase correction
3. Baseline correction (e.g. Polynomial 3rd order)
4. Referencing to TMS or residual solvent peak
5. Integrate, Pick peaks, Assign multiplets

Processing 2D magnitude spectra (e.g. COSY, HMBC)
1. Window function
Magnitude COSY or HMBC: SineBell 0 or SineSquare 0
2. baseline correction
(f2 and f2 -> try 2nd and 3rd order polynomial)
3. Zero filling or linear prediction
4. Referencing indirectly via 1H or via strong crosspeak (check proper shift in 1H before)

Processing 2D phase sensitive spectra (e.g. NOESY, HSQC,PE-COSY)
1. Window function
SineBell 90 or SineSquare 90
2. Phasing (manual phasing of f1 and f2 often better)
3. baseline correction
(f2 and f2 -> try 2nd and 3rd order polynomial)
4. Zero filling or linear prediction
5. Referencing indirectly via 1H or via strong crosspeak (check proper shift in 1H before)

For window function (also referred to as weighting function): display FID and evaluate other functions with a similar shape that can help you improve S/N.

Additional MNova specific advice (equal functions can be found e.g. in Topspin): MNova spectra can be pasted in a WORD document using Ctrl+C and Ctrl+V. You can report your picked peaks in publication format (e.g. for ^{13}C) using Tools -> Report -> Peaks (for ^{13}C 1 decimal) or multiplets (e.g. ^{1}H) using Tools -> Report -> Multiplets.
A molecule can be drawn or pasted from ChemDraw. You can assign crosspeaks from 2D spectra or multiplets from 1D spectra to the precise position in the molecule. This helps you keeping an overview for complex molecules and you can report the correlations/assignment.
Check literature or the MestreNova (https://mestrelab.com/downloads/mnova/manuals/MestReNova-14.2-0_Manual.pdf)/Topspin manual for more processing advice and more details.

4 Information for the Instructors

4.1 Additional Experimental Notes

Standard equipment was used for weighing in the reaction components: a fine balance, thin weighing paper and spatulas were required. It is recommendable to use small cut-outs of weighing paper and tweezers to decrease electrostatic effects. An anti-electrostatic gun can be used additionally (Figure S 4).

High precision in weighing in the catalyst components is required to ensure reproducibility (max error ±0.2 mg for catalyst components). In case of a large number of students, limited fine balances or lower precision balances, it might be favorable to prepare a catalyst stock solution with all catalyst components (both ligands and palladium acetate) dissolved in HFIP in a tightly sealed vial. Stirring the solution for 10 min, prior to addition to the substrate in the reaction vial is advisable. Preparing the stock solution for at least one excess reaction is furthermore recommended to ensure sufficient amount of solvent (avoid problem of drying out, "dead volume" in syringe, etc).

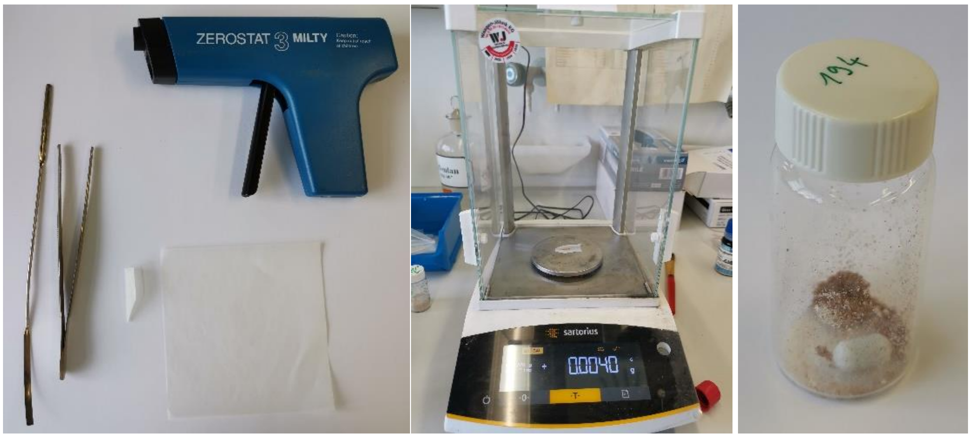

Figure S 4: Useful equipment for weighing in the reaction components and a suitable vial for the stock solution.

The following pressure resistant vials (Figure S 5) were used together with a strong neodymium stirring bar (! do NOT pierce the rubber seal for adding the stock solution as this causes HFIP leakage). As long as sufficiently strong, uniform stirring can be assured, a conventional stirring bar is suitable as well.

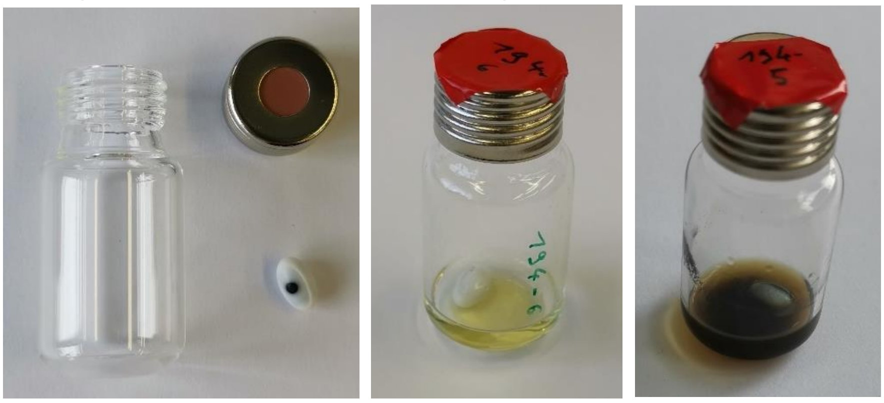

Figure S 5: Pressure resistant vials with reaction mixture before and after the reaction.

ICA stirrers with a temperature sensor (Figure S 6) and a silicon oil bath were used. The oil bath was preheated to 80°C and the temperature had stabilized prior to the reaction.

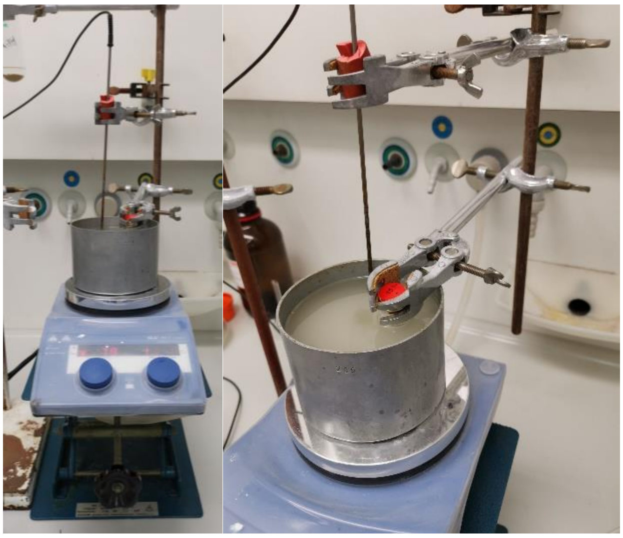

Figure S 6: Reaction set up.

The internal standard can be added either by weighing in the respective amount or by preparing a stock solution. A sufficiently precise syringe was used to add the internal standard to the reaction mixture. Figure S 7 also shows a typical pipette column that was used to avoid contaminating the GC-MS machine. In case e.g. a HRMS without prior chromatography is used, a small aliquot could be retrieved from the reaction mixture prior to addition of internal standard to avoid additional signals in the MS measurement.
For the pipette filtration a small piece of cotton or Whatman filter paper (recommended) was inserted into a glass pipette (press down with another pipette) and silica gel was added. The column was packed/equilibrated with pure ethyl acetate, the reaction mixture was applied, and washed down with ethyl acetate. This can be accelerated by using external pressure using e.g. a syringe or hand pump (Figure S 7).

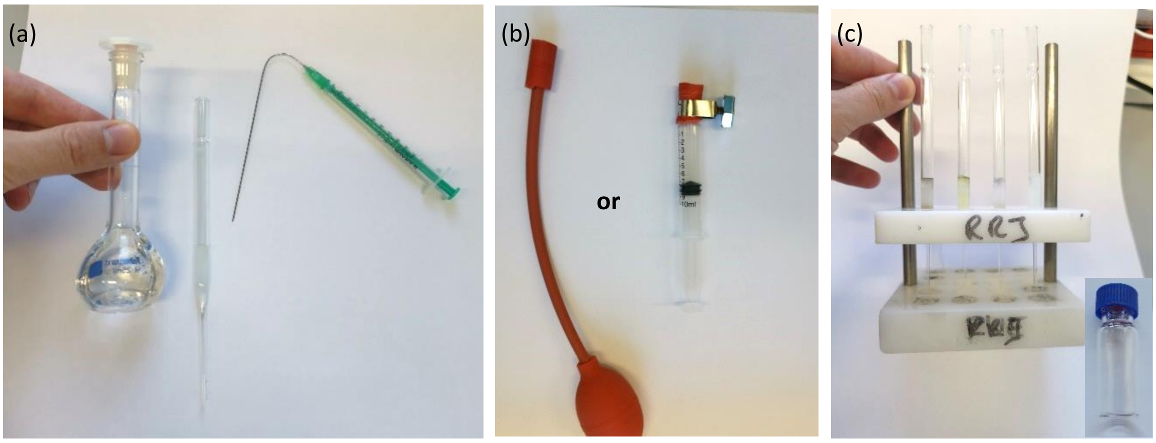

Figure S 7: (a) Volumetric flask, glass pipette with a Whatman filterpaper plug and silica (wetted with EtOAc), (b) version of a plunger for fast filtration using a syringe or a hand operated pump, (c) optional filtration stand and GC vials.

The remaining reaction mixture was filtered over a pad of celite (Figure S 8). This is best done with a funnel with an integrated frit (alternatively a cotton plug and an ordinary glass funnel can be used). The celite should be wetted with EtOAc prior to filtering the sample. After applying the sample, the filter bed should be washed carefully with several portions of EtOAc. The reaction mixture was collected in a 100 mL round bottom flask. The solvent was evaporated using a rotary evaporator and the crude product is redissolved in deuterated DMSO (ca. 0.8 mL). A suitable amount was taken up by a pipette and was transferred into an NMR tube for further analysis.

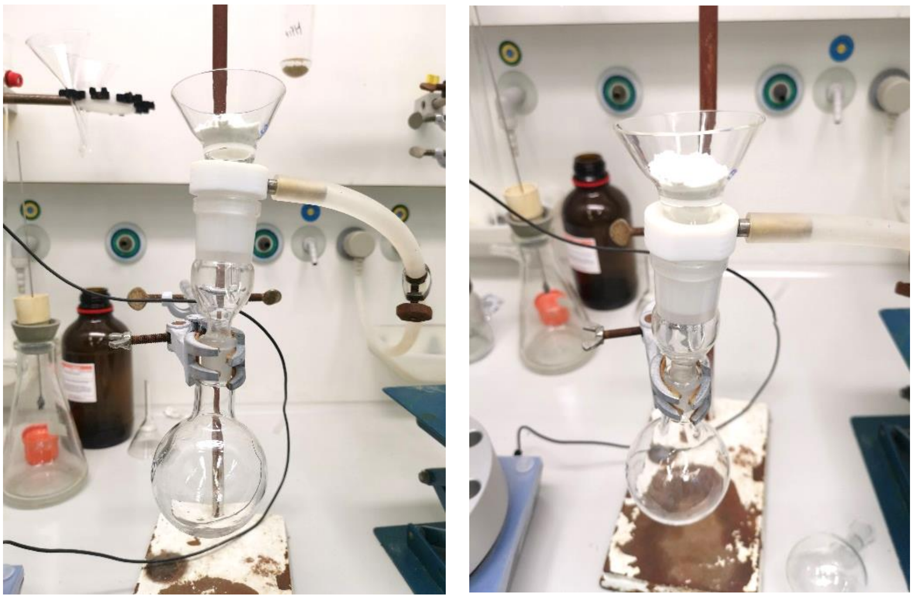

Figure S 8: Filtration over a celite pad.

Checklist of recommended material for the entire lab course in addition to the listed chemicals (see SDS)

- fine balance
- weighing paper
- scissors
- vial for stock solution
- stirring bar for stock solution
- thick neoprene gloves
- glass pipettes (long & short)
- Parafilm®
- stirring bar for stock solution
- Whatman® filter paper
- 2x erlenmeyer flasks or beakers (for aqua regia solution)
- graduated cylinder (for aqua regia solution)
- funnel
- volumetric flask (e.g. 25 mL)
- vacuum pump (for filtration)
- rotavap (+ distillation splash head, adapters, joint clips, etc, max 2 pP)
- Parafilm®
- drying oven
- permanent marker
- magnet (for retrieving stirring bars)
- acetone (for rinsing)
- containers for organic, aqueous and solid waste
- optional: ethyl acetate squeezing bottle
- optional: acetone squeezing bottle
- optional: anti-electrostatic gun
- optional: pipette holder for filtration
- optional: syringe piston (see Figure S 7) for quick filtration

Checklist of recommended material per student

- lab coat, safety goggles
- pipette tips
- silicon oil bath
- magnetic stirrer with contact thermometer
- 4x spatula
- tweezers
- 2x reactional vial

- nitrile gloves
- syringes 1 mL
- syringes 3 mL
- long needles
- 2x neodym stirring bar
- glass frit (or funnel + cotton)
- 2x 100 mL round bottom flask
- vacuum receiver, rubber gaskets, adapters, etc for filtration
- 2x NMR tube
- 2x GC vial
- sufficient stand clamps, double boss heads, stands, etc.
- 2x cork ring for round bottom flask
- 2x stopper for round bottom flask

4.2 Oral Questions prior to the Labwork

- What are the major safety concerns?
    - See SDS
- How to safely dispose of the chemicals used herein?
    - See SDS
- What analyses do you have to do?
    - See Student Information
- What is a stock solution and why can it be useful?
    - Error in weighing is not as significant as long as precise syringes are used
    - Faster set-up of several reactions
- What safety precautions do you have to take for cleaning your glassware?
    - See SDS

4.3 Possible Answers to Report Questions (Practical Part)

4.3.1 NMR Assignment of Watermelon Ketone (Compound 1-d_x) (*optional*)

Assignment of the reference compound **1** in CD_2Cl_2

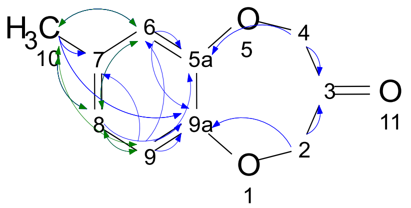

No	δ H	δ C	$J_{H,H}$ / Hz	HSQC-EDITED	HMBC	COSY
9	6.89	120.9	8.2, 0.3, 0.3	120.9(9)	121.5(6), 124.6(8),134.3(7), 146.6(9a), 148.4(5a)	2.26(10),6.77(8)
6	6.82	121.5	2.2, 0.7, 0.7	121.5(6)	20.6(10),124.6(8), 146.6(9a), 148.4(5a)	2.26(10),6.77(8)
8	6.77	124.6	8.2, 2.1, 0.7	124.6(8)	20.6(10), 121.5(6),146.6(9a)	2.26(10), 6.82(6),6.89(9)
4	4.69	76.0		76.0(4)	148.4(5a), 205.1(3)	-
2	4.66	76.3		76.3(2)	146.6(9a), 205.1(3)	-
10	2.26	20.6	0.7, 0.7, 0.3	20.6(10)	121.5(6), 124.6(8),134.3(7), 146.6(9a)	6.77(8), 6.82(6), 6.89(9)
7	-	134.3		-	-	-
9a	-	146.6		-	-	-
5a	-	148.4		-	-	-
3	-	205.1		-	-	-

The ^{13}C-NMR shifts in DMSO are comparable and provided in Figure S 9.

4.3.2 Determine Crude Yield, Degree of Deuteration and Deuterium Incorporation (Compound **1-*d*$_x$**)

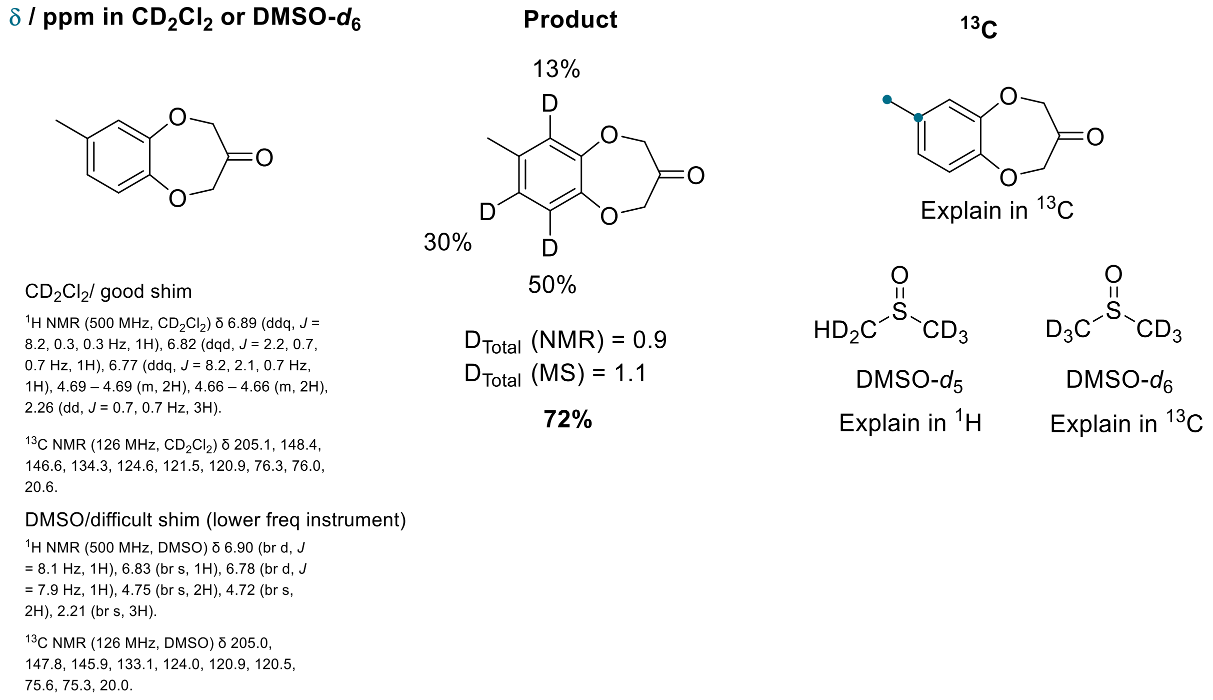

Figure S 9: Overview of the results of the deuteration of compound **1-*d*$_x$**.

Deuterium incorporation was detected (Figure S 9) and a comparison of the NMR spectra (Figure S 10) allows for the determination of crude yield (eq. 4), percent of deuterium incorporation at the respective positions (eq. 5) and overall degree of deuteration (eq. 6). The LabelChecker results (Figure S 11) indicate a similar deuterium incorporation according to EI-MS.

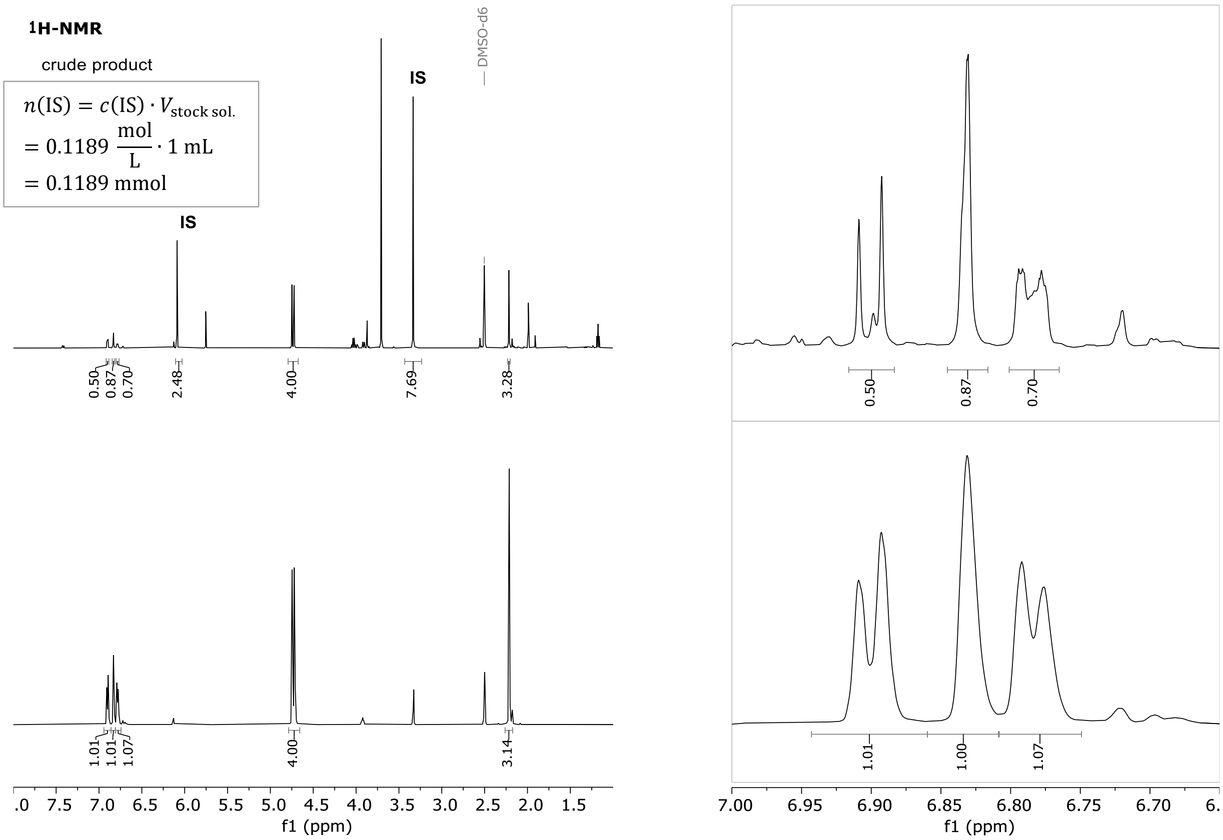

Figure S 10: Supporting stacked ^{1}H spectra.

Determination of crude yield:

$$yield\% = \frac{n(\text{IS})}{n(\text{rxn scale})} \cdot \frac{Int(\text{Product})}{Int(\text{IS})} \cdot \frac{N_{\text{eq.nuclei}}(\text{IS})}{N_{\text{eq.nuclei}}(\text{Product})} = \frac{0.1189 \text{ mmol}}{0.2 \text{ mmol}} \cdot \frac{4}{2.48} \cdot \frac{3}{4} = 0.72 = 72\% \quad (4)$$

Determination of degree of deuteration per position (results see Figure S 9):

$$D\% = 1 - \frac{N_{\text{eq.nuclei}}(\text{non-deut int. ref.})}{Int(\text{non-deut int. ref.})} \cdot \frac{Int(\text{position of interest.})}{N_{\text{eq.nuclei}}(\text{position of interest})} \quad (5)$$

$$\text{Example: } D_{6.90\text{ppm}}\% = 1 - \frac{4}{4} \cdot \frac{0.50}{1} = 0.50 = 50\%$$

Determination of total degree of deuteration (D_i% in Figure S 9):

$$D_{\text{Tot}}(\text{NMR}) = \sum_i D_i\% \cdot N_{\text{eq.nuclei}}(\text{i}) = 0.50 \cdot 1 + 0.30 \cdot 1 + 0.13 \cdot 1 = 0.93 \quad (6)$$

Equations 1-3 can be used irrespective of normalizing the integration of a nucleus of interest.

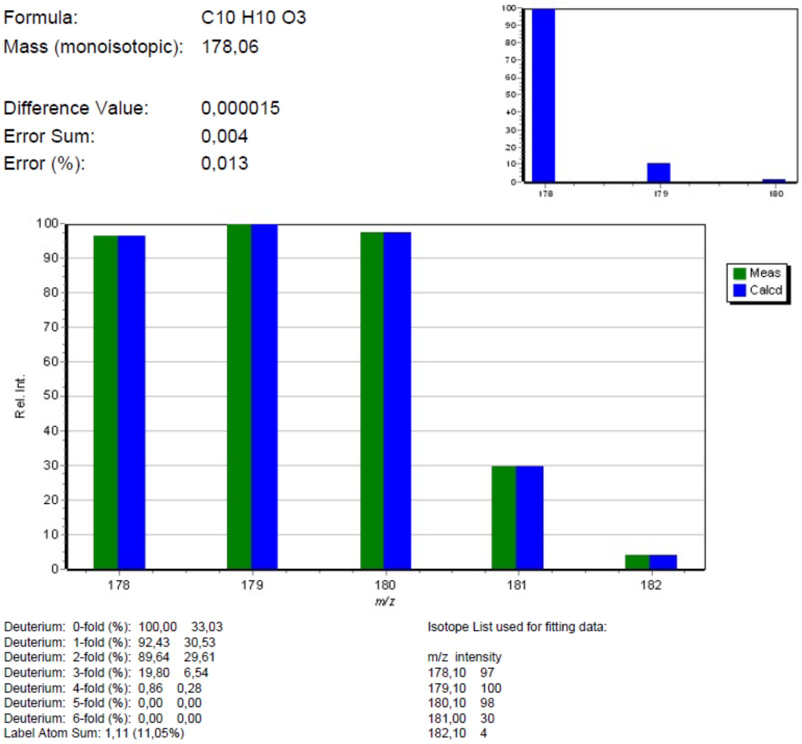

Figure S 11: Deuterium incorporation according to EI-MS for compound **2-*d*$_x$**.

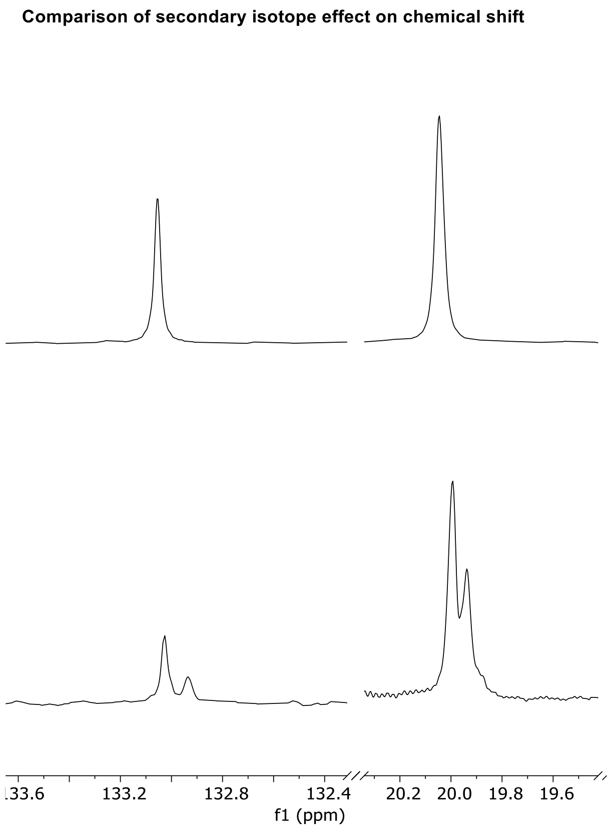

Figure S 12: Effect on ^{13}C-NMR of compound **1** and **1-*d*$_x$**.

One can observe an addition small peak adjacent to the signal of the methyl group (or the C_q) (Figure S 12). This is due to an isotope shift. Substitution with deuterium also shifts the ^{13}C resonances. In this case we observe a shift due to a substitution with two and three bonds distance, respectively. This shift is rather small and in line with literature values.[6,7]

4.3.3 Determine Crude Yield, Degree of Deuteration and Deuterium Incorporation (Compound 2-*d*$_x$)

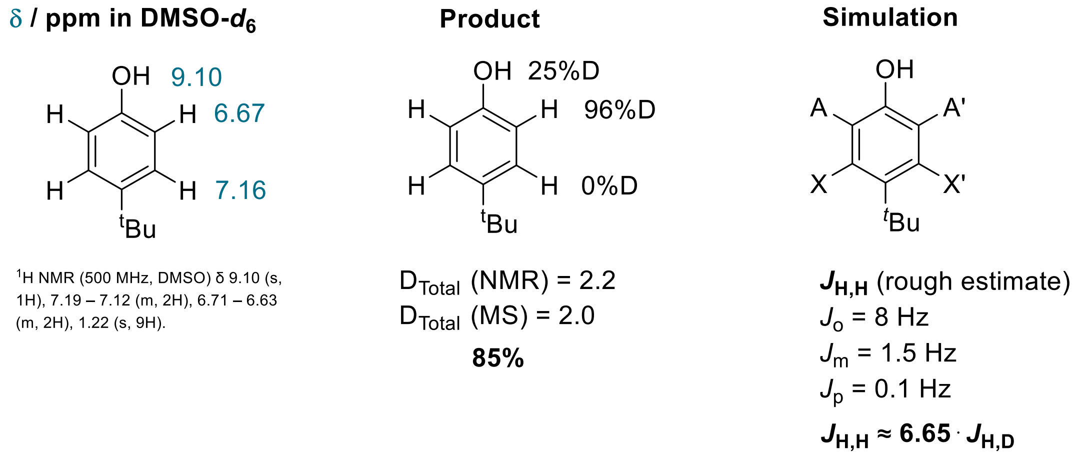

Figure S 13: Overview of the results of the deuteration of compound **2-*d*$_x$**.

Deuterium incorporation was detected (Figure S 13) and a comparison of the NMR spectra (Figure S 14) allows for the determination of crude yield (eq. 7), percent of deuterium incorporation at the respective positions (eq. 8) and overall degree of deuteration (eq. 9). The LabelChecker results (Figure S 15) indicate a similar deuterium incorporation according to EI-MS.

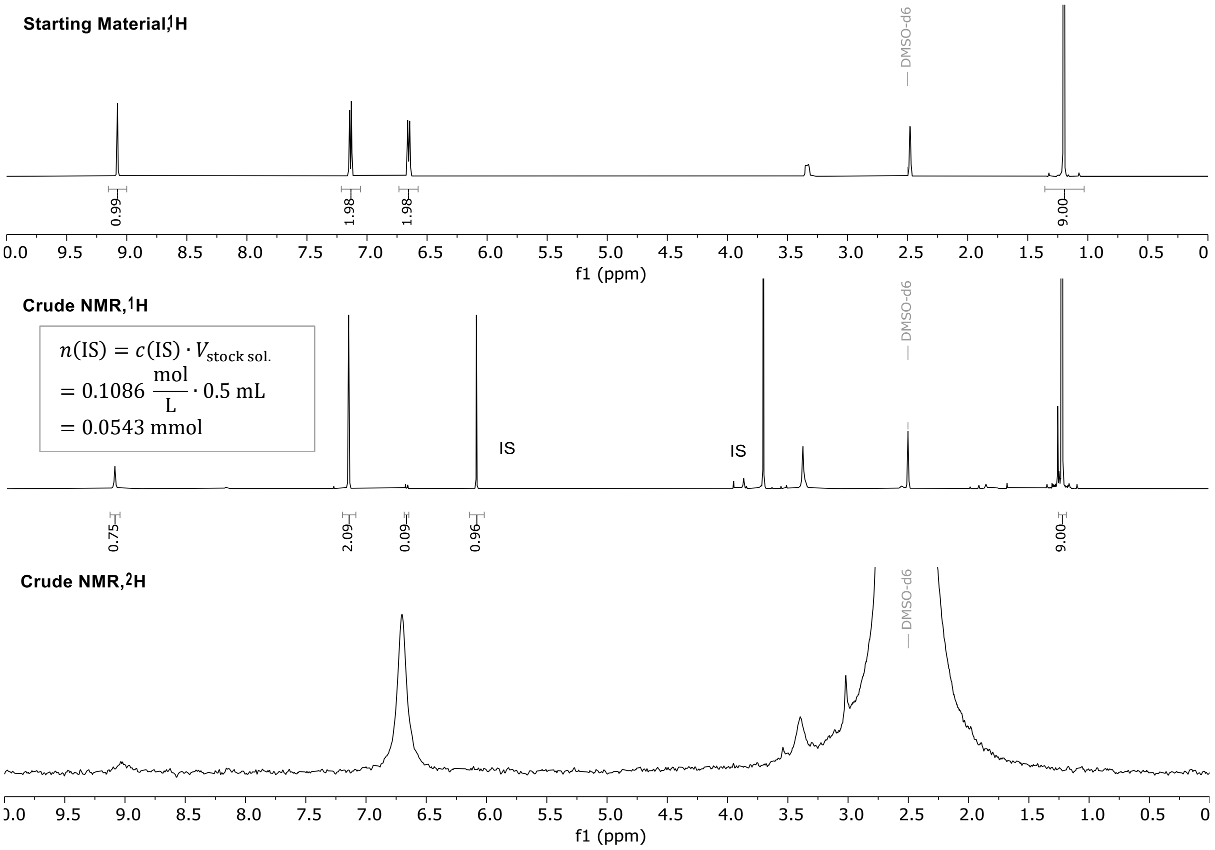

Figure S 14: Supporting stacked ^{1}H, and ^{2}H-NMR spectra.

Determination of crude yield:

$$yield\% = \frac{0.0543}{0.2} \cdot \frac{9}{0.96} \cdot \frac{3}{9} = 0.85 = 85\% \qquad (7)$$

Determination of degree of deuteration per position (results see Figure S 13):

$$D\% = 1 - \frac{N_{\text{eq.nuclei}}(\text{non-deut int. ref.})}{Int(\text{non-deut int. ref.})} \cdot \frac{Int(\text{position of interest.})}{N_{\text{eq.nuclei}}(\text{position of interest})} \qquad (8)$$

$$\text{Example: } D_{6.67\text{ppm}}\% = 1 - \frac{9}{9} \cdot \frac{0.09}{2} = 0.96 = 96\%$$

Determination of total degree of deuteration:

$$D_{\text{Tot}}(\text{NMR}) = 0.25 + 0.96 \cdot 2 + 0 \cdot 2 = 2.2 \qquad (9)$$

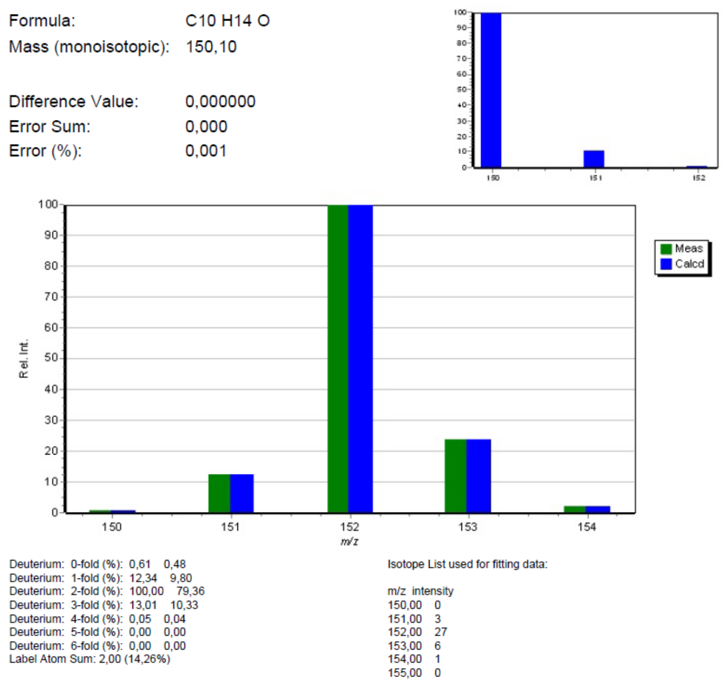

Figure S 15: Deuterium incorporation according to EI-MS.

Please note, that depending on the sampling handling different degrees of deuteration for the phenolic OH can be observed. The deuterium incorporation on the arene scaffold are rather constant.

4.3.4 Simulation of Isotopologes

The experimental chemical shift can be obtained from the ^{1}H NMR spectra. The proton-proton coupling constants are set to be J_o = 8.0 Hz, J_m = 1.5 Hz, J_p = 0.1 Hz for this exercise. The respective deuteron-proton coupling constants can be scaled with equation (10). The multiplicity can be calculated using equation (11). Please note, that the intensities for e.g. arising from a coupling with a spin = 1 rather than from two equivalent nuclei with spins = ½ are different in terms of shape. A 1:1:1 triplet instead of a 1:2:1 triplet is expected which follows Pascal's triangle.[8]

$$J_{\mathrm{H,H}} \approx 6.5 \cdot J_{\mathrm{H,D}} \tag{10}$$

$$m = 2 \cdot Spin \cdot N + 1 \tag{11}$$

The parameters used for simulation of compound **2** and **2-*d*$_x$** are indicated in Figure S 16.

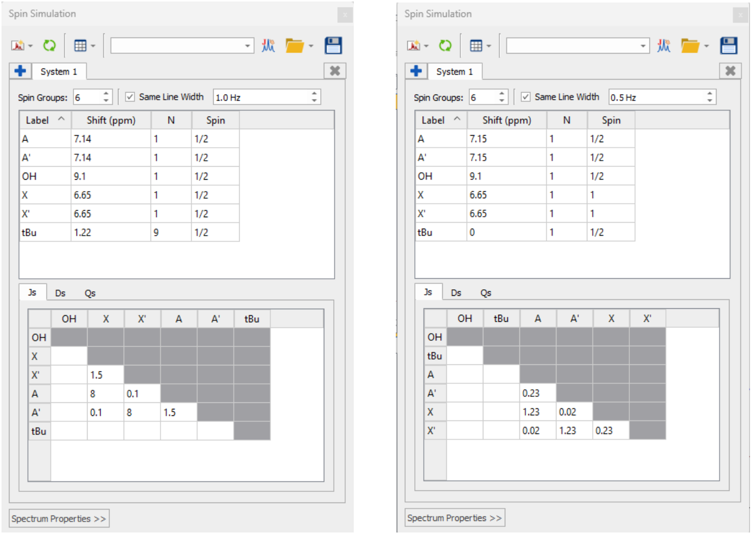

Figure S 16: Parameters used for simulation (**2** left, **2-*d*$_2$** right).

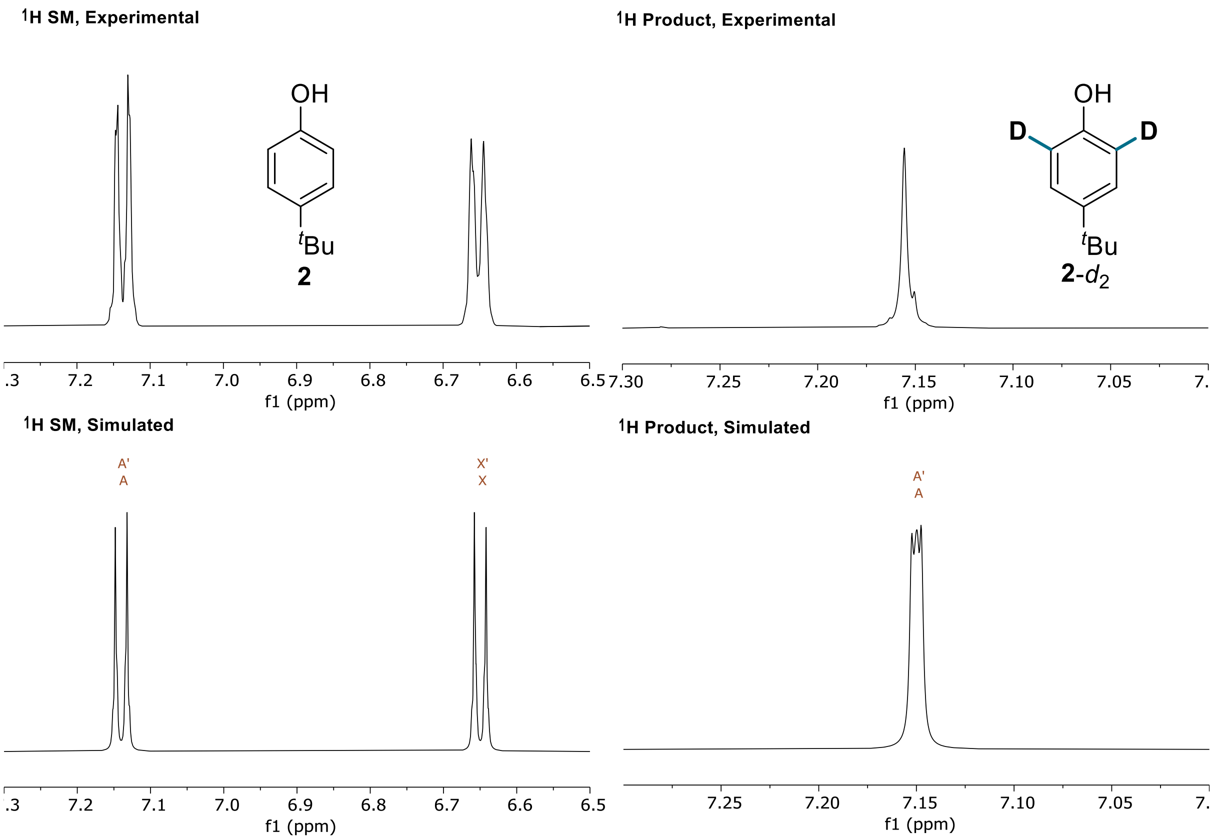

Figure S 17: Simulation of the starting material and product aromatic resonances.

The aromatic protons of Compound **2** form a higher order AA'XX' spin system. The A and A' signals have an identical chemical environment and are hence chemically equivalent but have a different coupling relation to the X or X' signals. They are therefore magnetically inequivalent. A higher order signal is expected. The simulation thereof is shown in Figure S 17. Due to the smaller $J_{H,D}$ coupling and the additional splitting (triplet vs doublet) in the experimental spectra one merely observes a broad singlet. The underlying couplings have been simulated in Figure S 17.
A high degree of deuterium incorporation *ortho* to the hydroxy group is observed. This can be seen by decrease in signal intensity in the ^{1}H NMR compared to the starting material as well as a strong peak in the ^{2}H with identical chemical shift. Some deuterium has also been incorporated in the OH group.

Additionally, the aromatic region of a tri- and tetrasubstituted benzene and representative isotopologues were simulated (Figure S 18). These are AMX and AX spin systems respectively with regard to ^{1}H. The tetrasubstituted arene shows two doublet signals, whereas the monodeuterated arene shows one triplet with 1:1:1 intensity.
In its native form the trisubstituted arene has three signals, all of which are formally dd. Due to the equivalent coupling constant of H_M and H_X on H_A (J_o = 8.0 Hz) the signal appears as a t with a 1:2:1 intensity distribution. Substituting H_X with ^{2}H results in two dt with a 1:1:1:1:1:1 intensity distribution (H_A: J = 8.0, 1.2Hz, H_B: J = 8.0, 0.2Hz). Replacing H_A with ^{2}H leads to two td with an identical intensity distribution and coupling constant (H_A: J = 1.5, 1.2Hz). Replacing H_A and H_X with ^{2}H gives a tt (J = 1.2, 0.2Hz) with 1:1:1:1:1:1 intensity distribution for H_M. In an actual spectrum this likely would only appear as a t or even a s due to the small, likely unresolved coupling constants. H_A appears to be a quintet if H_X and H_M are replaced by ^{2}H. Formally it is a tt (J = 1.2, 1.2Hz). Due to the similar coupling constant (J = 1.2 Hz) a quintet with a 1:2:3:2:1 distribution is apparent.

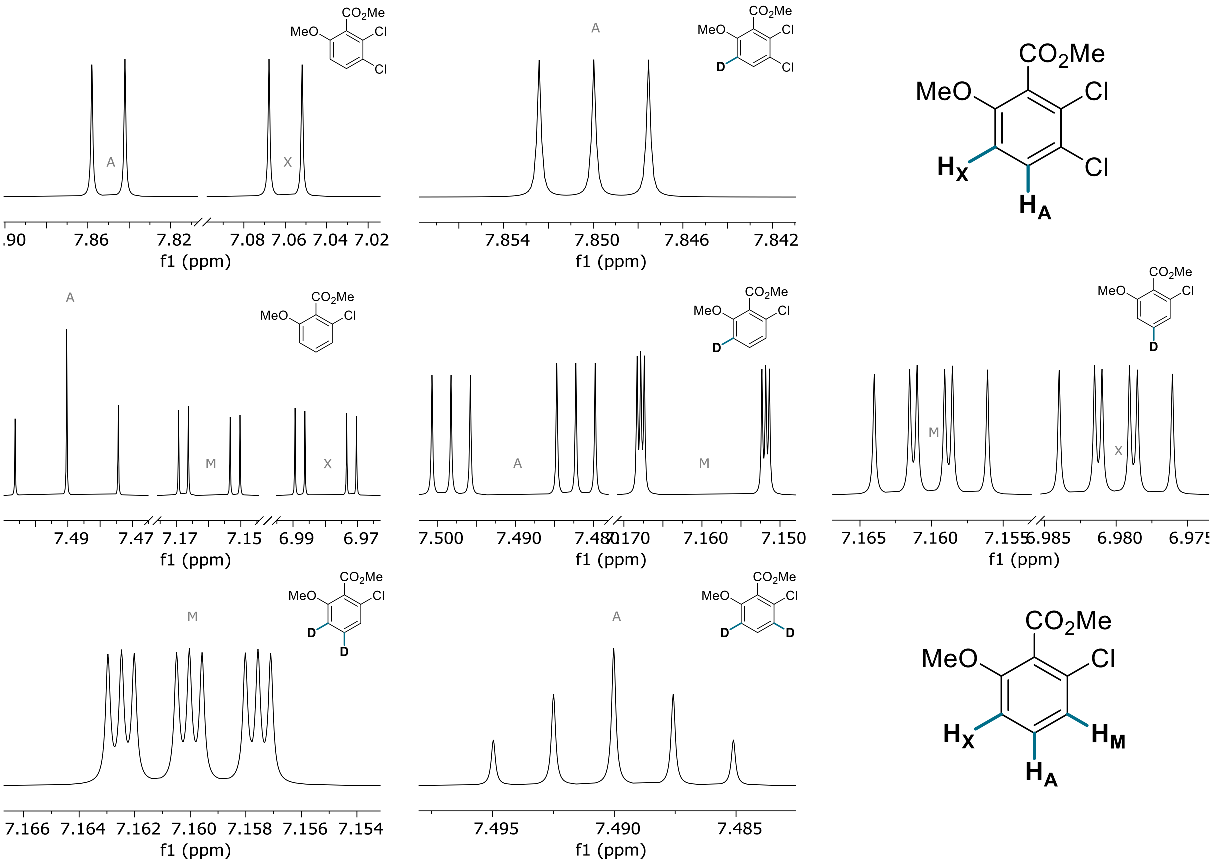

Figure S 18: Simulation of tri- and tetrasubstituted arenes and the representative isotopologues.

Please note that a quintet derived from four equivalent spin ½ nuclei has an intensity pattern of 1:4:6:4:1 (see Figure S 19).

spin = ½		spin = 1	
1	s	1	s
1 : 1	d	1 : 1 : 1	t
1 : 2 : 1	t	1 : 2 : 3 : 2 : 1	tt or quintet
1 : 3 : 3 : 1	q	1 : 3 : 6 : 7 : 6 : 3 : 1	ttt or septet
1 : 4 : 6 : 4 : 1	quintet		
1 : 5 : 10 : 10 : 5 : 1	sextet		
1 : 6 : 15 : 20 : 15 : 6 : 1	septet		

Figure S 19: Origin of the different final intensities for a quintet (spin ½), a tt (spin 1) or ttt (spin 1).

Tweaking the simulation parameters allows to directly see the influence of some experimental parameters on a hypothetical spectrum (bonus question). Increasing the linewidth (0.1 Hz -> 0.5Hz) makes apparent, that smaller coupling constants may not be properly resolved anymore (Figure S 20). Here, the tt appears like a q since signals merge. To not loose information on the coupling pattern, one can rerecord the spectra to ensure better shimming. After acquisition of the spectra a resolution enhancing weighting function can be applied during processing (! Reliability of integration could suffer).

Decreasing the amount of datapoint (512k -> 32k) results in poorly resolved quartet with 1:1:1:1 intensities but 1:3:3:1 integration. The lines are uneven and show artifacts. One can apply zero filling, linear prediction after the spectrum has been recorded to artificially increase the number of points, resulting in better resolved lines. A line broadening weighting function can improve the spectral appearance and improve integrateability. Rerecording the spectra with a longer acquisition time and/or a narrower spectral width can further result in more datapoints.

Decreasing the spectrometer frequency from 500 MHz to 50 MHz results in an apparent tilting of the signals ("roof effect"). The former AX signal becomes an AB signal (care has to be taken for reporting chemical shifts!).

Reducing the spectrometer frequency further to 5 MHz changes the signal appearance completely. A pronounced second order signal is observed.

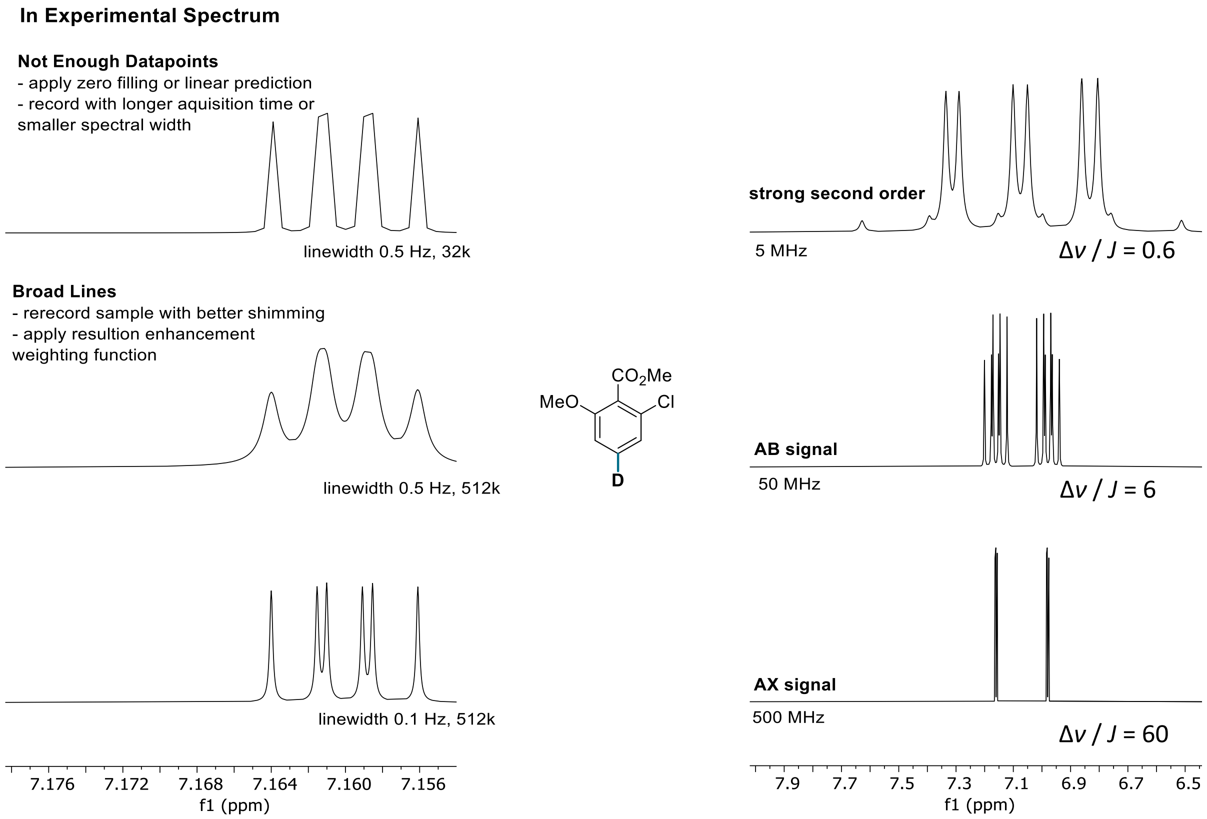

Figure S 20: Altered simulation parameters for the bonus question.

The difference in chemical shift in Hz ($\Delta\nu_{AB}$) is dependent on the spectrometer frequency. In case $\Delta\nu_{AB}$ (Hz) / $J_{A,B}$ (Hz) <5 second order effects will appear and warrant care for the determination of coupling constants and chemical shift. In case $\Delta\nu_{AB}$ (Hz) /$J_{A,B}$ (Hz) <1 the second order become detrimental for a routine analysis of the coupling pattern.[9]

4.4 Possible Answers to Report Questions (Theoretical Part)

4.4.1 Isotope Labelling

Isotopic labelling is crucial in chemical and biological/biomedical research. Metabolomics studies can e.g. rely on a deuterated compound since metabolites of said compound will also be enriched in deuterium. Deuterated compounds will in a rough approximation behave similarly to their protonated analogues, especially considering the use of another probe (e.g. replacing ^{1}H with a different atom). For metabolomics studies (often small molecules) deuteration is prevalent (other isotopes like ^{15}N, ^{13}C, etc. are used as well). Deuteration can nevertheless change the absorption, distribution, metabolism and excretion (ADME) properties. For instance, acidic OH bonds show decrease acidity and lipophilicity upon deuteration. Some deuterated compounds are investigated/marketed as drugs like e.g. Austedo (Deutetrabenazine).[10] Changes in physico-chemical properties are also interesting in material sciences e.g. for improved fluroesence lifetimes of dyes, etc.

The kinetic isotope effect can be leveraged for mechanistic studies or targeted isotope incorporation can be used e.g. for mechanistic scrambling studies in physical organic chemistry. The kinetic isotope effect relies on the principle that C–D bonds have shorter bond lengths, smaller vibrational stretching frequencies, and a lower zero-point energy. The energy required to overcome a transition barrier is hence higher in most cases leading to a pronounced k_H>k_D. The precise magnitude of the k_H/k_D allows for differentiation between primary (bond breaking) or secondary (geometric/electronic environment change) effects and hence allows to draw conclusions regarding the involved transition state.[11]

Further applications can be extracted from extensive literature reviews.[12] A potential application of a deuterated compound **1** could be metabolic studies. **1**, also known as watermelon ketone or calone could is used as an

artificial "maritime" odor in perfumes and the metabolism upon inhalation could be investigated and e.g. the cytotoxicity or environmental impact of these metabolites could be tested.[13]
Another isotope of hydrogen, namely tritium (^3H) can also be detected via ^3H-NMR. The tritium incorporation can be favorably measured via the radioactivity of the labelled target molecule. An additional method for testing ^2H incorporation is IR spectroscopy. Due to the increased mass, the vibrational frequencies are shifted towards lower wavenumbers compared to an unlabeled reference (compare equation 12-14, assuming equal force constant *k* and altered reduced mass for ^1H (μ_H) or ^2H (μ_D)).

$$\mu_{\mathrm{H/D}} = \frac{m_{\mathrm{R}} \cdot m_{\mathrm{H/D}}}{m_{\mathrm{R}} + m_{\mathrm{H/D}}} \tag{12}$$

$$\tilde{\nu}_{H/D} = \frac{1}{2\pi c}\sqrt{\frac{k}{\mu_{\mathrm{H/D}}}} \tag{13}$$

$$\frac{\tilde{\nu}_D}{\tilde{\nu}_H} = \sqrt{\frac{\mu_H}{\mu_D}} = \sqrt{0.5} \approx 0.71 \tag{14}$$

4.4.2 C–H Activation

Several different definitions of C–H activation have been used in the literature. Paraphrasing a definition originally proposed by Shilov[14], *Love et al* suggest that C–H activation is "*a specific mechanistic step involving the direct cleavage of a C–H bond that occurs due to an interaction with a transition metal, where the result is a new carbon-metal bond*." It should not be confused with C–H functionalization which is "*a process involving the replacement of a C–H bond by another element or functional group but where the functionalization is most often preceded by a C–H activation event*."[15] A C–H functionalization does not necessarily require a preceding strictly defined inner-sphere organometallic C–H activation but can also proceed via outer-sphere mechanisms like radical processes, hydrogen atom transfer (HAT), carbenoid, nitrenoid, oxoid insertion, etc (Figure S 21).[16]

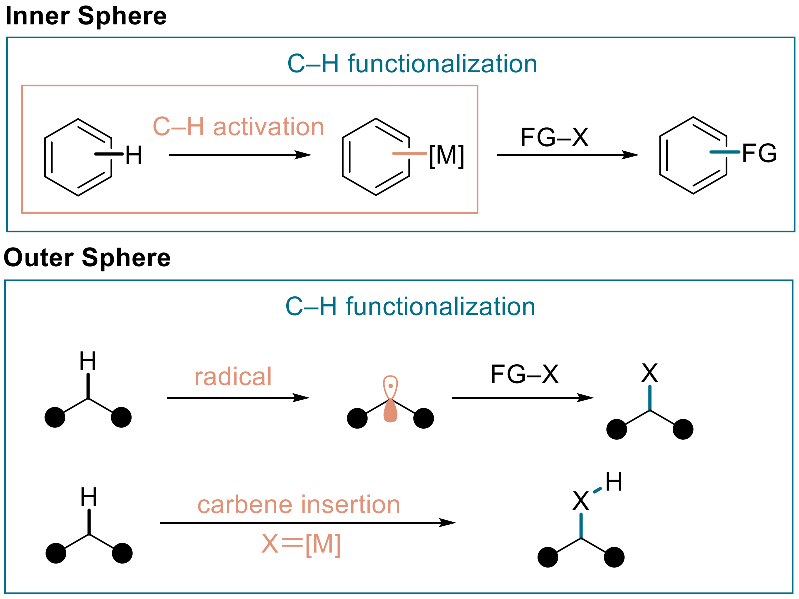

Figure S 21: Differentiation between C–H functionalization and C–H activation.[16,17]

The C–H activation reaction in this lab course is proposed to proceed as outlined in Figure S 22 based on extensive mechanistic studies on the analogous nondirected olefination of arenes.[18] It should be noted, that the C–H activation and every step in the cycle is reversible. Due to the prevalence of deuterium over hydrogen (D_2O as solvent), the equilibrium is shifted towards deuterated compound. Using a deuterated starting material in H_2O would consequently lead to de-deuteration.

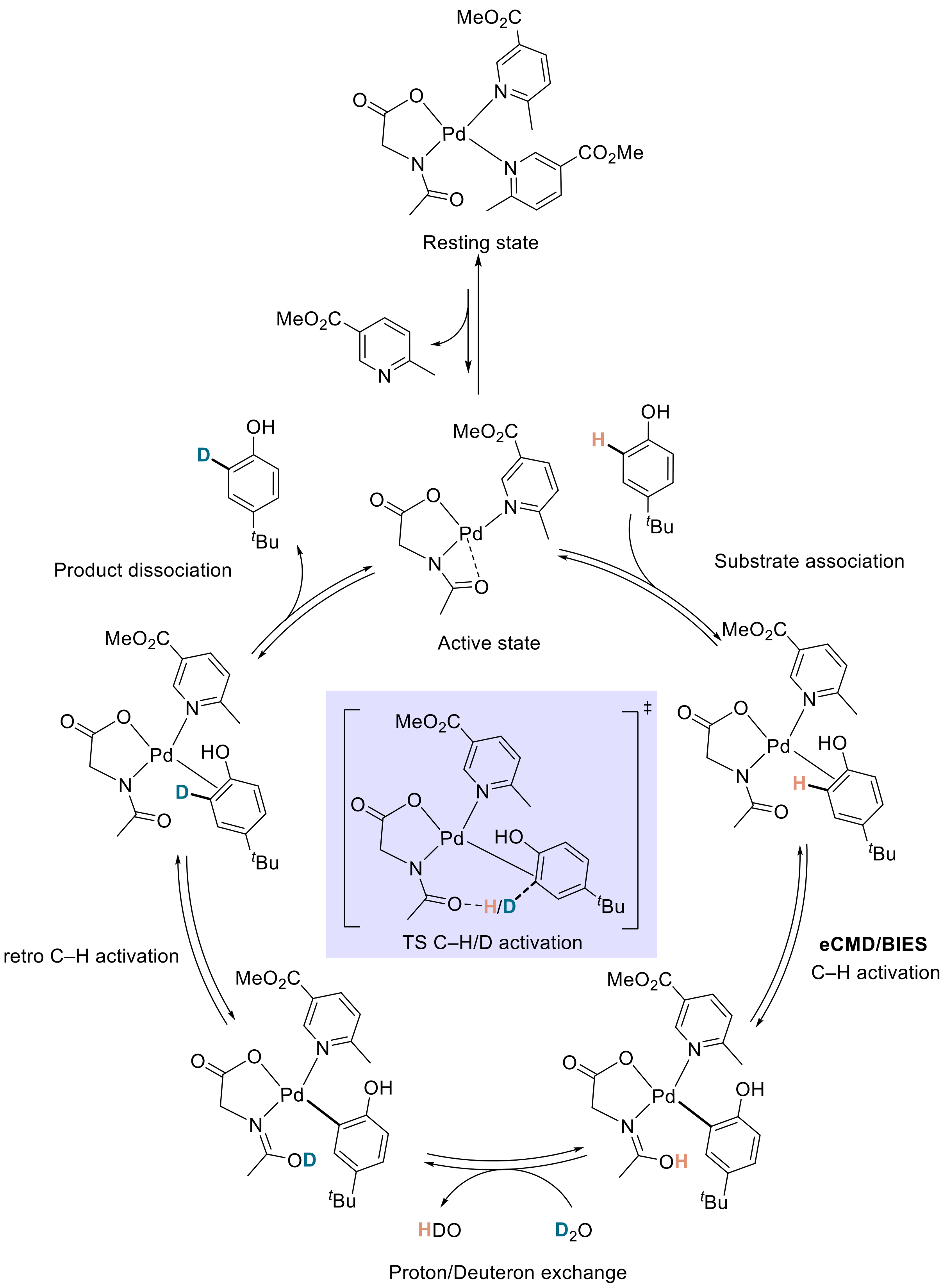

Figure S 22: Simplified proposed catalytic cycle with all reversible steps.

AcGlyOH plays a dual role as a bidentate ligand and internal base. Methyl 6-methylnicotinate is a monodentate ligand that can form a stable resting state (L:Pd = 2:1) and the active complex (L:Pd = 1:1) by dissociation of one ligand molecule. Both a required for sufficient activity as can be deducted from the optimization table of the original publication.[19]

The selectivity in this dual ligand system is governed mainly by steric and electronic effects. Sterically accessible and electron-rich positions are preferred. This is in line with a base-assisted intramolecular electrophilic-type substitution (BIES) also referred to as an electrophilic concerted metalation deprotonation (eCMD)[20] and allows us to explain the differing degrees of isotope incorporation for compounds **1** and **2** (electron rich and sterically available positions show high %D).
Contrary to a BIES one can encounter systems that preferentially functionalize the most acidic C–H bonds. These mechanisms are referred to as concerted metalation deprotonation (CMD) or ambiphilic metal-ligand activation (AMLA). Both scenarios constitute concreted mechanisms with different directions of asynchronicity. The first borderline case is an initial complete deprotonation and subsequent metalation (deprotonation/metallation mechanism). On the other extreme, a complete metalation with subsequent proton abstraction constitutes an electrophilic aromatic substitution (S_EAr) with the catalyst as electrophile. Both CMD/AMLA and BIES/eCMD are in between these extrema, which can be visualized in a More O'Ferral-Jencks diagram.[21]

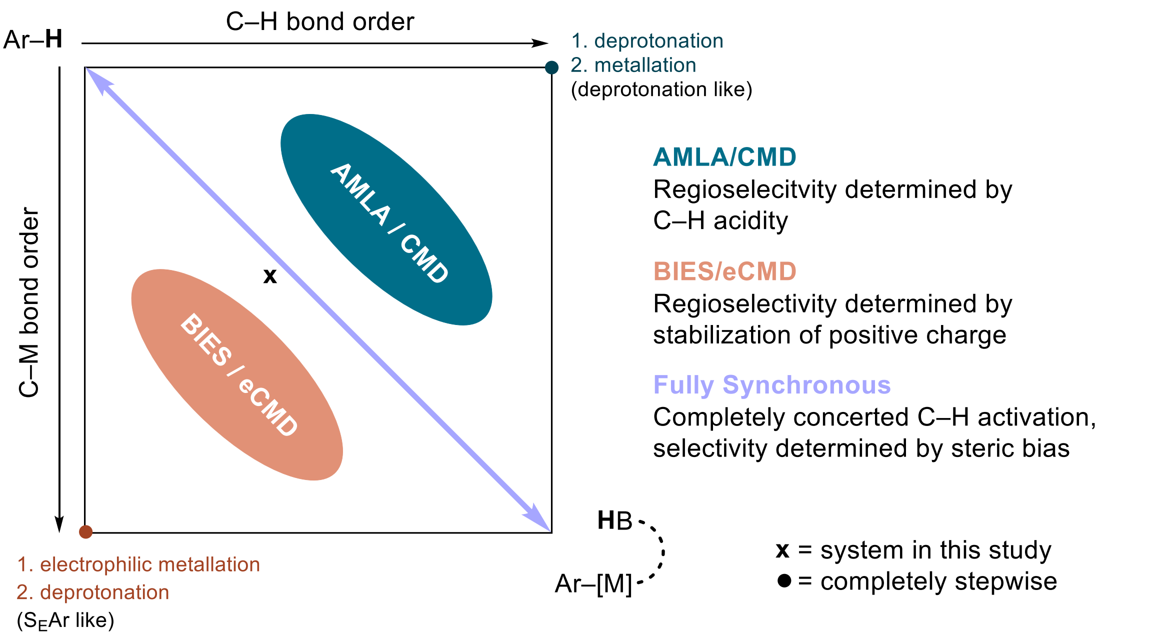

Figure S 23: More O'Ferral-Jencks diagram exemplifying the fundamental differences between CMD/AMLA and BIES/eCMD mechanisms in a mechanistic investigation from *van Gemmeren et al*.[18]

In this context, the starting materials used in this study can be analyzed. Compound **1** is a rather electron rich arene with two electron donating (+M) ether substituents. All functionalizable positions are similarly nucleophilic (see partial charges Figure S 24 a). The differences in the degree of deuteration can hence be explained mainly by differing steric bulk as evidenced by the respective A-values.[22] The position *meta* to the methyl group experiences least steric bulk and is therefore preferentially functionalized. Compound **2** is a more clear cut case since here large differences in steric bulk (^{t}Bu vs OH) and a larger electron-density *ortho* to the hydroxy group coincide such that steric and electronic factors strongly favor the same position.[VIII]

[VIII] Note for catalysis experts: We acknowledge that this compound could likely in principle be partially deuterated with similar selectivities in HFIP/D_2O in the presence of a Lewis acid as catalyst so the selectivity cannot unambiguously be attributed only to the dual-ligand catalyst system. Since the dual-ligand system alone is expected to give similar selectivities, the basis for discussion for the students remains valid. The high deuteration degrees make this compound a good candidate from a pedagogical point of view.

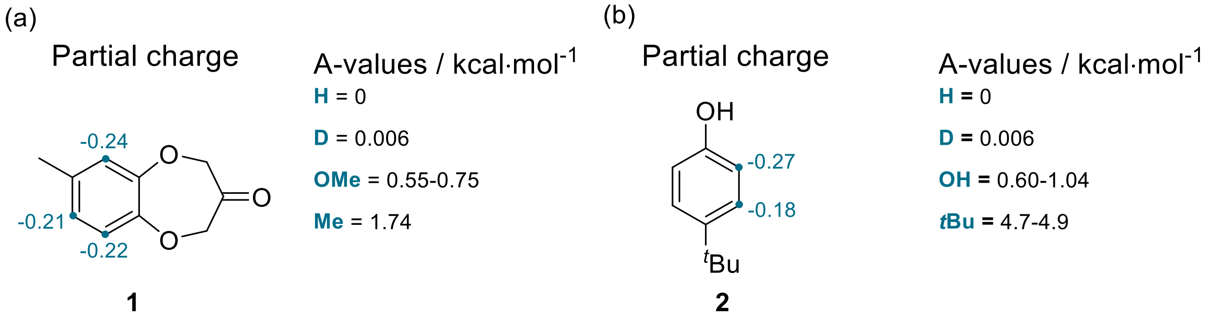

Figure S 24: Comparison of NBO partial charge and A-values for (a) Compound **1** and (b) **2**.

The method used in this lab course allows for functionalizing several positions in a molecule but without large steric bulk or electronic preferences it is not possible to selectively mono-functionalize an arene.

For a selective mono-deuteration of arenes (Figure S 25) a large manifold of literature methods has been developed and extensive reviews are available.[12,23,24] Some methods feature selective C–H deuteration mostly via directing groups, or *ortho*-metalation[25] and quenching. More generally it is possible to exchange an already existing functional handle with deuterium:

(Pseudo)halides can be converted to an organometallic compound (e.g. organolithium or organomangesium) and can subsequently be quenched with an acidic deuterium source (e.g. D_2O, AcOD, MeOD, etc.). Transition metal catalyzed reactions can be used as well to enable deuterodeborylation or deuterodehalogenation. The direct use of elemental deuterium (D_2) or an acidic, deuterated solvent are common.[12,26]

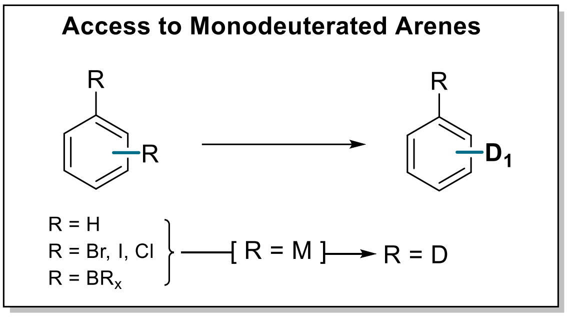

Figure S 25: Schematic overview for methods for selective mono-deuteration.

The methods described above can yield excellent regioselectivity but rely on pre-functionalized substrates rendering them unsuitable for a direct labelling of a highly complex substrate (since *de novo* synthesis with the required handle for deuteration might be required). Late-stage functionalization on the other hand is defined a as (bio)chemical, chemoselective (not necessarily regioselective) transformation on a complex scaffold to yield an analog in a synthetically useful amount and purity without requiring the introduction and subsequent removal or transformation of a functional group solely for this purpose. This is highly attractive e.g. in medicinal chemistry for rapidly generating derivatives of an active substructure without the need for *de novo* synthesis including route scouting, multistep synthesis, etc.[27,28] Mild conditions, and large functional group tolerance are crucial for late-stage functionalization.[29]

4.4.3 qNMR and NMR

In general, the following changes can be observed changing from a nondeuterated to a deuterated compound: In ^{1}H-NMR a decrease in intensity of the partially deuterated signals (or complete disappearance in case of complete deuteration) can be observed. In ^{2}H-NMR new signal are visible with a chemical shift similar to the one of the previously nondeuterated positions in ^{1}H-NMR. In ^{13}C-NMR theoretically a splitting (not always visible) can be observed since in common experiments only ^{1}H and not ^{2}H nuclei are decoupled. Additionally, an isotope induced chemical shift change is visible.

^{2}H-NMR spectra are normally recorded as NoD-NMR so without deuterated solvents since the solvent signal would otherwise be visible and could overlap with product peaks. In this case no deuterated solvent and TMS

reference is visible for referencing the spectrum. Indirect referencing via a reference in ^{1}H NMR can be used, making use of the different gyromagnetic ratio (frequency ratio) of the nuclei of interest. In reference tables of nuclei several Ξ values with the respective standard reference compound are reported.[30]

The DMSO-d_6 peak in ^{13}C NMR arises from three (N = 3) equivalent deuterons (spin n = 1), that couple to the ^{13}C nuclei: $2 \cdot n \cdot N + 1 = 2 \cdot 1 \cdot 3 + 1 = 7$ (septet, $^{1}J_{C,D} = 21.2$ Hz). Therefore, we expect a septet. Note, that the intensity distribution is different compared to a septet arising from six equivalent nuclei with a spin of 1/2. Additionally, a minor peak (quintet) arising from residual DMSO-d_5 is visible and shifted to higher ppm values. The lower chemical shift of DMSO-d_5 can be rationalized with the isotope shift as explained for compound **1**.

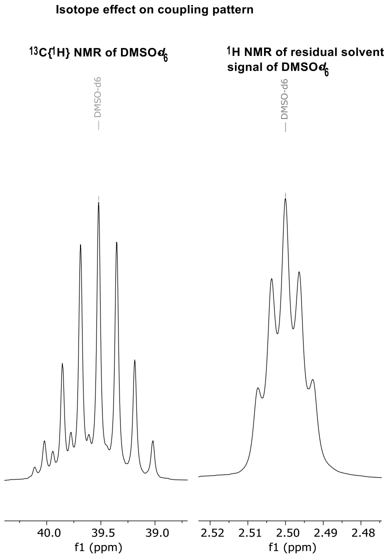

Figure S 26: (residual solvent) DMSO-d_6 signal in $^{13}C\{^{1}H\}$ and ^{1}H NMR.

The DMSO peak in ^{1}H NMR arises from residual DMSO-d_5. Here the two methyl groups are separate spin systems hence we consider an isolated CHD_2 group. A quintet is expected: $2 \cdot n \cdot N + 1 = 2 \cdot 1 \cdot 2 + 1 = 5$ with an intensity distribution due to coupling with two spin 1 nuclei (tt, $^{2}J_{H,D} = 1.8$ Hz) (compare Figure S 19). Please note that $J_{H,H} \approx 6.5 \cdot J_{H,D}$ since the ratio gyromagnetic ratios of deuterium and proton is $\gamma_H / \gamma_D \approx 6.5$.

The magnetization of all spins should return to its initial state to ensure that all spins are equally effected by the subsequent pulse and hence reliably integrateable. Due to a different chemical environment of surrounding spins, different nuclei possess different relaxation properties (T_1). More slowly relaxing nuclei will appear with a lower-than-real integration The starting material **1** for instance requires a sufficiently long relaxation delay for reliable integration (Figure S 27). These qNMR aspects might be less important for structural assignment, but are vital for the determination of deuterium incorporation and crude yield.

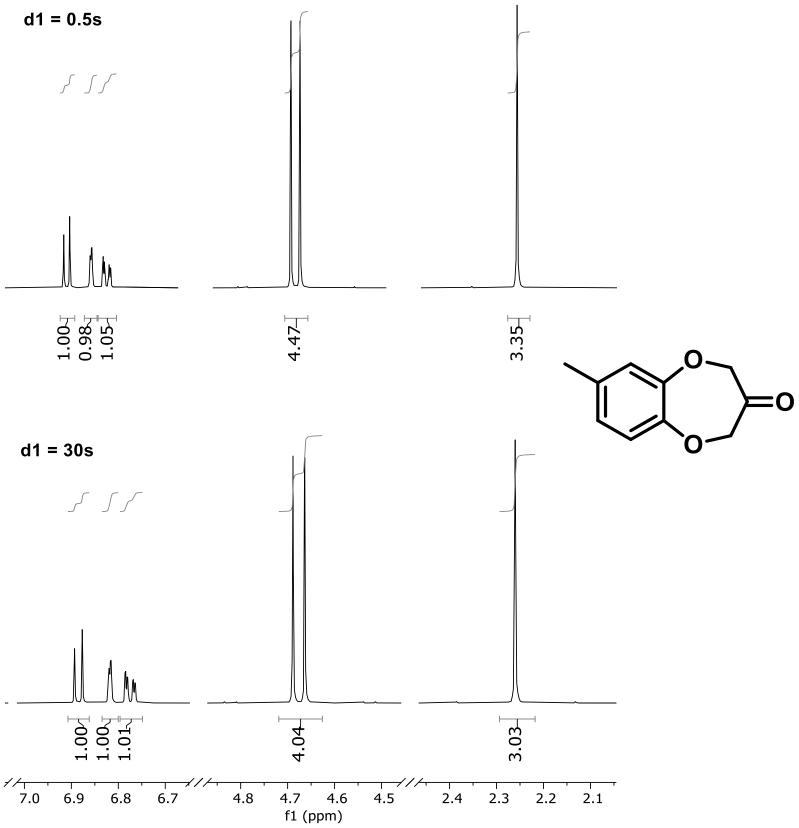

Figure S 27: Influence of relaxation delay (d1) on the integration of signals with compound **1** as an example.

Smaller pulse angles (e.g. 30°) and longer relaxation delays (d1 > 5 · T_1) can generally be recommended to achieve quantifiable results.

For compound **2** we also determined the relaxation delay needed to fully relax the respective protons (Figure S 28) and it can be easily be seen by the precise T_1 values, that a standard relaxation delay of 1s would be insufficient assuming the d1 > 5 · T_1 criteria.

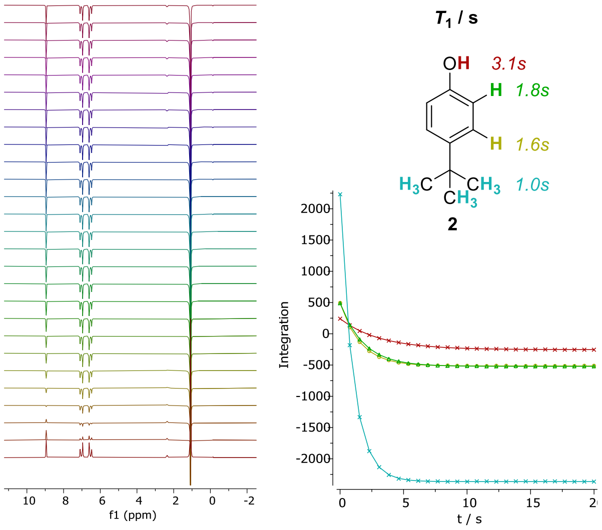

Figure S 28: Experimentally determined T_1 relaxation times for compound 2 with an inversion recovery pulse sequence.

Very long relaxation delays are required for quantitative carbon NMR $^{13}C\{^1H\}$. Additionally stronger signal to noise ratios (SNR) make the analysis less reliable. The standard decoupling sequence applies the decoupling power during the pulse sequence and the acquisition so that one benefits from additional NOE enhancement for certain signals. Since this is dependent in the proximity of other nuclei, it is not suited for quantification hence

an inverse gated decoupling scheme that only applies decoupling power during FID acquisition is better suited. Switching off the decoupler entirely would lead to a splitting due to $J_{H,C}$ coupling and hence lower SNR.
For qNMR the choice of a suitable internal standard is furthermore vital. It should be chemically inert, sufficiently fast relaxing, show only a few simple signals (no broad multiplets) with different chemical shift from product. A high enough Bp (doesn't evaporate over time) is important and an easy way to remove/separate the standard from the desired product is furthermore desirable in case the product needs to be isolated.

4.5 Additional Seminar

An accompanying lecture (outside the scope if this course) focusing on spectroscopic techniques ensured sufficient background for answering the questions and an additional seminar (within the scope of this course) was offered to answer potential questions that arose during the writing process and to recap the following important aspects (PowerPoint slides are provided in the supplemental material).

- Pople spin system nomenclature
- Pascal's triangle for spins
- NMR processing
- NMR relaxation time (T_1 & T_2)
- A plausible mechanism of the dual-ligand enabled C–H activation[18]
- Answer remaining questions
- Practical (hands-on) demonstration of spin system simulation, determination of crude yield and D_{Tot}[IX]

We carried out this lecture after the experimental lab work. We wanted to ensure an unbiased student comparison before and after the lab course. From the student feedback we concluded that the additional seminar was helpful and better suited prior to the lab work.

4.6 Useful Literature for Students and Instructors

The indicated literature references are suitable for further discussion.[6,7,15,16,18,23]

5 Student Performance and Feedback

We identified the following learning goals (LG):

LG1: Practical introduction to C–H activation, C–H deuteration, and late-stage functionalization

LG2: Determination of deuteration degrees using mass spectrometry and qNMR

LG3: Simulation of NMR spectra of deuterated organic compounds (isotopologues)

LG4: Visual determination of the parameters influencing a spectrum

5.1 Graduate Student Course and Performance

Students were paired up in groups of three to four to carry out the experimental lab work with every student performing each experiment (**LG1**). The students were tasked to write one common final report per group focusing on the analysis, interpretation, and rationalization of their individual results (**LG2**).
The lab course was designed to be carried out on two consecutive days: **Day 1**: setting up the C–H activation reaction, start preparing the report (simulation, analysis of 2D spectra, etc.), **Day 2**: work-up, cleaning and submission of the analysis.
The students required on average 7.2 h of practical time split two sessions, 3.5 h for the analysis and 13.0 h for writing the report. The students were allowed to write the reports in groups of 3-4.

The lab work was carried out in by 15 students one term so far and the results are highlighted Table S 1.

Table S 1: Average yields and deuteration degrees obtained by the students.

	# times carried out	Average yield	Std. Dev.	Average D_{Tot} (NMR)	Std. Dev.	Average D_{Tot} (MS)	Std. Dev.
Compound **1-*d*x**	15	64%	13%	1.28	0.39	1.32	0.29
Compound **2-*d*x**	15	75%	10%	2.20	0.19	1.88	0.04

Figure S 29 exemplifies the overall distribution of yields and deuteration degrees for compound **1** and **2** as specified in the students' lab reports. Outliers can be rationalized by insufficient stirring and a power outage for one stirring plate. Overall, the smaller distribution of yields and deuteration degrees for compound **2** can be

[IX] Note: This was not implemented in the reported lab course but was recommended based on student's feedback and will be implemented in future installments of the lab course

attributed to the higher solubility which limits potentially detrimental effects like insufficient stirring rate, nonuniform heating, etc. Common further error sources include unprecise weighing (of internal standard, catalyst, or substrate), volumetric measuring (solution of internal standard added volumetrically), evaporation of solvent, or differing reaction times (14-17h). The larger differences between D_{Tot} (NMR) and D_{Tot} (MS) for compound **2-*d*$_x$** can be attributed to the easily exchangeable OH position which, depending on the workup (i.e. duration of contact with acidic silica) can revert back to OH.

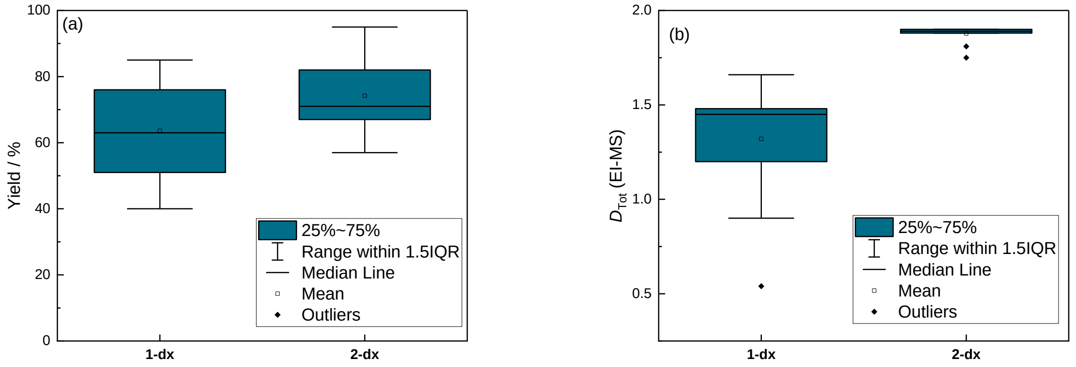

Figure S 29: Comparison of student average performance and distribution based on (a) yield and (b) D_{Tot} (EI-MS) for compound **1-*d*$_x$** and **2-*d*$_x$**.

5.2 Learning Outcomes

14 students participated in all three knowledge tests (one pre- & two post-lab tests). One student could not complete the course due to a medical condition. One additional student did not participate in the voluntary self-assessment so overall 13 students participated in both self-assessments. 14 students participated in the voluntary feedback. The experimental results and pre- and post-lab test results with the respective evaluation schemes for all students are included in the supplementary spreadsheet.

To pass the lab course and to ensure that the practical learning goals were achieved, a successful simulation of spectra and determination of deuteration degrees documented in the lab reports was mandatory and a resubmission of the reports was required in case of major flaws. All students in our cohort were able to correctly simulate spectra and determine %yield, D_{Tot}, and %D by the end of the lab course. This allowed us to verify that the practical aspects of spectral simulation and data analysis outlined in the learning outcomes were met (**LG2**, **LG3**, **LG4**). The associated theoretical understanding was also assessed in detail (*vide infra*).

5.2.1 Objective Assessment

To quantify the student's prior knowledge a test was carried out before the lab course assessing knowledge from previous lectures. The students were then given an additional test with identical questions as well as new questions after the completion of the lab course to quantify the learning outcomes of the theoretical part of the course. The categories *C–H activation* and *qNMR/Deuteration* were equally weighed (each was normalized to 10 points).

Table S 2 shows a significantly improved average performance of the students compared to the initial test. An improvement was not only visible for the repetition test but also for the new questions that were not part of the pre-lab test.

Table S 2: Student's performance in % of 100% maximal attainable points.

	Pre-lab	Post-lab (identical)	Post-lab (new)
C–H activation	26%	57%	47%
qNMR/Deuteration	23%	62%	58%
SUM	25%	60%	52%

Overall, a majority of students could improve their results, some of them by a very large margin (Figure S 30a). The distribution of results (Figure S 30b) also evidences a collective improvement of the cohort rather than only an improvement of selected individuals.

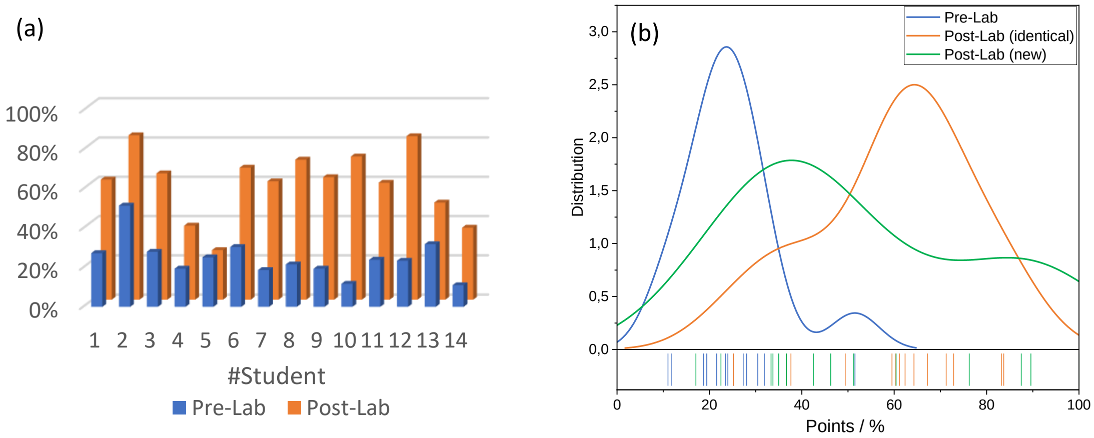

Figure S 30: (a) Comparison of the individual student's performance in the pre- and identical post-lab test as well as (b) the overall distribution of results.

With this test we assessed all theoretical aspects of **LG1**, **LG2**, **LG3** and **LG4**.

5.2.2 Subjective self-assessment

A voluntary self-evaluation of students was carried out during which they should assess their perceived knowledge and skills in specific categories. The assessment was done prior to and after the lab course.

Table S 3: Self-evaluation of students. The students could choose to assess themselves as (1) proficient, (2) rather experienced, (3) somewhat experienced, (4) little experienced, (5) no knowledge.

Question	Average pre-lab	Average post-lab	Average Improvement
How would you rank your knowledge in the theoretical aspects of C–H activation?	3.9	3.0	**0.9**
How would you rank your ability to set up an organometallic (C–H activation) reaction following a literature procedure?	2.8	2.2	**0.6**
How would you rank your knowledge in isotopic labelling?	4.2	2.9	**1.2**
How would you rank your knowledge in quantitative NMR?	3.3	2.7	**0.6**
How would you rank your knowledge in assessing the effects of deuterium on NMR spectra?	4.2	2.8	**1.4**
How would you rank your knowledge regarding virtual simulation/prediction of spin systems?	4.4	3.1	**1.3**

Overall, the students gained more confidence and subjective skills/knowledge in all of the investigated categories.

5.3 Student Feedback

The students were provided with a survey including questions regarding the laboratory course (Table S 4). Additional written feedback from the anonymous survey was given.

Table S 4: Student feedback ranging from fully agree (1), agree (2), neutral (3), disagree (4), fully disagree (5).

Question	Average Answer
The course improved my knowledge of C–H activation	**2.3**
I improved my knowledge regarding deuterium labelling	**1.9**
The additional topic seminar was useful	**1.7**
I now grasp the concept of late-stage functionalization better	**2.9**
I acquired new experimental skills regarding catalysis	**2.3**
I acquired new skills regarding analytical techniques	**1.9**
I learned/improved on how to simulate NMR coupling patterns	**1.7**
I can determine the degree of deuteration more independently	**2.4**
I understand the effect of isotope incorporation on NMR spectra better	**2.4**
I understand coupling patterns and higher order spin systems better	**2.3**
I gained more insight into the practical implications of NMR parameters like relaxation delay, decoupling, etc. on the spectrum	**2.2**
The course was manageable in the given time	**3.1**
The course was of adequate difficulty	**2.9**
Previous courses, lectures and seminars have prepared me sufficiently for the lab course	**3.4**
I overall consider the course to be instructive	**2.1**
I enjoyed the course	**1.9**

Generally speaking, the students enjoyed the novelty of a state-of-the art research experiment, the topics of deuteration and isotope labelling with the respective analytical techniques, which is reflected by the overall good feedback. The main criticism referred to the rather time demanding report writing and somewhat unclear instructions for the report writing. We addressed these issues in the instructions presented by rephrasing instructions and marking tasks as *optional.* This slightly revised version will be used as a template for the future implementation of this lab course at our university.

5.4 Grading of Students

The quiz that was used to assess the theoretical aspects of the learning outcomes **LG1**-**LG4** and can also be used in future versions of this lab course as an additional mean to grade students' performance. The metric with which we assessed the quiz can be adapted to grade students and is detailed in the spreadsheet for lab course evaluation in the supporting material. The post-lab quiz is a fast and objective way to assess the learning outcomes with respect to theoretical knowledge, complementing the grades given for the report.

The report predominantly serves to evaluate the practical and analysis related learning goals **LG2**, **LG3**, **LG4** were met for all students. Since insufficient analysis or errors would require a resubmission of the report, the successful completion of these learning goals can be ensured. Additionally, theoretical aspects can also be assessed through the report (**LG1**-**LG4**).

The LGs can be broken down within the report (*cf.* Table S 5, left column):

LG1: The students describe the experimental set-up, discuss the potential utility of C–H activation, answer questions regarding selectivity and mechanistic origins of the C–H, C–D exchange.

LG2: The students carry out the actual experimental analysis, determine %D, D_{Tot}, %yield, compare spectra with different relaxation delays and answer questions regarding qNMR

LG3: The students do the actual spectral simulation and answer theoretical questions regarding the influence of ^{2}H on the NMR spectra.

LG4: The students investigate how different NMR parameters influence spectra by tweaking simulation parameters, answer the corresponding theoretical questions, investigate the influence of relaxation delay on spectra.

The students were asked to carry out the determination of deuteration degrees individually (**LG2**) and the spectral simulations as group (**LG3**, **LG4**) and compile everything into one report per group of students.[X]
The written group report in its entirety was graded as mandated by the course syllabus and the quality of analysis, level of detail and quality of explanations, and adequate answers to the initial questions were the major factors taken into account for grading. Aspects like experimental performance, formatting, and scientific language were considered to a lesser extent.
Students were only allowed to participate in the lab course, if they could answer the questions regarding safety prior to the experimental lab work.
We suggest the following metric (Table S 5) to grade the students' written lab reports and ensure the **LG**s are met. Please note that grading of the lab reports depends on the grading systems used in a given country/university system, so we make general recommendations on a percentile basis that can easily be converted to all typical grading systems.

[X] The report could also be written by each student separately, if an entirely individual grading is required.

Table S 5: Grading criteria for the written lab report. Optional tasks are marked in grey and can serve to earn bonus points if found suitable when implementing the lab course. The learning goals that can be assessed in the respective questions are indicated in Column 1. Column 2 specifies tasks for the report and Column 3 lists details required to obtain the maximum number of points listed in Column 4 (green). Detailed ideal answers to the tasks listed are given in sections 4.3 and 4.4. Incomplete or wrong answers result in a number of points between 0 and the indicated maximum as judged by the correctors. Additionally, we specify criteria that would cause a deduction of points up to the indicated maximum of negative points (colored in red).

LG	Task	Key Aspects	Points
**Experimental Evaluation**			**Max**
**Compound 1**			
4	Assign NMR active atoms compound 1. Indicate the correlations you used (*optional*)	- Correlations in COSY (1p), ed-HSQC (1p), HMBC (3p)<br>- Correct assignment (3p)<br>- Correct reporting of multiplicities (2p)	*10*
2	Determine crude yield and D_{Tot} for compound **1-*d*$_x$** using ^{1}H-NMR	- Correct determination of yield with calculation path (3p)<br>- Correct determination of D_{Tot} with calculation path (3p)	6
2	Determine %^{2}H (specify calculation)	- Correct determination of %^{2}H with calculation path (3p)	3
2	Determine D_{Tot} using EI-MS	- Correct determination of D_{Tot} with indicated mass patterns (3p)	3
3	Compare the ^{13}C-NMR of **1** and **1-*d*$_x$** for the methyl group and the adjacent carbon. Observations?	- Identify isotope effect on chemical shift and stack spectra (2p)<br>- Give explanation (1p)	3
1,3	Changes in ^{1}H-NMR and from the nondeuterated **1** to **1-*d*$_x$**	- Indicate observations in stacked spectra (1p)<br>- Explain decrease in signal intensity in ^{1}H NMR (2p)	3
**Compound 2**			
2	Determine crude yield and D_{Tot} for compound **2-*d*$_x$** using ^{1}H-NMR	- Correct determination of yield with calculation path (3p)<br>- Correct determination of D_{Tot} with calculation path (3p)	6
2	Determine %^{2}H and compare with ^{2}H-NMR	- Correct determination of %^{2}H in ^{1}H NMR with calculation path (3p)<br>- Stack ^{2}H NMR and compare with ^{1}H NMR signals appearance/disappearance (3p)	6
2	Determine D_{Tot} using EI-MS	- Correct determination of D_{Tot} with indicated mass patterns (3p)	3
3	What spin system and what set of signals for compound **2**?	- Correct identification of AA'XX' spin system (0.5p)<br>- Correct identification of a higher order signal (0.5p)	1
3	How does the signal theoretically change from the native to deuterated compound?	- Correct identification of the spin system and expected signal (2p)	2
**NMR Simulation**			
3,4	Simulate **2** and **2-*d*$_x$** with the experimental chemical shifts. Estimate coupling constants of **2** and use $J_{H,H} \approx 6.5 \cdot J_{H,D}$ for **2-*d*$_x$**	- Correct simulation of compound **2** and comparison with experimental spectrum (3p)<br>- Estimation of coupling constants of **2** by improving the match with the experimental spectrum of 2 (2p)<br>- Correct simulation of 2-*d*$_x$ with proper scaling of the coupling constants from **2** and comparison with the experimental spectra (3p)	8
3,4	Simulate the aromatic spin system (^{1}H NMR) for all isotopologues (Figure S 3). Change the simulation parameters with the standard settings.	- Correct simulation of all isotopolgues (2p x 7 isotopolgues) with the right chemical shift and properly scaled coupling constants<br>- Explain the multiplicities and coupling patterns (0.5p x 7 isotopolgues)<br>- Change the simulation settings and note down the observations (1.5p)<br>- Relate the simulation settings to real live NMR parameters and explain (4p)	23
**Theoretical Aspects**			**Max**
**Isotope Labelling**			
1	Why is isotopic labelling relevant? Examples for ^{2}H and other nuclei. What is compound **1** used for? Hypothetical application of deuterated compound **1**?	- ADMET Studies, metabolomics, material sciences (changed properties), etc., give at least 4 examples (2p)<br>- Compound 1 is "watermelon ketone" = odorant; use metabolomics to assess potential harm as food additive (1p)	3

1	What other techniques can be used to verify deuterium or tritium incorporation?	- IR (mass difference), radioactivity (^{3}H), and explanation (2p)	2
**C–H Activation**			
1	Give a definition of C–H activation and C–H functionalization.	- Correct definition for both clearly indicating the differences (2p)	2
1	Give a mechanistic proposal for the catalytic cycle. What is the role of AcGlyOH and the Methyl 6-methylnicotinate?	- Correct mechanistic proposal (3p)<br>- Correct role of AcGlyOH and methyl 6-methylnicotinate (1p)	4
1	Compounds **1** and **2**: why there are differing degrees of deuteration depending on the position?	- Discussion of sterics (2p) and electronics (2p) and comparison of both structures (1p)	5
1	Difference AMLA/CMD and BIES/eCMD and S_EAr and deprotonation?	- Use of More O'Ferral Jencks diagram and/or explanation (1p per mechanistic scenario). Clear differentiation of scenarios (1p) and influence on selectivity (2p)	7
1	How to achieve *selective* mono-deuteration of an arene using traditional non-C–H activation chemistry? (*optional*)	- E.g. use of halogenation -> lithiation -> quenching with deuteron source or directed methods (2p)	*2*
1	What is late-stage functionalization (LSF) and why can C–H functionalization be beneficial in general? (*optional*)	- Proper definition (1p)<br>- Benefits of LSF, give 2-4 aspects (1p)	*2*
**qNMR and NMR**			
2,4	Why is measuring ^{2}H-NMR usually done in nondeuterated solvents?	- Residual solvent signal of deuterated solvent is very large compared to solute signals (1p)	1
2,4	How can you reference your shifts if you do not have residual solvent signals (*optional*)	- Use of internal standard or rather use of universal chemical shift scale -> reference indirectly via ^{1}H NMR (1p)	1
2,4	Explain the multiplicity of the solvent (deuterated DMSO) in the ^{1}H-NMR and ^{13}C-NMR? What are the coupling constants? Why is $J_{H,H} \approx 6.5 \cdot J_{H,D}$?	- Correct explanation for origin of signal in ^{1}H NMR and coupling constant (1p)<br>- Correct explanation for origin of signal in ^{13}C NMR and coupling constant (1p)<br>- Proton Deuterium coupling constant relation (1p)	3
2,4	Why is a long relaxation delay used for quantitative NMR? Compare and stack the provided spectra of the starting material **1**	- Explain relaxtion (2p) and stack spectra and explain observations (1p)	3
2,4	What constitutes a suitable internal standard for qNMR?	- Stability, purity, non-volatility, small non-overlapping signals, etc. Indicate >3 critera (2p)	2
2,4	Why is a normal ^{13}C-NMR experiment not suitable for qNMR? What do you need to record a qNMR experiment? (*optional*)	- NOE enhancement, long relaxation delay, indicate suitable experiment for quantitative decoupling (2p)	*2*
2,4	Why is there no ^{13}C-^{1}H coupling visible in a normal ^{13}C-NMR experiment? (*optional*)	- Decoupling (1p)	*1*
	**Reason**	**Example**	**Points**
**Additional Criteria**			**Max**
1,2, 3,4	Resubmission necessary	- Incorrect determination of D_{Tot} (e.g. use external rather than internal reference), %D, %yield<br>- Several incorrect or incomplete answers in the theoretical part<br>- Wrong mechanism or apparent problems in understanding	-40
1,2	Strongly deviating yields/D_{Tot}	- due to improper laboratory work (e.g. imprecise weighing, wrong temperature, etc.) NOT due to faulty equipment	-20
	Poor scientific writing, little effort for schemes and overall appearance, formalities	- schemes of poor quality (low resolution, unprofessional appearance)<br>- severely wrong significant digits<br>- poor writing (unscientific) or difficult to follow<br>- poor formatting (no page numbers, etc.)	-15
	**Total Points** (*with optional*)		**100** (*115*)

6 Possible Deviations from the Original Experiment

6.1 Use of Optimized Reaction Conditions and Different Substrates

Focus more on C–H activation by using two set of ligands and trying to rationalize the differences. The use of only ^{1}H-NMR would be sufficient for determining the degree of deuteration (depending on the existing facilities).

Higher degrees of deuteration can be achieved by the use of the optimized reaction conditions. The ligands used under these conditions are not commercially available, but can be prepared from readily available starting materials in 1-2 steps as specified in the supporting information of the original publication.[19]

Even higher degrees of deuteration can be obtained using HFIP-d_1 instead of HFIP, which can be prepared according to procedure developed by our group (see Figure S 31).[31]

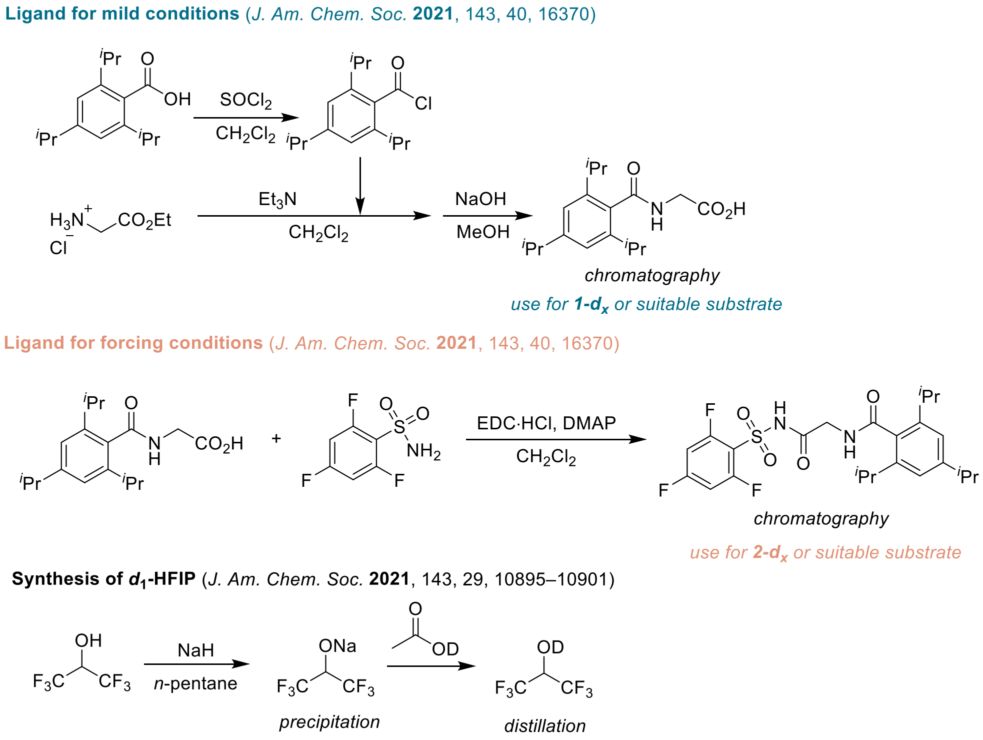

Figure S 31: Possible synthetic steps for preparing ligands in the original literature and d_1-HFIP for higher deuteration degrees.

More elaborate substrates could be used to demonstrate late-stage functionalization. In the original publication, one can find an array of, mostly commercially available late-stage structures, that could be suited for this purpose. To train multistep synthesis, a variant of this course could make use of arenes previously prepared by the students within the same lab course.

6.2 Use of Different Reaction Vessels

In our research laboratory reactions are typically performed in Schlenk redcap vials (see Figure S 32) and heated in an aluminum block with a tightly fitting recess, to which the stirring thermometer is connected. We verified for the reactions used in this lab course that it is equally possible to perform the reaction in commercial vials (see Figure S 6).

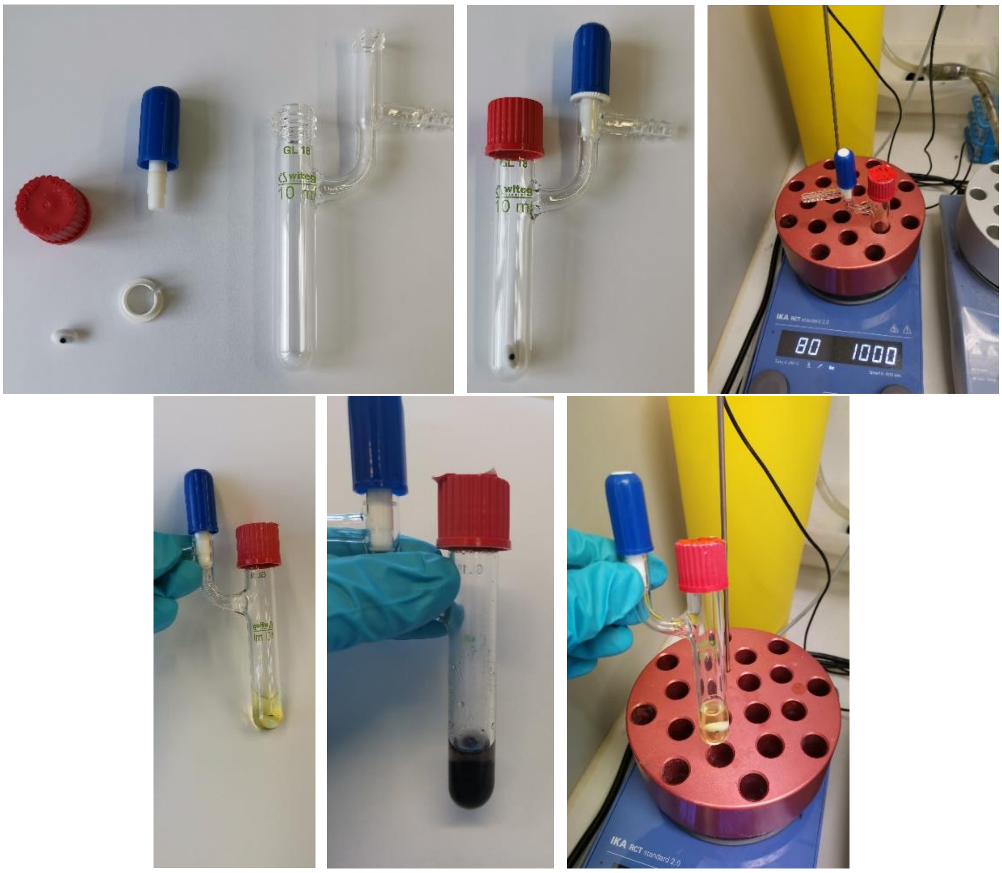

Figure S 32: Alternative reaction set-up with the reaction mixture before and after the reaction and the proper placement in the aluminum block. The stirring rate is best adjusted to 1000 rpm and the desired temperature set with a temperature sensor attached to the block.

These vials are suitable to use multiple times after cleaning with aqua regia unlike the vials described in the student lab course.

6.3 Modularity of the Course

Since the lab course is rather modular in nature, we herein propose different variations of the lab course that can enable an implementation meeting the needs of different curricula in organic chemistry. Modular components of the lab course are indicated in Figure S 33, which can be used to adjust the experiment as discussed below.

In the original lab course, the experimental work is stretched over two half days to accommodate for an 18h reaction time. In case less time is available for the course, we recommend to remove compound **1** from the laboratory course. For compound **2** the reaction time can be shortened to 4 h and the temperature increased to 120°C (92% yield, D_{Tot} = 1.9, similar isotope incorporation). The experiment can also be performed only once per group to reduce measurement time. It is possible to do the NMR analytics for this compound on lower frequency NMR spectrometers in case a 500 MHz spectrometer as used herein is not available, even on a 60 MHz benchtop NMR.

In case an extended practical version of the lab course is desired we encourage to carry out the ligand synthesis as indicated in Figure S 31. A further possibility is to additionally deuterate a previously synthesized complex arene substrate (LSF Ar). We recommend consulting the original literature[19] to assess whether an arene of interest is expected to be a suitable substrate for deuteration.

To reduce the amount of required knowledge and adjust the course for less experienced undergraduate students, we recommend to remove the NMR simulation tasks, theoretical questions regarding organometallics, and NMR theory, focusing on NMR and MS based determination of deuteration degrees and the actual experimental work. In this variant the experiment would resemble a classical organic undergraduate experiment with slightly more advanced analytics.

As an extreme measure, it is possible to convert this lab course to an online course entirely in case the necessary instrumentation is not available or in case the students are not able to carry out the practical part of the course. With the spectral data provided as Supporting Information it is feasible to do all the analysis (especially NMR simulation) remotely.

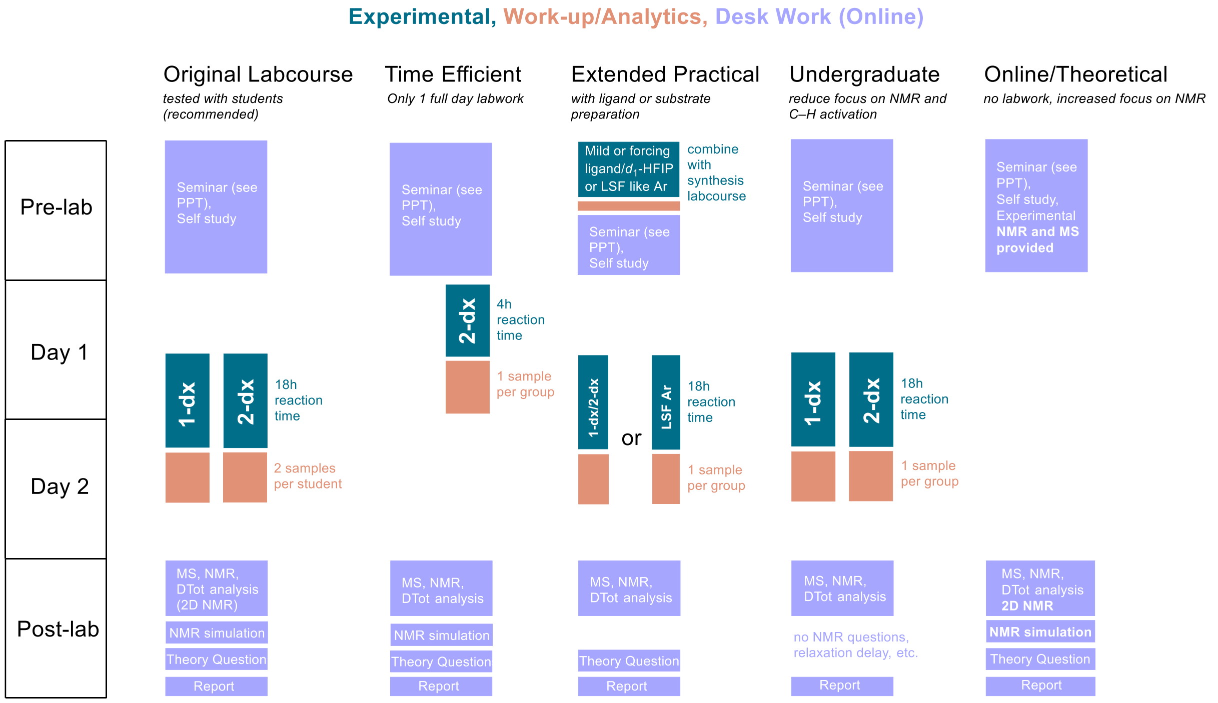

Figure S 33: Possible variations of the lab course.

7 References

(1) Fulmer, G. R.; Miller, A. J. M.; Sherden, N. H.; Gottlieb, H. E.; Nudelman, A.; Stoltz, B. M.; Bercaw, J. E.; Goldberg, K. I. NMR Chemical Shifts of Trace Impurities: Common Laboratory Solvents, Organics, and Gases in Deuterated Solvents Relevant to the Organometallic Chemist. *Organometallics* **2010**, *29* (9), 2176–2179. DOI: 10.1021/om100106e.

(2) Letzel, M. C. *Universal Mass Calculator*; WWU Münster, Org.-Chem. Institut, Germany, 2023. https://ww.uni-muenster.de/Chemie.oc/ms/downloads.html.

(3) Mestrelab Research S.L. *MestReNova*; Mestrelab Research S.L., 2019. https://mestrelab.com/download/mnova/.

(4) Bruker Corporation. *Topspin*; Bruker Corporation.

(5) Reich, H. J. WinDNMR: Dynamic NMR Spectra for Windows. *J. Chem. Educ.* **1995**, *72* (12), 1086. DOI: 10.1021/ed072p1086.1.

(6) Hansen, P. E. Isotope effects in nuclear shielding. *Prog. Nucl. Magn. Reson. Spectrosc.* **1988**, *20* (3), 207–255. DOI: 10.1016/0079-6565(88)80002-5.

(7) Hansen, P. E. Isotope Effects on Nuclear Shielding. In *Annu. Rep. NMR Spectrosc.*; pp 105–234. DOI: 10.1016/S0066-4103(08)60208-2.

(8) Lash, T. D.; Lash, S. S. The use of Pascal-like triangles in describing first order NMR coupling patterns. *J. Chem. Educ.* **1987**, *64* (4), 315. DOI: 10.1021/ed064p315.

(9) Lambert, J. B. *Organic structural spectroscopy*, 2nd ed.; Pearson Education, 2010.

(10) DeWitt, S. H.; Maryanoff, B. E. Deuterated Drug Molecules: Focus on FDA-Approved DeutetrabenazinePublished as part of the Biochemistry series "Biochemistry to Bedside". *Biochemistry* **2018**, *57* (5), 472–473. DOI: 10.1021/acs.biochem.7b00765.

(11) Anslyn, E. V.; Dougherty, D. A. *Modern physical organic chemistry*, 3rd ed.; Univ. Science Books, 2008.

(12) Kopf, S.; Bourriquen, F.; Li, W.; Neumann, H.; Junge, K.; Beller, M. Recent Developments for the Deuterium and Tritium Labeling of Organic Molecules. *Chem. Rev.* **2022**, *122* (6), 6634–6718. DOI: 10.1021/acs.chemrev.1c00795.

(13) Chapuis, C.; Cantatore, C.; Fankhauser, P.; Challand, R.; Riedhauser, J.-J. Synthesis of Deuterium-Labeled Perfume Ingredients as Internal Standards for Their GC/MS Quantification. *Helv. Chim. Acta* **2009**, *92* (9), 1782–1799. DOI: 10.1002/hlca.200900076.

(14) Shilov, A. E.; Shul'pin, G. B. Activation of C–H Bonds by Metal Complexes. *Chem. Rev.* **1997**, *97* (8), 2879–2932. DOI: 10.1021/cr9411886.

(15) Altus, K. M.; Love, J. A. The continuum of carbon-hydrogen (C-H) activation mechanisms and terminology. *Commun. Chem.* **2021**, *4* (1). DOI: 10.1038/s42004-021-00611-1.

(16) Rogge, T.; Kaplaneris, N.; Chatani, N.; Kim, J.; Chang, S.; Punji, B.; Schafer, L. L.; Musaev, D. G.; Wencel-Delord, J.; Roberts, C. A.; Sarpong, R.; Wilson, Z. E.; Brimble, M. A.; Johansson, M. J.; Ackermann, L. C–H activation. *Nat. Rev. Methods Primers* **2021**, *1* (43). DOI: 10.1038/s43586-021-00041-2.

(17) Ackermann, L. Carboxylate-assisted transition-metal-catalyzed C-H bond functionalizations: mechanism and scope. *Chem. Rev.* **2011**, *111* (3), 1315–1345. DOI: 10.1021/cr100412j.

(18) Wedi, P.; Farizyan, M.; Bergander, K.; Mück-Lichtenfeld, C.; van Gemmeren, M. Mechanism of the Arene-Limited Nondirected C–H Activation of Arenes with Palladium. *Angew. Chem. Int. Ed.* **2021**, *60* (28), 15641–15649. DOI: 10.1002/anie.202105092.

(19) Farizyan, M.; Mondal, A.; Mal, S.; Deufel, F.; van Gemmeren, M. Palladium-Catalyzed Nondirected Late-Stage C-H Deuteration of Arenes. *J. Am. Chem. Soc.* **2021**, *143* (40), 16370–16376. DOI: 10.1021/jacs.1c08233.

(20) Kaltenberger, S.; van Gemmeren, M. Controlling Reactivity and Selectivity in the Nondirected C-H Activation of Arenes with Palladium. *Acc. Chem. Res.* **2023**, *56* (18), 2459–2472. DOI: 10.1021/acs.accounts.3c00354.

(21) Carrow, B. P.; Sampson, J.; Wang, L. Base-Assisted C-H Bond Cleavage in Cross-Coupling: Recent Insights into Mechanism, Speciation, and Cooperativity. *Isr. J. Chem.* **2020**, *60* (3-4), 230–258. DOI: 10.1002/ijch.201900095.

(22) Eliel, E. L.; Wilen, S. H.; Mander, L. N. *Stereochemistry of organic compounds*; Wiley, 1994.

(23) Prakash, G.; Paul, N.; Oliver, G. A.; Werz, D. B.; Maiti, D. C-H deuteration of organic compounds and potential drug candidates. *Chem. Soc. Rev.* **2022**, *51* (8), 3123–3163. DOI: 10.1039/D0CS01496F.

(24) Atzrodt, J.; Derdau, V.; Fey, T.; Zimmermann, J. The renaissance of H/D exchange. *Angew. Chem. Int. Ed.* **2007**, *46* (41), 7744–7765. DOI: 10.1002/anie.200700039.

(25) Snieckus, V. Directed ortho metalation. Tertiary amide and O-carbamate directors in synthetic strategies for polysubstituted aromatics. *Chem. Rev.* **1990**, *90* (6), 879–933. DOI: 10.1021/cr00104a001.
(26) Modak, A.; Maiti, D. Metal catalyzed defunctionalization reactions. *Org. Biomol. Chem.* **2016**, *14* (1), 21–35. DOI: 10.1039/C5OB01949D.
(27) Wencel-Delord, J.; Glorius, F. C-H bond activation enables the rapid construction and late-stage diversification of functional molecules. *Nat. Chem.* **2013**, *5* (5), 369–375. DOI: 10.1038/nchem.1607.
(28) Zhang, L.; Ritter, T. A Perspective on Late-Stage Aromatic C-H Bond Functionalization. *J. Am. Chem. Soc.* **2022**, *144* (6), 2399–2414. DOI: 10.1021/jacs.1c10783.
(29) Dalton, T.; Faber, T.; Glorius, F. C-H Activation: Toward Sustainability and Applications. *ACS Cent. Sci.* **2021**, *7* (2), 245–261. DOI: 10.1021/acscentsci.0c01413.
(30) Harris, R. K.; Becker, E. D.; Cabral de Menezes, S. M.; Granger, P.; Hoffman, R. E.; Zilm, K. W. Further conventions for NMR shielding and chemical shifts (IUPAC Recommendations 2008). *Pure Appl. Chem.* **2008**, *80* (1), 59–84. DOI: 10.1351/pac200880010059.
(31) Uttry, A.; Mal, S.; van Gemmeren, M. Late-Stage β-C(sp3)-H Deuteration of Carboxylic Acids. *J. Am. Chem. Soc.* **2021**, *143* (29), 10895–10901. DOI: 10.1021/jacs.1c06474.
